# The organization of virtual care centers: A qualitative study in Dutch hospitals

**DOI:** 10.1371/journal.pdig.0001479

**Published:** 2026-06-26

**Authors:** Mirte A. M. Huynen, Vincent J.T. Peters, Martinus A. van Geffen

**Affiliations:** 1 Department of Information Systems and Operations Management, Tilburg University, Tilburg, The Netherlands; 2 Department of Home Monitoring, Jeroen Bosch Hospital, ‘s-Hertogenbosch, The Netherlands; University of Bremen Faculty 11 Human and Health Sciences: Universitat Bremen Fachbereich 11 Human- und Gesundheitswissenschaften, GERMANY

## Abstract

Virtual care centers (VCCs) facilitate remote patient monitoring (RPM), which has been shown to reduce secondary care utilization among patients, improve efficiency for healthcare providers, and decrease overall healthcare costs. Although VCCs are increasingly being employed, the way they are organized varies. Existing literature often conceptualizes VCCs as independent hospital wards, yet alternative organizational designs are emerging. However, there is a lack of empirical research examining how these designs differ and function. This study aims to examine and characterize the organizational designs of VCCs to provide a clearer understanding of their structure and implications. This qualitative, exploratory study, examined three VCCs in the Netherlands, selected through purposive sampling based on the volume of patients receiving virtual care and the range of care pathways offered. In April and May 2024, semi-structured interviews were conducted with twelve healthcare professionals. Thematic analysis was used to analyze the interview data. Two distinct organizational designs for VCCs have emerged from the data: decentralized designs, embedded within individual hospitals, and centralized designs, which coordinate care across multiple hospitals. To explore the different characteristics of the two organizational designs, these themes were derived: 1) operational implications, 2) collaboration between healthcare providers, 3) patient-healthcare provider relationships. Decentralized VCCs promote strong regional collaboration, fostering closer professional relationships and communication between healthcare providers and patients. However, their scalability is limited due to resource constraints and localized operational frameworks. In contrast, centralized VCCs optimize resource allocation, enabling broader patient coverage, and extended accessibility yet face challenges in maintaining care integration, trust among healthcare providers, and relational continuity with patients. Both designs exhibit difficulties in achieving multidisciplinary care integration, which is crucial for effectively supporting patients with complex and chronic conditions. The findings are relevant for policy makers working with VCCs, as setting up a VCC is more complex than it may appear. This study examines decentralized and centralized designs, each with different features and outcomes, that policy makers should consider. Future research should evaluate how VCCs accommodate varying care pathways to further inform evidence-based organizational design improvements.

## Author summary

VCCs allow patients to be remotely monitored in their home environments using digital technologies. Although VCCs have become increasingly prevalent in healthcare systems, particularly in response to the COVID-19 pandemic, there is an ongoing debate about the organizational design of a VCC. Two distinct designs have emerged: decentralized VCCs, which are embedded within individual hospitals, and centralized VCCs, which operate across multiple hospitals on a national scale. Despite the growing implementation of both designs, empirical research on their organizational differences remains limited. This study addresses that gap by interviewing twelve healthcare professionals across three VCCs in the Netherlands. Understanding these differences can support evidence-informed decisions on organizing virtual care delivery more effectively.

## Background

Virtual care centers (VCCs) facilitate remote patient monitoring (RPM) at the hospital level in the Netherlands, as a response to growing challenges, including increasing care demand, decreasing healthcare provider availability, an aging population, and the rising prevalence of chronic diseases [1–5]. VCCs facilitate a mix of digital and physical care - for perioperative, postoperative, episodic, and chronic patients - through RPM. RPM uses digital technologies to collect medical and physiological data from patients outside clinical settings and transmit it to health care providers for assessment and guidance [6]. Patients enter their medical data related to their health condition in a mobile application using a (wireless) device or sensors automatically transmit the data. When their values exceed a predefined threshold, the mobile application issues a notification prompting the repetition of a measurement or introduces additional query addressing specific symptoms. Nurses staffing the VCC monitor these alerts, offering support through digital or phone appointments, thereby enabling remote care delivery.

Previous research has examined benefits of RPM, including time and cost savings [7], reduced use of secondary healthcare for patients with chronic diseases [8–11], and a decrease in hospital readmissions for patients with chronic conditions [11–14]. The effectiveness of RPM has been examined to depend on the initial capital investment and the specific healthcare context [14]. Despite these findings, existing literature conceptualizes RPM as a technological intervention, overlooking the importance of the organizational design in its implementation.

However, while VCCs are becoming common practice in hospitals, they remain underrepresented in academic literature. Existing studies have described related models of RPM at the ward level, such as clinical management centers in Portugal [15], telehealth centers in Australia [16], virtual wards in the United Kingdom [17], virtual clinics in the United States [18], and medical command centers in Sweden [19]. Furthermore, VCCs in the Netherlands are primarily shaped by clinical and administrative judgement, accelerated by the COVID-19 pandemic. As a result, the designs of VCCs are not evidence-based, although such approaches in organizational design theory have been shown to be important for ensuring patient safety and staff satisfaction [20]. Hence, this research provides the first empirical analysis of organizational designs of VCCs in the Netherlands, addressing a gap in the literature where prior work has only presented RPM at the ward level.

## Methods

### Study design

A qualitative study design was adopted for this study, using semi-structured interviews with (healthcare) professionals employed in VCCs in the Netherlands. This exploratory, qualitative study has been conducted following the consolidated criteria for reporting qualitative research (COREQ) (S1 File) [21]. The ethics review board of [Omitted for review purposes] evaluated and approved the study design.

### Recruitment and sampling

Using purposive sampling, ten VCCs in the Netherlands employing RPM were identified [22]. Three VCCs were included in our study (A, B, and C) based on two criteria. The first criterion was the number of patients receiving digital care, indicating the scope. The second criterion concerned the range of care pathways provided, defined as structured care plans that integrate clinical interventions with supportive digital services for the management of specific patient populations [23] (Table 1).

**Table 1:** Characteristics of Virtual Care Centers in the Netherlands (2025).

VCC	Patients receiving virtual care (n)	Aggregated FTE working in VCC (FTE)	Care pathways in VCC (n)
A	6,000	30,0	42
B	600	13,0	24
C	23,000	50,0	27

Note: The data were collected in 2025 and retrieved from the department head or manager for each respective VCC.

The number of care pathways was used as a proxy measure to assess the extent of RPM implemented within each VCC, with a greater number of care pathways indicating broader applicability of such monitoring across diverse patient groups. VCC department heads and managers were approached via email or telephone to invite eligible colleagues to participate in the study. The inclusion criteria specified that participants must have engaged in the development or provision of RPM by means of a VCC and should be proficient in speaking and understanding the Dutch language. A diverse group of sixteen participants with no prior relationship to the interviewer [Omitted for review purposes] was initially approached, with twelve ultimately included in the study, encompassing a wide range of professional expertise (Table 2). Data saturation was achieved when repetition in participant’s answers occurred; this indicated that the range of relevant perspectives had been captured sufficiently and more interviews were unlikely to contribute to new understanding of the research questions [24]. Written informed consent was obtained from all participants prior to their involvement in the study.

**Table 2:** Participant characteristics.

Participant	VCC	Profession	Age (years)	Gender	Interview duration (minutes)
1	A	Nurse	31	Female	41
2	A	Nurse and Project Manager	27	Female	43
3	A	Nurse	33	Female	33
4	A	Professional Nursing Manager	42	Female	34
12	A	Medical Project Manager and Cardiologist	46	Female	40
5	B	Project Manager	54	Female	37
6	B	Nurse	39	Female	42
7	B	Scientific Researcher and Nursing Manager	31	Male	42
8	C	Project Manager	43	Female	40
9	C	Project Manager	39	Female	42
10	C	Head of VCC	30	Male	42
11	C	Nurse	46	Female	27

### Data collection

Semi-structured interviews were conducted by [Omitted for review purposes] in April and May 2024 in both face-to-face and online settings, lasting between 27–42 minutes each. An interview topic guide based on scientific literature on organizational design theory [20,25–27] was used for the interviews (S2 File). Questions were categorized into two sections: the first focus on the internal functioning and organizational design of the VCC, while the second section explored its integration within each respective hospital. The interviews were audio-recorded and transcribed. In addition, a member check with all participants was performed to verify the accuracy and credibility of the transcripts, allowing participants to provide feedback [28]. Four suggestions regarding the textual content were given and incorporated. Relevant documents, such as brochures and folders were obtained from the department heads and managers after the interviews had taken place to provide a clearer understanding of the study context. These materials were solely used for contextual insights and were not explicitly included in the data analysis.

### Data analysis

The interview transcripts were analysed using ATLAS.ti. We used the thematic analysis approach by Braun and Clarke [29] to assign codes to relevant data segments and categorized them into themes. In accordance with an inductive reasoning approach, initial codes were generated from the data [30,31]. This initial coding process started with an open coding process, followed by identifying key concepts and themes within the data. Subsequently, axial coding was employed to establish connections between these initial codes. Coding consistency was ensured by using peer meetings. The initial coding of the first three interviews was conducted by [Omitted for review purposes], after which both the coding framework and the coded transcripts were independently reviewed by [Omitted for review purposes]. Any discrepancies were discussed among all authors until consensus was reached. This process resulted in a definitive coding framework, which was subsequently applied by [Omitted for review purposes] to all remaining transcripts. This coding process led to the identification of two distinct organizational designs of VCCs: a decentralized and centralized design. We then identified themes that captured the characteristics and features of these organizational designs. The first theme captured the operational implications of VCCs, while the other themes addressed their integration within the hospital(s), including topics such as collaboration between the healthcare providers and the patient-healthcare provider relationship. Subthemes were further refined to reflect their features, illustrated with quotes from the interview data. An example of the coding process for theme 2 is presented in Table 3.

**Table 3:** Example of the development theme 2 based on our thematic analysis.

Theme	Subtheme	Illustrative quote Decentralized design	Illustrative quote Centralized design
Collaboration between healthcare providers	Personal contact between colleagues	*“The biggest difference is that you have shorter communication lines when monitoring within a hospital. You see the medical specialists walking around and you work with them.”* (Participant 2 – VCC A)	*“Well, I think that those lines of communication become much longer because you do not know the colleagues from the other hospital personally. Collaborating becomes more challenging.”* (Participant 4 – VCC A)
	Trust in colleagues	*“I think there is more mutual trust, because you simply work together intensively within the same hospital.”* (Participant 2 – VCC A); *“Trust also grows more quickly between us.”* (Participant 4 – VCC A)	*“You need trust in the medical protocol and trust in the nurses who are monitoring for you, and if they are your own nurses, then that is somewhat easier. But if it is a nurse from another hospital, then there is a challenge in the trust model.”* (Participant 10 – VCC C)
	Integrated care	*“A regional [decentralized] VCC makes it easier to work with local general practitioners, physiotherapists, social workers, and everything else that comes with it. Offering one package is less easy to organize at a national [centralized] VCC, because it covers a larger area.”* (Participant 9 – VCC C)	*“A national organization forces you to negotiate with professional associations, such as the Dutch Association of Cardiologists, for example. Then there is immediately a national protocol in place.”* (Participant 12 – VCC A)

## Results

Two distinct organizational VCC designs have emerged from the data: decentralized and centralized designs. A decentralized VCC, as observed in VCC A and B, operates as an independent unit within a hospital, where nurses (who are supervised by a project manager) oversee daily patient alerts, addressing the needs of their ‘own’ patients. In contrast, a centralized VCC, exemplified by VCC C, serves patients across multiple hospitals and is staffed by nurses and medical specialists delegated from the involved hospitals, who collaboratively manage incoming alerts.

These two organizational VCC designs differ in three themes. The first theme are the operational implications, referring to the organizational processes, workflow structures, and system integration. The second theme constitutes the collaboration between healthcare providers, while the third theme incorporates the patient–healthcare provider relationships. The themes and their corresponding subthemes are identified in Table 4.

**Table 4:** Themes and subthemes across decentralized and centralized VCC designs.

Theme	Subtheme	Decentralized design	Centralized design
Operational framework	Monitoring and alerts	Patients are monitored by nurses at their respective hospital, nurses can consult with the medical specialist of the patient if necessary. Expansion in the number of patients is limited by the capacity of the individual hospital.	A front-office for non-medical alerts; medical alerts are managed by nurses and delegated medical specialists from different hospitals. Designed for a larger patient population of secondary care patients.
	Standard operating procedures	Variation in standard operating procedures across hospitals. Programs for the included care pathways are collaboratively developed within the hospital and reflect local policies. Operating hours and services limited by in-house staffing and resources.	National uniformity in standard operating procedures and programs for the included care pathways, yet challenges arise in aligning the policies across the included hospitals. Increased accessibility for patients due to extended opening hours and optimized resource utilization.
	System interoperability and data accessibility	Focus on local interoperability of systems within each hospital or healthcare unit. EHRs accessibility is available within single hospitals, but does not extend to regional healthcare providers (e.g. GPs).	Comprehensive national-level system interoperability is required to ensure seamless data exchange and uniform data accessibility across all hospitals via shared digital platforms.
Collaboration between healthcare providers	Personal contact between colleagues	Direct interaction and communication between colleagues from the VCC and the respective hospital.	Limited personal interaction with the VCC and hospitals, as collaboration often occurs remotely and VCC operates more independently.
	Trust in colleagues	High levels of trust in colleagues of the VCC. Lower levels of trust in VCC colleagues from other hospitals.	Lower levels to trust VCC colleagues from other hospitals. Trust may become more challenging to establish.
	Integrated care delivery	Focus on network care, collaboration with GPs, and regional healthcare providers.	National disciplinary collaboration: disciplines (e.g., cardiology) sharing expertise, practices, and research.
Patient-healthcare provider relationships	Personal contact between patient and healthcare provider	High levels of personal contact between the healthcare providers and patients; addressing emotional, social, and physiological challenges of patients.	Moderate to low levels of personal contact between healthcare provider and patient due to larger numbers of patients. Expected increased accessibility due to quicker patient assistance.
	Trust between patient and healthcare provider	Patients value the trust established with their familiar local providers and employees of the VCC.	Trust may become more challenging to establish.
	Sharing power and responsibility	Care delivery through VCCs fosters sharing power and responsibility; however, it introduces a potential bias for patient inclusion, as not all patients may be equally presented.	
	Relational continuity of care	Potentially enhancing relational continuity of care by ensuring seamless transitions across various healthcare providers within the same region.	More challenging to achieve relational continuity of care due to less direct contact between the patient, healthcare provider, and the VCC.

### Theme 1. Operational framework

#### Monitoring and alerts.

The RPM process and handling of alerts vary between a decentralized and centralized VCC. In decentralized VCCs, nurses within each individual hospital manage incoming alerts based on the care pathways developed within the respective hospital, tailored to local practices and patient needs. Alerts are distributed daily among the nurses based on the respective care pathway programs or urgency criteria. Nurses manage the alerts independently and have the freedom to consult with the patient’s treating medical specialist when necessary: *“We receive the alert on the worklist, which we can then forward to the specialist. This happens about 20% of the time, and for some higher priority programs, there is a daily designated timeslot. If there is something that needs to be discussed, we often just make a phone call to the (nursing) specialist, and the alert is resolved quickly.”* (Participant 1 – VCC A). In contrast, centralized VCCs employ a more governed and collaborative approach. A front-office filters out non-medical alerts, reducing the workload for clinical staff and ensuring that only relevant medical alerts are escalated. As explained by one participant: *“If the patient is unable to install a mobile application, you do not need nursing expertise. Someone who is trained in how to install mobile applications can handle it as well.”* (Participant 9). Once filtered, monitoring occurs centrally, following standardized procedures designed to ensure consistency across different hospitals and regions. Delegated nurses and specialists from various hospitals manage the alerts within the VCC and tasks can be delegated based on specialization, potentially enhancing efficiency in managing digital alerts: *“If a nurse is employed more efficiently, for example responsible for one care pathway, she can handle alerts from that pathway more quickly and effectively. So now you might be calculating that one nurse can monitor 500 patients. But maybe you will even grow to a point where one nurse can monitor 2,000 patients. So I think there could be a lot of opportunities and differences there — provided that the technology keeps up as well.”* (Participant 9 – VCC C). Centralized VCCs can demonstrate scalability advantages in terms of managing a larger patient base in absolute numbers. By optimizing resource allocation and streamlining processes, centralized VCCs can efficiently accommodate more patients: *“Once you have streamlined the processes, scalability is definitely your advantage.”* (Participant 9 – VCC C).

#### Standard operating procedures.

The care protocols and programs used by hospitals to manage different care pathways are considered as part of the standard operating procedures (SOP) of VCCs. A tension arises between digital care delivery based on programs and protocols (standardization) versus care tailored to the patient’s needs and requirements (customization). One nurse emphasized: *“In principle, it [digital care] is very much focused on programs. So, it is quite standardized in its setup.”* (Participant 3 – VCC B). For a decentralized VCC design, instances were mentioned wherein remote care included tailored care interventions that adhered to the specific needs and preferences of individual patients. As one participant emphasized: *“There is sometimes room to make adjustments. For example, a program for high blood pressure, then people have to measure four times a day at the start. You can imagine if you are 35 years and have a job, that is not easy. Then we can see if we can solve that differently.”* (Participant 4 – VCC B)*.* Decentralized SOP reflect local policies, providing flexibility yet introducing variability, while centralized VCCs aim to enhance efficiency through SOP: *“Setting up the same processes across the entire country is much more efficient. You can expand beyond just those small regions and achieve efficiency gain with that.”* (Participant 9 – VCC C). Moreover, centralized VCCs can enhance the nurses’ ability and promote greater efficiency in managing alerts. For example, if nurses assist a greater number of patients of similar care pathways, their clinical experience and expertise related to the program will increase, potentially allowing for faster responsiveness to the patient’s needs and preferences. As exemplified by one of the participants: *“A nurse at a local level might have to handle three or four programs. On a larger scale, it would be just one or two, allowing you to delve much deeper into one. You will be quicker, so more efficient. You can also provide better advice.”* (Participant 10 – VCC C). However, while centralized VCCs may promote consistency through SOP, it faces challenges in regional alignment: *“If you ultimately create one protocol, you still have seven different procedures [for each region]. It requires a lot of work to align them.”* (Participant 11 – VCC C). Moreover, geographical differences further challenge standardization in centralized VCCs: *“You hear differences in the Netherlands, because in Groningen, when someone is ‘not feeling well’, they are lying half-dead on the coach. While in Amsterdam, ‘not feeling well’ means immediately going the hospital. It varies per region how patients react.”* (Participant 8 – VCC C). Additionally, the standard operating procedures of decentralized VCCs are constrained by limited operating hours due to resource limitations, reducing accessibility for patients. Conversely, centralized VCCs offer extended opening hours, enhancing accessibility and ensuring continuity of care. As one participant emphasized: *“Additionally, because you have so many patients, a lot of alerts will come in and you will have to employ more nurses. You can then choose to implement a 7-day week or a 24-hour service model because there are enough alerts coming in to keep someone working. With 2,000 patients, you cannot really justify scheduling an evening or night shift”* (Participant 10 – VCC C).

#### System interoperability and data accessibility.

Decentralized VCCs focus on monitoring devices within their own hospital systems. In contrast, centralized VCCs face a more complex task, requiring seamless interoperability across multiple hospitals and regions. This includes aligning the EHR, monitoring devices, and other digital (communication) tools to enable efficient communication and data sharing across hospitals. As explained by one of the participants: “*They are currently working with different mobile applications, different communication modules, and different EHRs. All of that is located in different places. It is a complex challenge to bring that together.*” (Participant 10 – VCC C).

In traditional care, access to EHRs is typically confined to each respective hospital, limiting the ability to share patient information with external providers. Similarly, in decentralized VCCs, EHR accessibility remains local and often does not extend to regional care providers like general practitioners (GPs), creating barriers to seamless integration of care. However, centralized VCCs rely heavily on data sharing across hospitals to ensure uniform access to patient information. Participants from all hospitals emphasized the importance of EHR accessibility as a critical component for effective RPM. Importantly, this did not reflect missing clinical data - most patient data, including vital signs, were generally available - but rather challenges related to how and when these data could be accessed and integrated across organizational boundaries. *“As long as we do not have all information about the patient’s health status, including information regarding mental health and how the patient wants to be coached, it becomes very difficult to provide personalized care.”* (Participant 12 - VCC A).

### Theme 2. Collaboration between healthcare providers

#### Personal contact between colleagues.

In decentralized VCCs, personal contact between colleagues from the VCC and the hospital is a defining feature. Direct interaction among colleagues within the hospital fosters informal communication and collaborative problem-solving. Multiple participants highlighted this advantage: *“The biggest difference is that you have shorter communication lines when monitoring within a hospital. You see the medical specialists walking around and you work with them.”* (Participant 2 – VCC A) and *“I think it is a big difference that we personally know everyone we work with. Each program is built and evaluated personally by us, with someone from the specialty.”* (Participant 4 – VCC A). Conversely, centralized VCCs result in greater geographical distance between colleagues, as they operate across multiple hospitals and regions. A participant from a decentralized VCC expressed concerns that communication and teamwork could be more challenging in this design: *“Well I think that those lines of communication become much longer because you do not know the colleagues from the other hospital personally. Collaborating becomes more challenging.”* (Participant 4 – VCC A).

#### Trust in colleagues.

Trust emerged as an important feature for decentralized VCCs, where colleagues operate within the same hospital. As explained by two participants: *“I think there is more mutual trust, because you simply work together intensively within the same hospital.”* (Participant 2 – VCC A) and *“Trust also grows more quickly between us [colleagues].”* (Participant 4 – VCC A). In contrast, centralized VCCs may encounter challenges in establishing trust, as medical specialists rely on nurses affiliated with other hospitals, highlighting the importance of inter-organizational trust. As one participant explained: *“You need trust in the medical protocol and trust in the nurses who are monitoring for you, and if they are your own nurses, then that is somewhat easier. But if it is a nurse from another hospital, then there is a challenge in the trust model.”* (Participant 10 –VCC C)*.* Similarly, one participant emphasized: *“What you have within your own organization, within your own capabilities, has to be handed over to another organization. And then you have to trust that it goes well.*” (Participant 9 – VCC C). The reliance on other hospitals necessitates the creation of trust through protocols to realize effective collaboration.

#### Integrated care delivery.

The extent to which integrated care is delivered varies across the two VCC designs. Decentralized VCCs have been examined to facilitate regional network care: collaborating with GPs and other local primary healthcare providers to address specific regional needs. As one participant noted: *“For example, we have a palliative program for end-stage heart failure, requiring collaboration with primary care, home care, and cardiology. [...] You need to know exactly with GP and district nurse to contact. The local knowledge is crucial, making the local approach more effective.”* (Participant 12 – VCC A). This advantage has also been recognized by a participant of a centralized VCC design: *“A regional [decentralized] VCC makes it easier* *to work with local GPs, physiotherapists, social workers, and everything else that comes with it. Offering one integrated set of care provision is less easy to organize at a national [centralized] VCC, because it covers a larger area.”* (Participant 9 – VCC C). Moreover, regional primary healthcare providers play a crucial role in carrying out essential tasks: “*Sometimes the patient needs more morphine, because s/he is short of breath. That falls under the GP’s responsibility. Similarly, if the patient feels very anxious and needs reassurance, it is the district nurse who should visit.”* (Participant 12 – VCC A). However, this does not imply that centralized VCCs are incapable of achieving similar outcomes compared to decentralized VCCs. As one participant emphasized, successful integration of the technical system is key: *“While regional network care may be easier to implement for a decentralized design, a centralized design can also succeed if the technical system can support it [regional network care].”* (Participant 9 – VCC C). Centralized VCCs provide greater opportunities for promoting integrated care across disciplines, allowing for national collaboration within the same disciplines. As exemplified by the following participant: *“A national [centralized] organization forces you to negotiate with professional associations, such as the Dutch Association of Cardiologists, for example. Then there is immediately a national protocol in place.”* (Participant 12 – VCC A). Despite the distinct advantages for decentralized and centralized VCCs in delivering integrated care, realizing integration across multiple disciplines remains a challenge for both VCC designs. As exemplified by one of the participants: *“The specialist responsible for the care pathway, for example the cardiologist, knows that the patient is in monitoring, However, if the patient contacts the surgery department the next day, the surgeon has no idea that the patient is included in monitoring. This lack of awareness is the biggest risk for integrated care.”* (Participant 6 – VCC B). Moreover, another participant highlighted the lack of communication and integration among different care pathways: *“It is not really multidisciplinary. For example, a patient with COPD and diabetes said that the care* *providers do not communicate at all. We should build in that multiple programs can be combined in the mobile application.”* (Participant 11 – VCC C).

### Theme 3. Patient-healthcare provider relationships

#### Personal contact between patient and healthcare provider.

In both centralized and decentralized VCC designs, the patient-provider relationship changes compared to traditional care provision. Participants noted a reduction in direct personal contact for both VCC designs between the treating medical specialist and patient, which aligns with the objective of hybrid care provision to reduce the burden on healthcare providers: “*If, as a patient, you can trust such a virtual care center, then I can very well imagine that the distance to the treating physician or the need for the treating physician will decrease. That is also the whole goal. So, something will change in that relationship.”* (Participant 9 – VCC C). Interestingly, participants mentioned that reduced direct personal contact increases accessibility of care: *“Many patients initially are afraid for losing contact with their doctor. But eventually, they realize that through the mobile application, they can actually have quicker contact when needed.”* (Participant 11 – VCC C). Moreover, another participant emphasized the enhanced accessibility of care: *“Research has examined that telemonitoring makes the contact with the healthcare provider more accessible. This leads to a collaboration between the healthcare providers and the patient.”* (Participant 7 – VCC B). Decentralized VCCs seem to be effective in maintaining personal contact between patients and their healthcare providers. Close geographical proximity and familiarity allow patients to feel supported and heard: *“The contact, from what I hear from the patients and experience myself, is very accessible. They generally call us easily and that makes it very personal.”* (Participant 4 – VCC A). Moreover, decentralized VCCs have demonstrated to effectively address personal interactions, managing the patient’s emotional and social needs: *“Indeed, socially, how is work going or do you feel* *anxious and down about your illness. We already include those questions in certain programs.”* (Participant 2 – VCC A). Centralized VCCs are at risk of reducing the frequency and depth of personal interactions as a result of their scalability and the inclusion of a greater absolute number of patients. While efficiency gains may be achieved in centralized VCCs, this often comes at the expense of a personal relationship between patients and healthcare providers. As one participant from a decentralized design emphasized: *“I also think that the more hospitals you monitor, the more patients you have, the less personal the contact becomes.”* (Participant 2 – VCC A). This shift has also been reflected in the centralized VCC: *“Now you would have one nurse for 500 patients, and we could maybe grow to one nurse for 2000 patients. [...] You do not have your own patients anymore, that is the biggest difference.”* (Participant 9 – VCC C) and “*I think that interactions between the patient and healthcare provider are more personal for a decentralized VCC, especially when monitoring fewer patients, compared to a centralized VCC. However, within a centralized VCC, we ensure that nurses have expertise and experience with specific types of alerts, allowing patients to receive quicker assistance than in a decentralized design.”* (Participant 10 – VCC C). The potential advantage of providing quicker assistance by a centralized design may be of greater importance than establishing a patient-provider relationship: *“I think that it is more important for the patient to be helped quickly, than that s/he has to wait for a nurse with whom s/he has built a connection with. But that is an assumption.*” (Participant 10 – VCC 10). Furthermore, some participants noted that reduced levels of direct personal contact are not necessarily harmful, as it may allow healthcare providers to focus on their own expertise: *“I do think that the nursing specialist has less conversations about how things are going [chit-chat], because that is often already identified earlier. Ultimately, they might have less contact. But precisely because of that, they can really focus on their expertise.”* (Participant 2 – VCC A). Moreover, one of the participants emphasized that higher levels of direct personal contact may lead to reduced appropriate care: *“The downside of great accessibility in contact, is being too kind to the patient. You should ask yourself, is this really effective care? This is something that still requires further research.*” (Participant 7 – VCC B).

#### Trust between patient and healthcare provider.

Patients in decentralized VCCs often rely on trust established among care providers within the hospital: *“There are short lines of communication with the (nursing) specialist and also with the patient. This increases the accessibility for the patient, familiarity, and the mutual trust.”* (Participant 4 – VCC A). In centralized VCCs, remote care delivery may feel impersonal to some, reducing trust in the therapeutic alliance: *“It can feel very impersonal for the patient if you spread it [care provision] over a very large area. Then I can completely understand that the patient loses the connection or trust in it.”* (Participant 2 – VCC A). One medical specialist emphasized the trust patients place in remote monitoring, even when the involvement of the specialist is not directly visible: *“As the VCC is currently decentralized, the relationship between the patient and specialist only becomes stronger. The patient has complete trust in the monitoring, believing that I have looked into it, even when that was not the case, and it was actually handled by the VCC.”* (Participant 12 – VCC A). For centralized VCCs, one of the participants emphasized the challenge of building trust when there is no personal relationship between the patient and the nurse: *“I think that if those connections are much more distant, because you do not know each other personally, it becomes difficult. Trust, which is still essential to achieve the desired outcome, becomes harder to establish.”* (Participant 4 – VCC A).

#### Sharing power and responsibility.

For both decentralized and centralized VCCs, participants highlighted that remote patient care delivery through VCCs contributes to self-management, the perception of control, and patient responsibility: *“Yes, I think through remote monitoring, they get to know their health condition better. For example, if a patient has had a barbecue and a few beers and ate whatever he wanted, then his weight will increase the next day. In that sense, they learn from what they do and the consequences of it.”* (Participant 4 – VCC A). This increased sense of autonomy is complemented by healthcare providers having access to accurate, real-time health data rather than relying on the patients’ subjective reports: *“The doctors, also have much more reliable values, because the patient measures at home, potentially adjusting treatment policies accordingly.”* (Participant 4 – VCC A). It was consistently emphasized that participation in remote monitoring programs necessitates patients to exhibit some level of self-management and responsibility. Currently, participation in remote monitoring is voluntary. Some patients are not suitable for remote monitoring due to, for example, specific medical conditions, limited digital literacy, age-related factors, or language barriers: *“Not all patients will be eligible for remote monitoring, so you assess what level of responsibility the patient can handle and that varies.”* (Participant 7 – VCC B)*.*

#### Relational continuity of care.

In decentralized VCCs, relational continuity of care is expected to be more easily achieved by keeping handover of care within the regional network, ensuring that patients receive care from the same team of healthcare providers who are familiar with the patient’s medical history and needs. This regional approach promotes strong personal engagement and trust between patients and providers. Conversely, centralized VCCs might face challenges due to the larger scale of operations and reduced direct interaction between patients and their treating healthcare providers. The medical specialists and nurses are delegated from multiple hospitals, which makes it more challenging to preserve the personal relationships and therapeutic alliances that are essential for ensuring relational continuity of care. As emphasized by one of the participants: *“So it could be that a patient is being treated by a cardiologist in hospital A, but* *enrolled in monitoring through a centralized VCC, with their measurements being assessed by a cardiologist from hospital B. Those two cardiologists are thus not the same person, which is a disadvantage.”* (Participant 2 – VCC A).

## Discussion

This study examined the organizational designs of VCCs in the Netherlands and identified a centralized and decentralized design. The distinction between these two designs is not rigid, hybrid forms may emerge that incorporate elements of both. The findings were categorized into three themes: 1) operational implications, 2) collaboration between healthcare providers, and 3) the patient-healthcare provider relationship, which appeared across both designs.

This study addresses a research gap in the literature, as organizational designs of VCCs have not previously been empirically examined beyond RPM at the ward level [15–19]. Our study bridges operations management and digital health disciplines, as the VCC designs possess elements related to processes and coordination, both between patients and healthcare professionals. The discussion positions these findings within the broader organizational design literature, showing how they align with and extend existing concepts.

### Operational implications of VCC designs

This study shows the operational implications of two distinct organizational VCC designs and highlights a fundamental tension between localized, hospital-specific operations in decentralized VCCs and standardized processes that regulate centralized VCC designs. The empirical findings show that in decentralized VCCs, the programs for the included care pathways are collaboratively developed and evaluated within the hospital, reflecting regional standards of care and policies. In contrast, centralized VCCs leverage standardized protocols to achieve scalability, optimize resources, and enable the management of a broader patient base.

This tension mirrors a debate in logistics and distribution literature, which conceptualizes the selection of a distribution structure as a multi-dimensional trade-off influenced by an interplay of product characteristics, supply-side constraints, such as labor and land availability, and contextual factors like regional policy divergence and digital infrastructure [32]. While centralization is traditionally favored to minimize inventory costs and exploit risk-pooling effects [33], efficiency gains are not warranted. Although centralized structures aim to minimize total system costs through coordinated replenishment, the increasing number of warehouses, or VCCs in our context, can escalate coordination complexity, potentially leading to sub-optimal performance compared to decentralized models where individual warehouses remain responsive to local cost structures [34]. Consequently, decentralization can be the preferred strategic orientation when demand dispersion increases or when local responsiveness and alignment with regional regulations are prioritized [32]. Crucially, while centralization mitigates demand fluctuations, decentralization offers resilience through risk diversification, maintaining independent warehouses to safeguard against regional disruptions or supply chain failures [35].

Both organizational VCC designs are characterized by a reliance on program-based care pathways, which may be inherited from traditional care models where protocol-based care guides care delivery [36], reinforcing a siloed approach in which each discipline operates independently. While this facilitates consistency and can improve efficiency, it may constrain the ability to provide patient-centered care, limiting the flexibility to address individual patient needs [36,37]. This challenge is not unique to VCCs but aligns with broader concerns about fragmented care, in which chronic patients receive care from multiple providers and settings that do not consistently communicate or coordinate with one another [38]. This tension between standardized protocols and patient-catered care appears to reflect structural characteristics of program-based care pathways more generally, rather then stemming solely from the organizational design of VCCs.

Moreover, both decentralized and centralized organizational designs face persistent challenges related to data infrastructure. Respondents emphasized that data accessibility and interoperable digital systems are essential for the effective functioning of a VCC. These findings align with existing literature, which highlights that limited interoperability and constraints in EHR accessibility hinder benefits of digital technology such as usability, information exchange, and advanced data analytics [39]. The empirical findings and existing literature emphasize the importance of interoperability and data accessibility.

### Collaboration between healthcare providers

The findings of this study highlight variation in collaborating between healthcare providers within decentralized and centralized VCC designs. Decentralized VCCs foster intense collaboration characterized by frequent personal contact and direct communication among healthcare providers. These attributes strengthen interpersonal trust and cohesion, as staff within the hospital and VCC are familiar with one another’s work processes. In contrast, centralized VCCs introduce a more complex collaborative dynamic, where geographical proximity between healthcare providers may hinder the development of close professional relationships. Medical specialists and nurses from distinct hospitals may experience challenges in building trust and fostering effective communication. Although physical distance alone does not inherently prevent effective collaboration, our empirical results indicate that the challenges observed in centralized VCCs arise from organizational rather than geographical distance. In centralized VCCs, collaboration often involves hospitals that are more institutionally remote, partners with whom staff have limited prior contact or established working relationships. This highlights the importance of relational governance structures. Specifically, the empirical findings indicate that centralized VCCs require not only the development of trust and effective communication between organizations but also the presence of formalized inter‑organizational governance arrangements [40]. The literature further notes that, alongside relational governance, these inter-organizational structures also include contractual mechanisms [41] to ensure that various arrangements, such as finances and data management [42], are properly managed and safeguard the involved parties against potential opportunistic behaviors [43].

From a theoretical standpoint, the strong relational foundation in decentralized VCCs may support more effective integration and collaboration with regional healthcare providers. However, decentralized VCCs may face limitations in regional expansion. As the patient population increases, the personal contact that characterizes decentralized designs may become unsustainable. This theoretical constrains invites consideration of a scale threshold at which a decentralized VCC reaches the threshold where it transitions into a centralized VCC. Identifying this “cut-off” point is essential for ensuring that effective collaboration and integration are preserved during scaling-up efforts. Future research should empirically examine this ‘cut-off’ point.

A broader discussion arises concerning the implications of VCCs on integrated care. While decentralized VCCs support integrated care through expanded regional network care, centralized VCCs potentially promote integrated care through intradisciplinary collaboration on a larger, national scale. However, the findings indicate that both designs face challenges in achieving integration of multidisciplinary care. Fragmented, poorly coordinated care provision leading to inefficiencies, is particularly prevalent among patients with chronic conditions, who often require services from multiple specialists and providers [38,44]. As patients with chronic diseases represent one of the primary focus areas for RPM by VCCs, the absence of multidisciplinary collaboration in this context underscores an important gap that requires further investigation.

### Patient-healthcare provider relationships

Our findings accentuate the difference in patient-provider relationships in decentralized and centralized VCC designs. Decentralized VCCs have been examined to facilitate higher levels of personal contact and trust between patient and provider, attributes that have been examined to be foundational to a robust therapeutic alliance and affect patient satisfaction [45,46]. In contrast, centralized VCCs may reduce opportunities for direct personal interaction, potentially weakening this alliance. While centralized designs have been hailed for their ability to increase the pace of assistance based on the respondents, it is questionable whether this design actually enables quicker assistance. Furthermore, if quicker assistance is indeed achieved, it is important to consider whether this advantage outweighs the value of maintaining a personal patient-provider relationship. Another important question is whether personal contact and trust is deemed essential for virtual care provision. While decentralized designs are intended to facilitate more personal contact, this study does not show that higher levels of personal contact contribute to improved health outcomes for patients. Future research should examine whether personal contact enhances the effectiveness of care provision in VCCs or whether factors such as quicker response times have a significant impact on patient outcomes.

Existing literature provides inconclusive results on the benefits of VCCs with regard to higher levels of sharing power and responsibility. Previous research on RPM and telemonitoring, has provided evidence for higher levels of sharing power and responsibility because of RPM [47], where patients are active participants in their treatment decisions [48], whereas other research emphasized that patient empowerment is not improved by digital care provision [49]. Our findings suggest that remote monitoring through VCCs fosters the patient’s autonomy and their ability to actively engage in decision-making regarding their care provision. However, respondents also emphasized that patients must meet certain eligibility criteria to be enrolled in RPM programs. Hence, the empirical findings show that this introduces a potential selection bias, as it is difficult to determine whether the observed increase in autonomy stems from the organizational design of VCCs or from the pre-existing self-management capacities of patients who are deemed suitable for remote monitoring. This ambiguity aligns with existing literature that reports inconsistent empirical evidence regarding whether RPM genuinely redistributes power and responsibility between patients and healthcare providers.

The concept of relational continuity of care offers additional insights into the patient-health care provider relationship, particularly its relevance for trust and support in a therapeutic relationship. Relational continuity, a component of the broader concept of continuity of care, refers to an ongoing therapeutic relationship with the healthcare provider [45]. Moreover, continuity of carer - a concept in maternal healthcare that refers to care from a consistent and familiar healthcare provider - has been shown to significantly reduce mortality rates, decrease hospital admissions, and improve patient satisfaction [50,51]. However, the findings of our study suggest that the relational continuity of care may come at risk because of VCC care delivery, as patients may be unaware of the specific healthcare providers overseeing their care. Addressing this challenge is essential, particularly in centralized VCCs, where the disconnection between patients and healthcare providers may be more pronounced.

## Limitations and future research

This study is not without any limitations. First, this study only included three out of ten VCCs in the Netherlands, potentially limiting the generalizability of our results. Moreover, this study focuses on two distinct VCC designs but does not exclude the possibility of hybrid forms or combinations. Furthermore, recruiting participants through department heads and managers may have introduced selection bias. Combined with differences in respondents’ professional roles, this may have influenced the perspectives captured and consequently limit the generalizability of the findings. Moreover, subsequent studies should examine how organizational design choices impact cost-effectiveness and operational efficiency, broadening the understanding of how organizational designs of VCCs influence RPM outcomes. Last, a selection bias may be present, as department heads were approached to select eligible individuals to participate using a non-probability sampling method, necessitated by the specific expertise required for meaningful participation. While efforts were made to include participants from diverse professional backgrounds to capture a broad range of perspectives, this limited comparability across groups, as differences in roles may influence perceptions and experiences in ways that are not uniformly comparable.

## Conclusion

This qualitative study examined the organizational designs of three VCCs in Dutch hospitals. We identified two distinct organizational designs: decentralized and centralized VCC designs, revealing their operational, collaborative, and patient-healthcare provider relationships variations. Decentralized VCCs foster strong regional collaboration and personal contact, but face scalability challenges, while centralized VCCs optimize resources at scale but struggle with maintaining care integration and personal relationships. Both organizational designs encounter difficulties in achieving integration across multiple disciplines and maintaining relational continuity of care.

## Supporting information

S1 File A 32-item checklist for reporting qualitative studies (COREQ). (DOCX)

S2 File Interview topic guide. (DOCX)
