# Supplementary material for: The organization of virtual care centers: A qualitative study in Dutch hospitals
Source: PLOS Digit Health. 2026 Jun 26;5(6):e0001479. doi: 10.1371/journal.pdig.0001479 (PMC13308795; doi:10.1371/journal.pdig.0001479)
Supplement: S1 File — (DOCX) [file pdig.0001479.s001.docx]

**S1 File. A 32-item checklist for reporting qualitative studies (COREQ)**

| **Item** | **Description** | **Check** |
| --- | --- | --- |
| **Domain 1: Research team and reflexivity** | | |
| *Personal characteristics* | | |
| 1. Interviewer | Which author/s conducted the interviews and/or observations? | Author 1 |
| 2. Credentials | What were the researcher’s credentials? | Author 1: MSc  Author 2: PhD  Author 3: MA |
| 3. Occupation | What was the occupation at the time of the study? | Author 1: Master's student  Author 2: Assistant professor  Author 3: Head of Virtual Care Center |
| 4. Gender | Was the researcher male or female? | Author 1: Female  Author 2: Male  Author 3: Male |
| 5. Experience and training | What experience or training did the researcher have? | Courses on Qualitative Research Methods at [Information omitted due to review process] and previous experience during thesis writing. |
| *Relationship with participants* | | |
| 6. Relationship established | Was a relationship established prior to study commencement? | No prior relationship was established with the participants before the study commenced. |
| 7. Participant knowledge of the researcher | What did the participants know about the researcher? | Participants were informed about the researchers' institutional affiliations, the study’s objectives, and the nature of their involvement before participation. |
| 8. Researcher characteristics | What characteristics were reported about the researcher? | Researchers obtained an academic background in healthcare (operations) management and digital health which contributed to their interest in the study. |
| **Domain 2: Study design** | | |
| *Theoretical framework* | | |
| 9. Methodological orientation and Theory | What methodological orientation was stated to underpin the study? | Thematic analysis was employed to analyze the qualitative data. |
| *Participant selection* | | |
| 10. Sampling | How were participants selected? | Participants were selected using purposive sampling based on their roles in virtual care centers. |
| 11. Method of approach | How were participants approached? | Participants were approached via email and telephone by their department heads and/or managers. |
| 12. Sample size | How many participants were in the study? | Twelve healthcare professionals participated in the study. |
| 13. Non-participation | How many people refused to participate or dropped out? Reasons? | People did not refuse to participate, but four people were not included as data saturation had already been reached. |
| *Setting* | | |
| 14. Setting of data collection | Where was the data collected? | Data collection took place in both online and face-to-face settings at participants' workplaces. |
| 15. Presence of non-participants | Was anyone else present besides the participants and researchers? | No non-participants were present during the interviews. |
| 16. Description of sample | What are the important characteristics of the sample? | The sample consisted of healthcare professionals working in decentralized and centralized virtual care centers in the Netherlands, including nurses, project managers, and medical specialists. |
| *Data collection* | | |
| 17. Interview guide | Were questions, prompts, guides provided by the authors?  Was it pilot tested? | A semi-structured interview guide was used, based on literature and expert consultation. It was pilot tested before use. |
| 18. Repeat interviews | Were repeat interviews carried out? If yes, how many? | No repeat interviews were conducted. |
| 19. Audio recording | Did the research use audio recording to collect the data? | All interviews were audio-recorded. |
| 20. Field notes | Were field notes made during and/or after the interview or observation? | Field notes were taken after the interviews. |
| 21. Duration | What was the duration of the interviews or observation? | Each interview lasted between 27-42 minutes. |
| 22. Data saturation | Was data saturation discussed? | Data saturation was achieved after 12 interviews, confirmed through thematic consistency. |
| 23. Transcripts returned | Were transcripts returned to participants for comment and/or correction? | Transcripts were returned to participants for validation and minor corrections were provided. |
| **Domain 3: Analysis and findings** | | |
| *Data analysis* | | |
| 24. Number of data coders | How many data coders coded the data? | One researcher coded the data, incorporating interim evaluations and feedback from other researchers. |
| 25. Description of the coding list | Did authors provide a description of the coding list? | One researcher coded the data, incorporating interim evaluations and feedback from other researchers. |
| 26. Derivation of themes | Were themes identified in advance or derived from the data? | Themes were derived from the data; initial codes emerged from the data while incorporating insights from scientific literature used in the interview topic guide. |
| 27. Software | What software, if applicable, was used to manage the data? | ATLAS.ti was used for qualitative data management and coding. |
| 28. Participant checking | Did participants provide feedback on the findings? | Participants were asked to reflect on and validate the study’s findings. Four textual suggestions were made and incorporated. |
| *Reporting* | | |
| 29. Quotations presented | Were participant quotations presented to illustrate the themes/findings? Was each quotation identified? | Direct participant quotations were included in the manuscript to illustrate findings. |
| 30. Data and findings consistent | Was there consistency between the data presented and the findings? | Findings aligned with the data and were systematically analyzed to ensure consistency. |
| 31. Clarity of major themes | Were major themes clearly presented in the findings? | Major themes were presented and discussed in the findings. |
| 32. Clarity of minor themes | Is there a description of diverse cases or discussion of minor themes? | Minor themes and diverse perspectives were included in the discussion to provide a comprehensive analysis. |
