# Supplementary material for: The organization of virtual care centers: A qualitative study in Dutch hospitals
Source: PLOS Digit Health. 2026 Jun 26;5(6):e0001479. doi: 10.1371/journal.pdig.0001479 (PMC13308795; doi:10.1371/journal.pdig.0001479)
Supplement: S2 File — (DOCX) [file pdig.0001479.s002.docx]

**S2. File. Interview topic guide**

| 1. We start with   **The organization of remote patient monitoring in general** | *Main question*  **Could you describe how remote patient monitoring has been organized?**  *Probing questions*   - Can you describe how incoming alerts are typically handled within the remote patient monitoring department? - In what situations are alerts managed independently, without involving a medical or nursing specialist? Could you give examples? - When an alert requires consultation, how does the communication between the monitoring staff and medical/nursing specialists usually take place? - Are there cases where alerts are forwarded directly to medical/nursing specialists? What tends to trigger that immediate handover? - How would you describe the nature of the contact between the monitoring center and the patient?   - What forms does it usually take (e.g., through Luscii messages, phone calls, or video calls)?   - How personal do you think it is? |
| --- | --- |
| 1. We will dive deeper into the   **The organization of remote patient monitoring** | *Main questions*  **What are the differences between remote patient monitoring at your hospital, compared to other hospitals?**  **What are the similarities between remote patient monitoring at your hospital, compared to other hospitals?**  *Probing questions*   - To what extent do you notice advantages of the organizational design applicable to you? - To what extent do you notice disadvantages of the organizational design applicable to you? - To what extent do you think the organizational design influences the quality of remote patient monitoring? - To what extent do you think the organizational design affects the provision of patient-centered care (care from the perspective of the patient)? |
| 1. We will dive deeper into   **Patient-centered care**  **#1 Biopsychosocial perspective** | *Explanation: Biopsychosocial means that patients with chronic illnesses face various challenges: at social, emotional, and physiological levels. Providing care from a biopsychosocial perspective supports understanding and awareness of these aspects beyond the physical 'illness' of the patient.*  *Main question*  **Can you explain how remote patient monitoring influences the biopsychosocial approach in healthcare?**  *Probing questions*   - To what extent does remote patient monitoring influence the social aspects of being chronically ill in a patient? - To what extent does remote patient monitoring influence the emotional aspects of being chronically ill in a patient? - To what extent does remote patient monitoring influence the physiological aspects of being chronically ill in a patient? - How does remote patient monitoring assist medical specialists/nurses in understanding and treating patients with various challenges? |
| 1. We will dive deeper into   **Patient-centered care**  **#2 Patient-as-person** | *Explanation: Person-centered care means that care is provided from the perspective of the patient, with a focus on understanding the patient's perspective within their living situation.*  *Main question*  **Can you explain how remote patient monitoring contributes to person-centered care?**  *Probing questions*   - What are the main benefits of remote patient monitoring regarding person-centered care? - In what ways can remote patient monitoring help understand the living situation and needs of the patient? - To what extent does the organizational design contribute to providing person-centered care from your perspective? - What could healthcare institutions do to ensure that remote patient monitoring contributes to person-centered care? |
| 1. We will dive deeper into   **Patient-centered care**  **#3 Sharing power and responsibility** | *Main questions*  **Can you explain how remote patient monitoring contributes to the perception of control and self-management of the patient?**  **Can you explain how remote patient monitoring contributes to the perception of responsibility of the patient?**  *Probing questions*   - In what ways can remote patient monitoring strengthen patients' perception of control and self-management? - In what ways can remote patient monitoring provide more autonomy over the patient's illness? - Can you explain to what extent remote patient monitoring contributes to the shared decision-making process between the patient and the medical specialist/nurse? - To what extent does the organizational design contribute to the patient's perception of control and self-management? |
| 1. We will dive deeper into   **Patient-centered care**  **#4 Therapeutic alliance** | *Main question*  **Can you explain how remote patient monitoring affects the relationship between the doctor and the patient?**  *Probing questions*   - How does the relationship between the patient and the medical specialist/nurse differ when remote patient monitoring is applied compared to situations where remote patient monitoring is not present? - How can remote patient monitoring help build a relationship between the medical specialist/nurse and the patient? - How can remote patient monitoring contribute to the relationship between the medical specialist/nurse and the patient? - To what extent does the organizational design affect the relationship between the medical specialist/nurse and the patient? |
| 1. We will dive deeper into   **Patient-centered care**  **#5 Doctor-as-person** | *Explanation: Doctor-as-person means that healthcare providers bring their own style and personal characteristics into providing care. This involves self-awareness of emotional responses and creating an emotional connection between the patient and the healthcare provider.*  *Main question*  **Can you explain how remote patient monitoring influences the personal characteristics and style of the medical specialist/nurse?**  *Probing questions*   - How do the style and personal characteristics of the medical specialist/nurse differ when remote patient monitoring is applied, compared to situations where remote patient monitoring is not used? - To what extent does the organizational design affect the style and personal characteristics of the medical specialist/nurse in providing care? |
| 1. Last questions and tips | - Are there any other things you would like to mention about the organizational design of remote patient monitoring? - What tips would you like to give to the organization regarding the organization of remote patient monitoring? |
