# Supplementary material for: The organization of virtual care centers: A qualitative study in Dutch hospitals
Source: PLOS Digit Health. 2026 Jun 26;5(6):e0001479. doi: 10.1371/journal.pdig.0001479 (PMC13308795; doi:10.1371/journal.pdig.0001479)
Supplement: Attachment — Submitted filename: PDIG-D-25-00451 (2)_Rev.pdf [file pdig.0001479.s003.pdf]

PLOS Digital Health

The organization of virtual care centers: A qualitative study in Dutch hospitals

--Manuscript Draft--

**Manuscript Number:**	PDIG-D-25-00451
**Article Type:**	Research Article
**Full Title:**	The organization of virtual care centers: A qualitative study in Dutch hospitals
**Short Title:**	Organizational designs of virtual care centers
**Corresponding Author:**	Vincent J.T. Peters, Dr<br>Tilburg University<br>Tilburg, NETHERLANDS, KINGDOM OF THE
**Order of Authors:**	Mirte A.M. Huynen
	Vincent J.T. Peters, Dr
	Martinus A. van Geffen
**Keywords:**	Virtual care center; remote patient monitoring; virtual care
**Abstract:**	Virtual care centers (VCCs) facilitate remote patient monitoring, which has been shown to reduce secondary care utilization among patients, improve efficiency for healthcare providers, and decrease overall healthcare costs. Although VCCs are increasingly being employed, the way they are organized varies. Existing literature often conceptualizes VCCs as independent hospital wards, yet alternative organizational designs are emerging. However, there is a lack of empirical research examining how these designs differ and function. This study aims to explore and characterize the organizational designs of VCCs to provide a clearer understanding of their structure and implications. This qualitative, exploratory study, examined three VCCs in the Netherlands, selected through purposive sampling based on the volume of patients receiving virtual care and the range of care pathways offered. In April and May 2024, semi-structured interviews were conducted with twelve healthcare professionals. Thematic analysis was used to analyze the interview data. Two distinct organizational designs for VCCs were identified: a decentralized and centralized design. Themes and subthemes were derived to explore the different characteristics of the two organizational designs. Decentralized VCCs promote strong regional collaboration, fostering closer professional relationships and communication between healthcare providers and patients. However, their scalability is limited due to resource constraints and localized operational frameworks. In contrast, centralized VCCs optimize resource allocation, enabling broader patient coverage, and extended accessibility yet face challenges in maintaining care integration, trust among healthcare providers, and relational continuity with patients. Both designs exhibit difficulties in achieving multidisciplinary care integration, which is crucial for effectively supporting patients with complex and chronic conditions. Future research should include patient perspectives, and evaluate how VCCs accommodate varying care pathways to further inform evidence based organizational design improvements.
**Additional Information:**	
**Question**	**Response**
**Government Employee**<br><br>Are you or any of the contributing authors an employee of the United States government?<br><br>Manuscripts authored by one or more US Government employees are not	No - No authors are employees of the U.S. government.

Powered by Editorial Manager® and ProduXion Manager® from Aries Systems Corporation

copyrighted, but are licensed under a CC0 Public Domain Dedication, which allows unlimited distribution and reuse of the article for any lawful purpose. This is a legal requirement for US Government employees.<br><br>This will be typeset if the manuscript is accepted for publication.	
**Financial Disclosure**<br><br>Enter a financial disclosure statement that describes the sources of funding for the work included in this submission. Review the submission guidelines for detailed requirements. View published research articles from *PLOS Digital Health* for specific examples.<br><br>This statement is required for submission and **will appear in the published article** if the submission is accepted. Please make sure it is accurate.<br><br>**Funded studies**<br>Enter a statement with the following details:<br>• Initials of the authors who received each award<br>• Grant numbers awarded to each author<br>• The full name of each funder<br>• URL of each funder website<br>• Did the sponsors or funders play any role in the study design, data collection and analysis, decision to publish, or preparation of the manuscript?<br><br>Did you receive funding for this work?	The author(s) received no specific funding for this work.
**Competing Interests**<br><br>On behalf of all authors, disclose any competing interests that could be perceived to bias this work.<br><br>This statement will be typeset if the manuscript is accepted for publication.	The authors have declared that no competing interests exist.

Review the instructions link below and PLOS Digital Health's competing interests policy to determine what information must be disclosed at submission.	
**Data Availability**<br><br>Provide a **Data Availability Statement** in the box below. This statement should detail where the data used in this submission can be accessed. This statement will be typeset if the manuscript is accepted for publication.<br><br>Before publication, authors are required to make all data underlying their findings fully available, without restriction. Review our PLOS Data Policy page for detailed information on this policy. Instructions for writing your Data Availability statement can be accessed via the Instructions link below.	The data that support the findings of this study are publicly available from Tilburg University's data repository, TiU Dataverse. This repository has been certified with the CoreTrustSeal. You can access the repository once the paper has been accepted.

Powered by Editorial Manager® and ProduXion Manager® from Aries Systems Corporation

Manuscript

Click here to access/download;Manuscript;20250710 Manuscript Virtual care centers.docx

The organization of virtual care centers:

A qualitative study in Dutch hospitals

Mirte A.M. Huynen[a,b], Vincent J.T. Peters[a*], Martinus A. van Geffen[b]

[a] Department of Information Systems and Operations Management, Tilburg University, Tilburg, the Netherlands

[b] Department of Home Monitoring, Jeroen Bosch Hospital, ‘s-Hertogenbosch, the Netherlands

* Corresponding author

E-mail: v.j.t.peters@tilburguniversity.edu (VP)

ORCID ID: 0000-0002-9777-7777

Abstract

Virtual care centers (VCCs) facilitate remote patient monitoring, which has been shown to reduce secondary care utilization among patients, improve efficiency for healthcare providers, and decrease overall healthcare costs. Although VCCs are increasingly being employed, the way they are organized varies. Existing literature often conceptualizes VCCs as independent hospital wards, yet alternative organizational designs are emerging. However, there is a lack of empirical research examining how these designs differ and function. This study aims to explore and characterize the organizational designs of VCCs to provide a clearer understanding of their structure and implications. This qualitative, exploratory study, examined three VCCs in the Netherlands, selected through purposive sampling based on the volume of patients receiving virtual care and the range of care pathways offered. In April and May 2024, semi-structured interviews were conducted with twelve healthcare professionals. Thematic analysis was used to analyze the interview data. Two distinct organizational designs for VCCs were identified: a decentralized and centralized design. Themes and subthemes were derived to explore the different characteristics of the two organizational designs. Decentralized VCCs promote strong regional collaboration, fostering closer professional relationships and communication between healthcare providers and patients. However, their scalability is limited due to resource constraints and localized operational frameworks. In contrast, centralized VCCs optimize resource allocation, enabling broader patient coverage, and extended accessibility yet face challenges in maintaining care integration, trust among healthcare providers, and relational continuity with patients. Both designs exhibit difficulties in achieving multidisciplinary care integration, which is crucial for effectively supporting patients with complex and chronic conditions. Future research should include patient perspectives, and evaluate how VCCs accommodate varying care pathways to further inform evidence based organizational design improvements.

Author summary

VCCs allow patients to be remotely monitored in their home environments using digital technologies. Although VCCs have become increasingly prevalent in healthcare systems, particularly in response to the COVID-19 pandemic, there is an ongoing debate about the organizational design of a VCC. Two distinct deigns have emerged: decentralized VCCs, which are embedded within individual hospitals, and centralized VCCs, which operate across multiple hospitals on a national scale. Despite the growing implementation of both designs, empirical research on their organizational differences and practical implications remains limited. This study addresses that gap by interviewing twelve healthcare professionals across three VCCs in the Netherlands. Understanding these differences can support evidence-informed decisions on organizing virtual care delivery more effectively.

Background

Virtual care centers (VCCs) facilitate remote patient monitoring in hospitals as a response to growing challenges, including increasing care demand, decreasing healthcare provider availability, an aging population, and the rising prevalence of chronic diseases [1-5]. VCCs facilitate a mix of digital and physical care, through remote patient monitoring. Remote patient monitoring has been defined as the supervision and guidance of patients through the remote monitoring of vital indicators and symptoms for a particular disease [6]. VCCs provide monitoring for two distinct patient groups: individuals who are discharged early after surgery, for example a breast reconstruction surgery [7] and those with chronic diseases, such as Chronic Obstructive Pulmonary Disease (COPD) and diabetes [8]. Patients enter their medical data related to their health condition in an application using a (wireless) device or sensors automatically transmit the data. When their values exceed a predefined threshold, the application issues a notification prompting the repetition of a measurement or introduces additional query addressing specific symptoms. Nurses staffing the VCC monitor these alerts, offering support through digital or phone appointments, thereby enabling remote care delivery.

Previous research has examined benefits of remote patient monitoring, including time and cost savings [9], reduced use of secondary healthcare for patients with chronic diseases [10-13], and a decrease in hospital readmissions for patients with chronic conditions [8, 13-15]. The effectiveness of remote patient monitoring has been examined to depend on the initial capital investment and the specific healthcare context [15]. Despite these findings, existing literature conceptualizes remote patient monitoring as a technological intervention, with limited attention to the organizational design through which it is deployed, such as VCCs.

The current literature identifies the organizational design of a VCC as an independent ward within the hospital, that serves as the primary interface within a hospital and has to distinguish between critical and non-critical cases [16-18]. Although VCCs are becoming common practices in hospitals, the organizational design is primarily shaped by clinical and administrative judgement, accelerated by the COVID-19 pandemic, with limited reliance on evidence-based approaches. Consequently, existing literature lacks an exploration of organizational designs of VCCs and their respective outcomes. Therefore, the aim of this study is to examine organizational designs of VCCs in Dutch hospitals.

Methods

Study design

A qualitative study design was adopted for this study, using semi-structured interviews with (healthcare) professionals employed in VCCs in the Netherlands. This exploratory, qualitative study has been conducted following the consolidated criteria for reporting qualitative research (COREQ) (S1 File) [19]. The ethics review board of [Omitted for review purposes] evaluated and approved the study design.

Recruitment and sampling

Using purposive sampling, ten VCCs in the Netherlands employing remote patient monitoring were identified [20]. Three VCCs were included in our study (A, B, and C) based on their suitability, as determined by the numbers of patients receiving digital care and the included care pathways, which refer to structured plans that encompass both clinical procedures and supportive services in managing a particular type of patient (Table 1) [21].

Table 1. Characteristics of Virtual Care Centers in the Netherlands (2025).

VCC	Patients receiving virtual care (n)	Healthcare providers employed in VCC (n)	Care pathways in VCC (n)
A	6,000	30	42
B	600	13	24
C	23,000	50	27

The number of care pathways was used as a proxy measure to assess the extent of remote patient monitoring implemented within each VCC, with a greater number of care pathways indicating broader applicability of such monitoring across diverse patient groups. VCC department heads and managers were approached via email or telephone to invite eligible colleagues to participate in the study. The inclusion criteria specified that participants must have engaged in the development or provision of remote patient monitoring by means of a VCC and should be proficient in speaking and understanding the Dutch language. A diverse

group of sixteen participants was initially approached, with twelve ultimately included in the study, encompassing a wide range of professional expertise (Table 2). Data saturation was achieved when repetition in participant's answers occurred; this indicated that the range of relevant perspectives had been captured sufficiently and more interviews were unlikely to contribute to new understanding of the research questions [22]. Written informed consent was obtained from all participants prior to their involvement in the study.

Table 2. Participant characteristics.

Participant	VCC	Profession	Age (years)	Gender
1	A	Nurse	31	Female
2	A	Nurse and Project Manager	27	Female
3	A	Nurse	33	Female
4	A	Professional Nursing Manager	42	Female
12	A	Medical Project Manager and Cardiologist	46	Female
5	B	Project Manager	54	Female
6	B	Nurse	39	Female
7	B	Scientific Researcher and Nursing Manager	31	Male
8	C	Project Manager	43	Female
9	C	Project Manager	39	Female
10	C	Head of VCC	30	Male
11	C	Nurse	46	Female

Data collection

Semi-structured interviews were conducted in April and May 2024 in both face-to-face and online settings, lasting approximately 30 minutes each. An interview topic guide based on scientific literature [23-25] was used for the interviews (S2 File). Questions were categorized into two sections: the first focus on the internal functioning and organizational design of the VCC, while the second section explored its integration within each respective hospital. The interviews were audio-recorded and transcribed. In addition, a member check with all participants was performed to verify the accuracy and credibility of the transcripts, allowing participants to provide feedback [26]. Four suggestions regarding the textual content were given and incorporated. Additionally, secondary data, including brochures and folders, was obtained from the department heads and managers, regarding the organizational design of the respective VCC.

Data analysis

The interview transcripts were analysed using ATLAS.ti. We used the thematic analysis approach by Braun and Clarke [27] to assign codes to relevant data segments and categorized them into themes. In accordance with an inductive reasoning approach, initial codes were generated from the data [28, 29]. This initial coding process started with an open coding process, followed by identifying key concepts and themes within the data. Subsequently, axial coding was employed to establish connections between these initial codes. This coding process led to the identification of two distinct organizational designs of VCCs: a decentralized and centralized design. We then identified themes that captured the characteristics and implications of these organizational designs. The first theme captured the operational framework of VCCs, while the other themes addressed their integration within the hospital(s), including topics such as collaboration between the healthcare providers and the patient-healthcare provider relationship. Subthemes were further refined to reflect their specific implications, illustrated with quotes from the interview data (Table 3).

Table 3. Example of a theme resulting from our thematic analysis.

**Theme**	**Subtheme**	**Illustrative quote Decentralized design**	**Illustrative quote Centralized design**
Collaboration between healthcare providers	Personal contact between colleagues	*"The biggest difference is that you have shorter communication lines when monitoring within a hospital. You see the medical specialists walking around and you work with them."* (Participant 2 – VCC A)	*"Well, I think that those lines of communication become much longer because you do not know the colleagues from the other hospital personally. Collaborating becomes more challenging."* (Participant 4 – VCC A)
	Trust in colleagues	*"I think there is more mutual trust, because you simply work together intensively within the same hospital."* (Participant 2 – VCC A);<br><br>*"Trust also grows more quickly between us."* (Participant 4 – VCC A)	*"You need trust in the medical protocol and trust in the nurses who are monitoring for you, and if they are your own nurses, then that is somewhat easier. But if it is a nurse from another hospital, then there is a challenge in the trust model."* (Participant 10 – VCC C)

	Integrated care	*"A regional [decentralized] VCC makes it easier to work with local general practitioners, physiotherapists, social workers, and everything else that comes with it. Offering one package is less easy to organize at a national [centralized] VCC, because it covers a larger area."* (Participant 9 – VCC C)	*"A national organization forces you to negotiate with professional associations, such as the Dutch Association of Cardiologists, for example. Then there is immediately a national protocol in place."* (Participant 12 – VCC A)

Results

This study identified two distinct organizational VCC designs: decentralized and centralized designs. A decentralized VCC, as observed in VCC A and B, operates as an independent unit within a hospital, where nurses (who are supervised by a project manager) oversee daily patient alerts, addressing the needs of their 'own' patients. In contrast, a centralized VCC, exemplified by VCC C, serves patients across multiple hospitals and is staffed by nurses and medical specialists delegated from the involved hospitals, who collaboratively manage incoming alerts.

These two organizational VCC designs differ in three themes: 1) operational framework, 2) collaboration between healthcare providers, and 3) patient-healthcare provider relationships. Corresponding subthemes have also been identified (Table 4).

Table 4. Themes and subthemes across decentralized and centralized VCC designs.

Theme	Subtheme	Decentralized design	Centralized design
Operational framework	Monitoring and alerts	Patients are monitored by nurses at their respective hospital, nurses can consult with the medical specialist of the patient if necessary. Expansion in the number of patients is limited by the capacity of the individual hospital.	A front-office for non-medical alerts; medical alerts are managed by nurses and delegated medical specialists from different hospitals. Designed for a larger patient population of secondary care patients.

	Standard operating procedures	Variation in standard operating procedures across hospitals. Programs for the included care pathways are collaboratively developed within the hospital and reflect local policies. Operating hours and services limited by in-house staffing and resources.	National uniformity in standard operating procedures and programs for the included care pathways, yet challenges arise in aligning the policies across the included hospitals. Increased accessibility for patients due to extended opening hours and optimized resource utilization.
	System integration and file accessibility	Focus on local integration of systems within each hospital or healthcare unit. EHRs accessibility is available within single hospitals, but does not extend to regional healthcare providers (e.g. GPs)	Required comprehensive integration of systems at a national level to ensure seamless data exchange. Uniform accessibility across all hospitals through shared digital platforms.
Collaboration between healthcare providers	Personal contact between colleagues	Direct interaction and communication between colleagues from the VCC and the respective hospital.	Limited personal interaction with the VCC and hospitals, as collaboration often occurs remotely and VCC operates more independently.
	Trust in colleagues	High levels of trust in colleagues of the VCC. Lower levels of trust in VCC colleagues from other hospitals.	Lower levels to trust VCC colleagues from other hospitals. Trust is fostered through formalized agreements across different hospitals.
	Integrated care delivery	Focus on network care, collaboration with GPs, and regional healthcare providers.	National disciplinary collaboration: disciplines (e.g. cardiology) sharing expertise, practices, and research.
Patient-healthcare provider relationships	Personal contact between patient and healthcare provider	High levels of personal contact between the healthcare providers and patients; addressing emotional, social, and physiological challenges of patients.	Moderate to low levels of personal contact between healthcare provider and patient due to larger numbers of patients. Expected increased accessibility due to quicker patient assistance.
	Trust between patient and healthcare provider	Patients value the trust established with their familiar local providers and employees of the VCC.	Trust in the relationship may be established through standardized care protocols.
	Sharing power and responsibility	Care delivery through VCCs fosters sharing power and responsibility; however, it introduces a potential bias for patient inclusion, as not all patients may be equally presented.	
	Continuity of care	Potentially enhancing continuity of care by ensuring seamless transitions across various healthcare providers within the same region.	More challenging to achieve continuity of care due to less direct contact between the patient, healthcare provider, and the VCC.

Theme 1. Operational framework

Monitoring and alerts

The remote patient monitoring process and handling of alerts varies between a decentralized and centralized VCC. In decentralized VCCs, nurses within each individual hospital manage incoming alerts based on the care pathways developed within the respective hospital, tailored

to local practices and patient needs. Alerts are distributed on a daily basis among the nurses based on the respective care pathway programs or urgency criteria. Nurses manage the alerts independently and have the freedom to consult with the patient's treating medical specialist when necessary: *"We receive the alert on the worklist, which we can then forward to the specialist. This happens about 20% of the time, and for some higher priority programs, there is a daily designated timeslot. If there is something that needs to be discussed, we often just make a phone call to the (nursing) specialist, and the alert is resolved quickly."* (Participant 1 – VCC A). In contrast, centralized VCCs employ a more governed and collaborative approach. A front-office filters out non-medical alerts, reducing the workload for clinical staff and ensuring that only relevant medical alerts are escalated. As explained by one participant: *"If the patient is unable to install an app, you do not need nursing expertise. Someone who is trained in how to install apps can handle it as well."* (Participant 9). Once filtered, monitoring occurs centrally, following standardized procedures designed to ensure consistency across different hospitals and regions. Delegated nurses and specialists from various hospitals manage the alerts within the VCC and tasks can be delegated based on specialization, potentially enhancing efficiency in managing digital alerts: *"If a nurse is employed more efficiently, for example responsible for one care pathway, she can handle alerts from that pathway more quickly and effectively. So now you might be calculating that one nurse can monitor 500 patients. But maybe you will even grow to a point where one nurse can monitor 2,000 patients. So I think there could be a lot of opportunities and differences there — provided that the technology keeps up as well."* (Participant 9 – VCC C). Centralized VCCs can demonstrate scalability advantages in terms of managing a larger patient base in absolute numbers. By optimizing resource allocation and streamlining processes, centralized VCCs can efficiently accommodate more patients: *"Once you have streamlined the processes, scalability is definitely your advantage."* (Participant 9 – VCC C).

Standard operating procedures

The care protocols and programs used by hospitals to manage different care pathways are considered as part of the standard operating procedures (SOP) of VCCs. A tension arises between digital care delivery based on programs and protocols (standardization) versus care tailored to the patient's needs and requirements (customization). One nurse emphasized: *"In principle, it [digital care] is very much focused on programs. So, it is quite standardized in*

its setup." (Participant 3 – VCC B). For a decentralized VCC design, instances were mentioned wherein remote care included tailored care interventions that adhered to the specific needs and preferences of individual patients. As one participant emphasized: *"There is sometimes room to make adjustments. For example, a program for high blood pressure, then people have to measure four times a day at the start. You can imagine if you are 35 years and have a job, that is not easy. Then we can see if we can solve that differently."* (Participant 4 – VCC B). Decentralized SOP reflect local policies, providing flexibility yet introducing variability, while centralized VCCs aim to enhance efficiency through SOP: *"Setting up the same processes across the entire country is much more efficient. You can expand beyond just those small regions and achieve efficiency gain with that."* (Participant 9 – VCC C). Moreover, centralized VCCs can enhance the nurses' ability and promote greater efficiency in managing alerts. For example, if nurses assist a greater number of patients of similar care pathways, their clinical experience and expertise related to the program will increase, potentially allowing for faster responsiveness to the patient's needs and preferences. As exemplified by one of the participants: *"A nurse at a local level might have to handle three or four programs. On a larger scale, it would be just one or two, allowing you to delve much deeper into one. You will be quicker, so more efficient. You can also provide better advice."* (Participant 10 – VCC C). However, while centralized VCCs may promote consistency through SOP, it faces challenges in regional alignment: *"If you ultimately create one protocol, you still have seven different procedures [for each region]. It requires a lot of work to align them."* (Participant 11 – VCC C). Moreover, geographical differences further challenge standardization in centralized VCCs: *"You hear differences in the Netherlands, because in Groningen, when someone is 'not feeling well', they are lying half-dead on the coach. While in Amsterdam, 'not feeling well' means immediately going the hospital. It varies per region how patients react."* (Participant 8 – VCC C). Additionally, the standard operating procedures of decentralized VCCs are constrained by limited operating hours due to resource limitations, reducing accessibility for patients. Conversely, centralized VCCs offer extended opening hours, enhancing accessibility and ensuring continuity of care. As one participant emphasized: *"Additionally, because you have so many patients, a lot of alerts will come in and you will have to employ more nurses. You can then choose to implement a 7-day week or a 24-hour service model because there are enough alerts coming in to keep someone working. With 2,000 patients, you cannot really justify scheduling an evening or night shift"* (Participant 10 – VCC C).

System integration and file accessibility

Decentralized VCCs focus on monitoring devices within their own hospital systems. In contrast, centralized VCCs face a more complex task, requiring seamless interoperability across multiple hospitals and regions. This includes aligning the EHR, monitoring devices, and other digital (communication) tools to enable efficient communication and data sharing across hospitals. As explained by one of the participants: "*They are currently working with different apps, different communication modules, and different EHRs. All of that is located in different places. It is a complex challenge to bring that together*." (Participant 10 – VCC C).

In traditional care, access to EHRs is typically confined to each respective hospital, limiting the ability to share patient information with external providers. Similarly, in decentralized VCCs, EHR accessibility remains local and often does not extend to regional care providers like general practitioners (GPs), creating barriers to seamless integration of care. However, centralized VCCs rely heavily on data sharing across hospitals to ensure uniform access to patient information. Participants from all hospitals emphasized the importance of EHR accessibility as a critical component for effective remote patient monitoring, regardless of the organizational design of the VCC: *"As long as we do not have all information about the patient's health status, including information regarding mental health and how the patient wants to be coached, it becomes very difficult to provide personalized care."* (Participant 12 - VCC A).

Theme 2. Collaboration between healthcare providers

Personal contact between colleagues

In decentralized VCCs, personal contact between colleagues from the VCC and the hospital is a defining feature. Direct interaction among colleagues within the hospital fosters informal communication and collaborative problem-solving. Multiple participants highlighted this advantage: *"The biggest difference is that you have shorter communication lines when monitoring within a hospital. You see the medical specialists walking around and you work with them."* (Participant 2 – VCC A) and *"I think it is a big difference that we personally know everyone we work with. Each program is built and evaluated personally by us, with*

someone from the specialty." (Participant 4 – VCC A). Conversely, centralized VCCs result in greater geographical distance between colleagues, as they operate across multiple hospitals and regions. A participant from a decentralized VCC expressed concerns that communication and teamwork could be more challenging in this design: *"Well I think that those lines of communication become much longer because you do not know the colleagues from the other hospital personally. Collaborating becomes more challenging."* (Participant 4 – VCC A).

Trust in colleagues

Trust emerged as an important feature for decentralized VCCs, where colleagues operate within the same hospital. As explained by two participants: *"I think there is more mutual trust, because you simply work together intensively within the same hospital."* (Participant 2 – VCC A) and *"Trust also grows more quickly between us [colleagues]."* (Participant 4 – VCC A). In contrast, centralized VCCs may encounter challenges in establishing trust, as medical specialists rely on nurses affiliated with other hospitals, highlighting the importance of inter-organizational trust. As one participant explained: *"You need trust in the medical protocol and trust in the nurses who are monitoring for you, and if they are your own nurses, then that is somewhat easier. But if it is a nurse from another hospital, then there is a challenge in the trust model."* (Participant 10 –VCC C). Similarly, one participant emphasized: *"What you have within your own organization, within your own capabilities, has to be handed over to another organization. And then you have to trust that it goes well.*" (Participant 9 – VCC C). The reliance on other hospitals necessitates the creation of trust through protocols to realize effective collaboration.

Integrated care delivery

The extent to which integrated care is delivered varies across the two VCC designs. Decentralized VCCs have been examined to facilitate regional network care: collaborating with GPs and other local primary healthcare providers to address specific regional needs. As one participant noted: *"For example, we have a palliative program for end-stage heart failure, requiring collaboration with primary care, home care, and cardiology. [...] You need to know exactly with GP and district nurse to contact. The local knowledge is crucial, making the local approach more effective."* (Participant 12 – VCC A). This advantage has also been

recognized by a participant of a centralized VCC design: *"A regional [decentralized] VCC makes it easier to work with local GPs, physiotherapists, social workers, and everything else that comes with it. Offering one package is less easy to organize at a national [centralized] VCC, because it covers a larger area."* (Participant 9 – VCC C). Moreover, regional primary healthcare providers play a crucial role in carrying out essential tasks: "*Sometimes the patient needs more morphine, because s/he is short of breath. That falls under the GP's responsibility. Similarly, if the patient feels very anxious and needs reassurance, it is the district nurse who should visit."* (Participant 12 – VCC A). However, this does not imply that centralized VCCs are incapable of achieving similar outcomes compared to decentralized VCCs. As one participant emphasized, successful integration of the technical system is key: *"While regional network care may be easier to implement for a decentralized design, a centralized design can also succeed if the technical system can support it [regional network care]."* (Participant 9 – VCC C). Centralized VCCs provide greater opportunities for promoting integrated care across disciplines, allowing for national collaboration within the same disciplines. As exemplified by the following participant: *"A national [centralized] organization forces you to negotiate with professional associations, such as the Dutch Association of Cardiologists, for example. Then there is immediately a national protocol in place."* (Participant 12 – VCC A). Despite the distinct advantages for decentralized and centralized VCCs in delivering integrated care, realizing integration across multiple disciplines remains a challenge for both VCC designs. As exemplified by one of the participants: *"The specialist responsible for the care pathway, for example the cariologist, knows that the patient is in monitoring, However, if the patient contacts the surgery department the next day, the surgeon has no idea that the patient is included in monitoring. This lack of awareness is the biggest risk for integrated care."* (Participant 6 – VCC B). Moreover, another participant highlighted the lack of communication and integration among different care pathways: *"It is not really multidisciplinary. For example, a patient with COPD and diabetes said that the care providers do not communicate at all. We should build in that multiple programs can be combined in the app."* (Participant 11 – VCC C).

Theme 3. Patient-healthcare provider relationships

Personal contact between patient and healthcare provider

In both centralized and decentralized VCC designs, the patient-provider relationship changes compared to traditional care provision. Participants noted a reduction in direct personal contact for both VCC designs between the treating medical specialist and patient, which aligns with the objective of hybrid care provision to reduce the burden on healthcare providers: "*If, as a patient, you can trust such a virtual care center, then I can very well imagine that the distance to the treating physician or the need for the treating physician will decrease. That is also the whole goal. So, something will change in that relationship."* (Participant 9 – VCC C). Interestingly, participants mentioned that reduced direct personal contact actually increases accessibility of care: *"Many patients initially are afraid for losing contact with their doctor. But eventually, they realize that through the app, they can actually have quicker contact when needed."* (Participant 11 – VCC C). Moreover, another participant emphasized the enhanced accessibility of care: *"Research has examined that telemonitoring makes the contact with the healthcare provider more accessible. This leads to a collaboration between the healthcare providers and the patient."* (Participant 7 – VCC B). Decentralized VCCs seem to be effective in maintaining personal contact between patients and their healthcare providers. Close geographical proximity and familiarity allow patients to feel supported and heard: *"The contact, from what I hear from the patients and experience myself, is very accessible. They generally call us easily and that makes it very personal."* (Participant 4 – VCC A). Moreover, decentralized VCCs have demonstrated to effectively address personal interactions, managing the patient's emotional and social needs: *"Indeed, socially, how is work going or do you feel anxious and down about your illness. We already include those questions in certain programs."* (Participant 2 – VCC A). Centralized VCCs are at risk of reducing the frequency and depth of personal interactions as a result of their scalability and the inclusion of a greater absolute number of patients. While efficiency gains may be achieved in centralized VCCs, this often comes at the expense of a personal relationship between patients and healthcare providers. As one participant from a decentralized design emphasized: *"I also think that the more hospitals you monitor, the more patients you have, the less personal the contact becomes."* (Participant 2 – VCC A). This shift has also been reflected in the centralized VCC: *"Now you would have one nurse for 500 patients, and we could maybe grow to one nurse for 2000 patients. [...] You do not have your own patients anymore, that is the biggest difference."* (Participant 9 – VCC C) and "*I think that interactions between the patient and healthcare provider are more personal for a decentralized VCC, especially when monitoring fewer patients, compared to a centralized VCC. However, within a centralized VCC, we ensure that nurses have expertise and*

experience with specific types of alerts, allowing patients to receive quicker assistance than in a decentralized design." (Participant 10 – VCC C). The potential advantage of providing quicker assistance by a centralized design may be of greater importance than establishing a patient-provider relationship: *"I think that it is more important for the patient to be helped quickly, than that s/he has to wait for a nurse with whom s/he has built a connection with. But that is an assumption.*" (Participant 10 – VCC 10). Furthermore, some participants noted that reduced levels of direct personal contact are not necessarily harmful, as it may allow healthcare providers to focus on their own expertise: *"I do think that the nursing specialist has less conversations about how things are going [chit-chat], because that is often already identified earlier. Ultimately, they might have less contact. But precisely because of that, they can really focus on their expertise."* (Participant 2 – VCC A). Moreover, one of the participants emphasized that higher levels of direct personal contact may lead to reduced appropriate care: *"The downside of great accessibility in contact, is being too kind to the patient. You should ask yourself, is this really effective care? This is something that still requires further research.*" (Participant 7 – VCC B).

Trust between patient and healthcare provider

Patients in decentralized VCCs often rely on trust established among care providers within the hospital: *"There are short lines of communication with the (nursing) specialist and also with the patient. This increases the accessibility for the patient, familiarity, and the mutual trust."* (Participant 4 – VCC A). In centralized VCCs, remote care delivery may feel impersonal to some, reducing trust in the therapeutic alliance: *"It can feel very impersonal for the patient if you spread it [care provision] over a very large area. Then I can completely understand that the patient loses the connection or trust in it."* (Participant 2 – VCC A). One medical specialist emphasized the trust patients place in remote monitoring, even when the involvement of the specialist is not directly visible: *"As the VCC is currently decentralized, the relationship between the patient and specialist only becomes stronger. The patient has complete trust in the monitoring, believing that I have looked into it, even when that was not the case, and it was actually handled by the VCC."* (Participant 12 – VCC A). For centralized VCCs, one of the participants emphasized the challenge of building trust when there is no personal relationship between the patient and the nurse: *"I think that if those connections are much more distant, because you do not know each other personally, it becomes difficult.*

Trust, which is still essential to achieve the desired outcome, becomes harder to establish." (Participant 4 – VCC A).

Sharing power and responsibility

For both decentralized and centralized VCCs, participants highlighted that remote patient care delivery through VCCs contributes to self-management, the perception of control, and patient responsibility: *"Yes, I think through remote monitoring, they get to know their health condition better. For example, if a patient has had a barbecue and a few beers and ate whatever he wanted, then his weight will increase the next day. In that sense, they learn from what they do and the consequences of it."* (Participant 4 – VCC A). This increased sense of autonomy is complemented by healthcare providers having access to accurate, real-time health data rather than relying on the patients' subjective reports: *"The doctors, also have much more reliable values, because the patient measures at home, potentially adjusting treatment policies accordingly."* (Participant 4 – VCC A). It was consistently emphasized that participation in remote monitoring programs necessitates patients to exhibit some level of self-management and responsibility. Currently, participation in remote monitoring is voluntary. Some patients are not suitable for remote monitoring due to, for example, specific medical conditions, limited digital literacy, age-related factors, or language barriers: *"Not all patients will be eligible for remote monitoring, so you assess what level of responsibility the patient can handle and that varies."* (Participant 7 – VCC B).

Continuity of care

In decentralized VCCs, continuity of care is expected to be more easily achieved by keeping handover of care within the regional network, ensuring that patients receive care from the same team of healthcare providers who are familiar with the patient's medical history and needs. This regional approach promotes strong personal engagement and trust between patients and providers. Conversely, centralized VCCs might face challenges due to the larger scale of operations and reduced direct interaction between patients and their treating healthcare providers. The medical specialists and nurses are delegated from multiple hospitals, which makes it more challenging to preserve the personal relationships and therapeutic alliances that are essential for ensuring continuity of care. As emphasized by one of the participants: *"So it could be that a patient is being treated by a cardiologist in hospital*

A, but enrolled in monitoring through a centralized VCC, with their measurements being assessed by a cardiologist from hospital B. Those two cardiologists are thus not the same person, which is a disadvantage." (Participant 2 – VCC A).

Discussion

Operational implications of VCC design

This study shows the operational implications of two distinct organizational VCC designs and highlights a fundamental tension between localized, hospital-specific operations in decentralized VCCs and standardized processes that regulate centralized VCC designs. In decentralized VCCs, the programs for the included care pathways are collaboratively developed and evaluated within the hospital, reflecting regional standards of care and policies. In contrast, centralized VCCs seem to leverage (national) standardized protocols to achieve scalability, optimize resources, and enable the management of a broader patient base. However, centralized VCCs potentially face challenges in maintaining alignment with diverse regional policies, as integrating various healthcare standards into a cohesive framework remains complex. Although this centralized design potentially improves efficiency, this study does not empirically confirm such improvements. Both organizational VCC designs are characterized by a reliance on program-based care pathways. This reliance may be inherited from traditional care models, where protocol-based care guide care delivery [30], reinforcing a siloed approach in which each discipline operates independently. While this facilitates consistency and can improve efficiency, it may constrain the ability to provide patient-centered care, limiting the flexibility to address individual patient needs [30, 31]. This observation shifts the discussion beyond care delivery by VCCs, suggesting that challenges to personalized care may stem from the foundational design of program-based care pathways rather than the organizational VCC design itself.

Collaboration between healthcare providers

The findings of this study highlight variation in collaborating between healthcare providers within decentralized and centralized VCC designs. Decentralized VCCs foster intense collaboration characterized by frequent personal contact and direct communication among

healthcare providers. These attributes strengthen interpersonal trust and cohesion, as staff within the hospital and VCC are familiar with one another's work processes. In contrast, centralized VCCs introduce a more complex collaborative dynamic, where geographical proximity between healthcare providers may hinder the development of close professional relationships. Medical specialists and nurses from distinct hospitals may experience challenges in building trust and fostering effective communication. Additionally, centralized VCCs require not only the establishment of trust and effective communication between organizations but also formalized inter-organizational governance structures [32]. Next to relational governance, these inter-organizational structures also include contractual mechanisms [33] to ensure that various arrangements, such as finances and data management [34], are properly managed and safeguard the involved parties against potential opportunistic behaviors [35].

The strong relational foundation in decentralized VCCs may support more effective integration and collaboration with regional healthcare providers. However, decentralized VCCs may face limitations in regional expansion. As the patient population increases, the personal contact that characterizes decentralized designs may become unsustainable. This prompts a discussion concerning the scale at which a decentralized VCC reaches the threshold where it transitions into a centralized VCC. Identifying this "cut-off" point is essential for ensuring that effective collaboration and integration are preserved during scaling-up efforts. Future research should dive into this 'cut-off' point.

A broader discussion arises concerning the implications of VCCs on integrated care. While decentralized VCCs support integrated care through expanded regional network care, centralized VCCs potentially promote integrated care through intradisciplinary collaboration on a larger, national scale. However, the findings indicate that both designs face challenges in achieving integration of multidisciplinary care. Fragmented, poorly coordinated care provision leading to inefficiencies, is particularly prevalent among patients with chronic conditions, who often require services from multiple specialists and providers [36]. As patients with chronic diseases represent one of the primary focus areas for remote patient monitoring by VCCs, the absence of multidisciplinary collaboration in this context underscores an important gap that requires further investigation.

Patient-healthcare provider relationships

Our findings accentuate the difference in patient-provider relationships in decentralized and centralized VCC designs. Decentralized VCCs have been examined to facilitate higher levels of personal contact and trust between patient and provider, attributes that have been examined to be foundational to a robust therapeutic alliance and affect patient satisfaction [37, 38]. In contrast, centralized VCCs may reduce opportunities for direct personal interaction, potentially weakening this alliance. While centralized designs have been hailed for their ability to increase the pace of assistance, it is questionable whether this design actually enables quicker assistance. Furthermore, if quicker assistance is indeed achieved, it is important to consider whether this advantage outweighs the value of maintaining a personal patient-provider relationship. Another important question is whether personal contact and trust is deemed essential for virtual care provision. While decentralized designs are intended to facilitate more personal contact, this study does not show that higher levels of personal contact contribute to improved health outcomes for patients. Future research should examine whether personal contact enhances the effectiveness of care provision in VCCs or whether factors such as quicker response times have a significant impact on patient outcomes.

Existing literature provides inconclusive results on the benefits of VCCs with regard to higher levels of sharing power and responsibility. Previous research has provided evidence for higher levels of sharing power and responsibility as a result of remote patient monitoring [39], where patients are active participants in their treatment decisions [40], whereas other research emphasized that patient empowerment is not improved by digital care provision [41]. Our findings suggest remote monitoring through VCCs fosters the patient's autonomy and their ability to actively engage in decision-making regarding their care provision. However, it introduces a potential bias, as it becomes unclear whether the findings reflect the impact of the VCCs organizational design or are influenced by the pre-existing self-management abilities of patients selected for remote patient monitoring. Consequently, this study cannot claim whether VCCs inherently support the sharing of power and responsibility among patients.

The concept of relational continuity of care offers additional insights into the patient-health care provider relationship, particularly its relevance for trust and support in a therapeutic relationship. Relational continuity, a component of the broader concept of continuity of care, refers to an ongoing therapeutic relationship with the healthcare provider [37]. Moreover,

continuity of carer - a concept in maternal healthcare that refers to care from a consistent and familiar healthcare provider - has been shown to significantly reduce mortality rates, decrease hospital admissions, and improve patient satisfaction [42, 43]. However, the findings of our study suggest that the relational continuity of care may come at risk as a result of VCC care delivery, as patients may be unaware of the specific healthcare providers overseeing their care. Addressing this challenge is essential, particularly in centralized VCCs, where the disconnection between patients and healthcare providers may be more pronounced.

Limitations and future research

This study is not without any limitations. First, this study presents an exclusive focus on the perceptions of healthcare providers, with the patient perspective notably absent. Future research should include patient insights through interviews to assess whether our findings align with their experiences and perceptions. Second, this study only had access to three out of ten VCCs in the Netherlands, potentially limiting the generalizability of our results. Moreover, this study focuses on two distinct VCC designs but does not exclude the possibility of hybrid forms or combinations. Last, a selection bias may be present, as department heads were approached to select eligible individuals to participate using a non-probability sampling method, necessitated by the specific expertise required for meaningful participation. While efforts were made to include participants from diverse professional backgrounds to capture a broad range of perspectives, this limited comparability across groups, as differences in roles may influence perceptions and experiences in ways that are not uniformly comparable.

Conclusion

This qualitative study examined the organizational designs of three VCCs in Dutch hospitals. We identified two distinct organizational designs: decentralized and centralized VCC designs, revealing their operational, collaborative, and patient-healthcare provider relationships variations. Decentralized VCCs foster strong regional collaboration and personal contact, but face scalability challenges, while centralized VCCs optimize resources at scale but struggle with maintaining care integration and personal relationships. Both

organizational designs encounter difficulties in achieving integration across multiple disciplines and maintaining relational continuity of care.

Acknowledgements

The authors would like to thank all participants.

List of abbreviations

**COPD**	Chronic obstructive pulmonary disease
**COREQ**	Consolidated criteria for reporting qualitative studies
**EHR**	Electronic health record
**GP**	General practitioner
**SOP**	Standard operating procedures
**VCC**	Virtual care center

References

1. Hacker K. The burden of chronic disease. Mayo Clinic Proceedings: Innovations, Quality and Outcomes. 2024; 8(1): 112–119. doi:10.1016/j.mayocpiqo.2023.08.005
2. Leenen JPL, Jordens YJ, Wegman A, Heesink L, Lenferink A. Experiences of nurses working in novel virtual care centres in the Netherlands: A qualitative study. BMC Digital Health. 2024; 2(1). doi:10.1186/s44247-024-00127-3
3. Meershoek A, Broek L, Crea-Arsenio M. Perspectives from the Netherlands: Responses from, strategies of and challenges for long-term care health personnel. Healthcare Policy. 2022; 17(SP): 14–26. doi:10.12927/hcpol.2022.26857
4. Tamata AT, Mohammadnezhad M. A systematic review study on the factors affecting shortage of nursing workforce in the hospitals. Nursing Open. 2023; 10(3): 1247–1254. doi:10.1002/nop2.1434
5. World Health Organization. World report on ageing and health. Geneva: WHO; 2015.
6. Paré G, Jaana M, Sicotte C. Systematic review of home telemonitoring for chronic diseases: The evidence base. Journal of the American Medical Informatics Association. 2007; 14(3): 269–277. doi:10.1197/jamia.M2270

7. Semple JL, Sharpe S, Murnaghan ML, Theodoropoulos J, Metcalfe KA. Using a mobile app for monitoring post-operative quality of recovery of patients at home: A feasibility study. JMIR Mhealth and Uhealth. 2015; 3(1): e18. doi:10.2196/mhealth.3929
8. Bashi N, Karunanithi M, Fatehi F, Ding H, Walters D. Remote monitoring of patients with heart failure: An overview of systematic reviews. Journal of Medical Internet Research. 2017; 19(1): e18. doi:10.2196/jmir.6571
9. Koole MAC, Kauw D, Winter MM, Dohmen D, Tulevski II, De Haan R, et al. First real-world experience with mobile health telemonitoring in adult patients with congenital heart disease. Netherlands Heart Journal. 2018; 27(1): 30–37. doi:10.1007/s12471-018-1201-6
10. Kessler R, Casàn-Clará P, Koehler D, Tognella S, Viejo JL, Negro RW, et al. COMET: A multicomponent home-based disease-management programme versus routine care in severe COPD. European Respiratory Journal. 2018; 51(1): 1701612. doi:10.1183/13993003.01612-2017
11. Van Der Burg JM, Aziz NA, Kaptein M, Breteler MJM, Janssen J, Van Vliet L, et al. Long-term effects of telemonitoring on healthcare usage in patients with heart failure or COPD. Clinical eHealth. 2020; 3: 40–48. doi:10.1016/j.ceh.2020.05.001
12. Orozco-Beltrán D, Sánchez-Molla M, Sanchez JJ, Mira JJ. Telemedicine in primary care for patients with chronic conditions: The valcrònic quasi-experimental Study. Journal of Medical Internet Research. 2017; 19(12): e400. doi:10.2196/jmir.7677
13. Steventon A, Bardsley M, Billings J, Dixon J, Doll H, Hirani S, et al. Effect of telehealth on use of secondary care and mortality: findings from the whole system demonstrator cluster randomised trial. BMJ. 2012; 344(jun21 3): e3874. doi:10.1136/bmj.e3874
14. McLean S, Nurmatov U, Liu JL, Pagliari C, Car J, Sheikh A. Telehealthcare for chronic obstructive pulmonary disease: Cochrane Review and meta-analysis. British Journal of General Practice. 2012; 62(604): e739–e749. doi:10.3399/bjgp12X658269
15. De Guzman KR, Snoswell CL, Taylor ML, Gray LC, Caffery LJ. Economic evaluations of remote patient monitoring for chronic disease: A systematic review. Value in Health. 2022; 25(6): 897–913. doi:10.1016/j.jval.2021.12.001
16. Belchior J, Guedes M, António D, Guedes S, Ramos MI, Jafri S, et al. A clinical management centre value in triage of COPD telemonitoring patients. Journal of the International Society for Telemedicine and eHealth. 2017; 5: e7. https://journals.ukzn.ac.za/index.php/JISfTeH/article/view/262

17. Martin-Khan M, Fatehi F, Kezilas M, Lucas K, Gray LC, Smith AC. Establishing a centralised telehealth service increases telehealth activity at a tertiary hospital. BMC Health Services Research. 2015; 15(1). doi:10.1186/s12913-015-1180-x
18. Papoutsi C, A'Court C, Wherton J, Shaw S, Greenhalgh T. Explaining the mixed findings of a randomised controlled trial of telehealth with centralised remote support for heart failure: Multi-site qualitative study using the NASSS framework. Trials. 2020; 21(1). doi:10.1186/s13063-020-04817-x
19. Tong A, Sainsbury P, Craig J. Consolidated criteria for reporting qualitative research (COREQ): A 32-item checklist for interviews and focus groups. International Journal for Quality in Health Care. 2007; 19(6): 349–357. doi:10.1093/intqhc/mzm042
20. Ministerie van Volksgezondheid, Welzijn en Sport. Factsheet telemonitoring bij volwassenen thuis. Inspectie Gezondheidszorg en Jeugd. 2022 [Cited 2024 Jul 10]. Available from: https://www.igj.nl/publicaties/publicaties/2022/02/28/factsheet-telemonitoring-bij-volwassenen-thuis
21. Vesinurm M, Sylgren I, Bengts A, Torkki P, Lillrank P. Concept analysis of patient journey disruptions: The obstacle of integrated care. Journal of Integrated Care. 2024; 32(5): 37–58. doi:10.1108/jica-08-2023-0060
22. Guest G, Bunce A, Johnson L. How many interviews are enough? Field Methods. 2005;18(1):59–82. doi:10.1177/1525822X05279903
23. Edmonds WA, Kennedy TD. An applied guide to research designs: Quantitative, qualitative, and mixed methods. 2nd ed. Thousand Oaks, CA: Sage Publications; 2017. doi:10.4135/9781071802779
24. Jamshed S. Qualitative research method-interviewing and observation. Journal of Basic and Clinical Pharmacy. 2014; 5(4): 87. doi:10.4103/0976-0105.141942
25. Mead N, Bower P. Patient-centredness: A conceptual framework and review of the empirical literature. Social Science & Medicine. 2000; 51(7): 1087–1110. doi:10.1016/S0277-9536(00)00098-8
26. Birt L, Scott S, Cavers D, Campbell C, Walter F. Member checking. Qualitative Health Research. 2016; 26(13): 1802–1811. doi:10.1177/1049732316654870
27. Braun V, Clarke V. Using thematic analysis in psychology. Qualitative Research in Psychology. 2006; 3(2): 77–101. doi:10.1191/1478088706qp063oa
28. Thompson J. A guide to abductive thematic analysis. The Qualitative Report. 2022; 27(5): 1410–1421. doi:10.46743/2160-3715/2022.5340

29. Williams M, Moser T. The art of coding and thematic exploration in qualitative research. International Management Review. 2019; 15(1): 45. https://www.questia.com/library/journal/1P4-2210886420/the-art-of-coding-and-thematic-exploration-in-qualitative
30. Rycroft-Malone J, Fontenla M, Bick D, Seers K. Protocol-based care: Impact on roles and service delivery. Journal of Evaluation in Clinical Practice. 2008; 14(5): 867–873. doi:10.1111/j.1365-2753.2008.01015.x
31. Sinsky CA, Bavafa H, Roberts RG, Beasley JW. Standardization vs customization: Finding the right balance. Annals of Family Medicine. 2021; 19(2): 171–177. doi:10.1370/afm.2654
32. Peters V, Vähätalo M, Meijboom B, Barendregt A, Bok L, De Vries E. Elaborating on modular interfaces in multi-provider contexts. International Journal of Operations & Production Management. 2020; 40(9): 1397–1419. doi:10.1108/IJOPM-12-2019-0822
33. Cao Z, Lumineau F. Revisiting the interplay between contractual and relational governance: A qualitative and meta-analytic investigation. Journal of Operations Management. 2014; 33–34(1): 15–42. doi:10.1016/j.jom.2014.09.009
34. Aben TAE, Van Der Valk W, Roehrich JK, Selviaridis K. Managing information asymmetry in public–private relationships undergoing a digital transformation: The role of contractual and relational governance. International Journal of Operations & Production Management. 2021; 41(7): 1145–1191. doi:10.1108/IJOPM-09-2020-0675
35. Warsen R, Klijn EH, Koppenjan J. Mix and match: How contractual and relational conditions are combined in successful public–private partnerships. Journal of Public Administration Research and Theory. 2019; 29(3): 375–393. doi:10.1093/jopart/muy082
36. Nolte E, Knai C, Hofmarcher M, Conklin A, Erler A, Elissen A, et al. Overcoming fragmentation in health care: Chronic care in Austria, Germany and the Netherlands. Health Economics, Policy and Law. 2012; 7(1): 125–146. doi:10.1017/S1744133111000338
37. Haggerty JL. Continuity of care: A multidisciplinary review. BMJ. 2003; 327(7425): 1219–1221. doi:10.1136/bmj.327.7425.1219
38. Pellegrini CA. Trust: The keystone of the patient-physician relationship. Journal of the American College of Surgeons. 2016; 224(2): 95–102. doi:10.1016/j.jamcollsurg.2016.10.032
39. Schermer M. Telecare and self-management: Opportunity to change the paradigm? Journal of Medical Ethics. 2009; 35(11): 688–691. doi:10.1136/jme.2009.030973

40. Aujoulat I, Marcolongo R, Bonadiman L, Deccache A. Reconsidering patient empowerment in chronic illness: A critique of models of self-efficacy and bodily control. Social Science & Medicine. 2007; 66(5): 1228–1239. doi:10.1016/j.socscimed.2007.11.034

41. Nielsen MK, Johannessen H. Patient empowerment and involvement in telemedicine. Journal of Nursing Education and Practice. 2019; 9(8): 54. doi:10.5430/jnep.v9n8p54

42. Bradford BF, Wilson AN, Portela A, McConville F, Turienzo CF, Homer CSE. Midwifery continuity of care: A scoping review of where, how, by whom and for whom? PLOS Global Public Health. 2022; 2(10): e0000935. doi:10.1371/journal.pgph.0000935

43. Pace CA, Crowther S, Lau A. Midwife experiences of providing continuity of carer: A qualitative systematic review. Women and Birth. 2021; 35(3): e221–e232. doi:10.1016/j.wombi.2021.06.005

Supporting information

S1 File. A 32-item checklist for reporting qualitative studies (COREQ).

S2 File. Interview topic guide.

Support Information 1

Click here to access/download
Supporting Information
S1_File.docx

Supporting Information 2

Click here to access/download
Supporting Information
S2_File.docx
