# Supplementary material for: The organization of virtual care centers: A qualitative study in Dutch hospitals
Source: PLOS Digit Health. 2026 Jun 26;5(6):e0001479. doi: 10.1371/journal.pdig.0001479 (PMC13308795; doi:10.1371/journal.pdig.0001479)
Supplement: Attachment — Submitted filename: 20260421 Response letter.docx [file pdig.0001479.s006.docx]

Reviewer 1

**Q1. Thank you for the opportunity to review the revised version of this manuscript. The authors have clearly taken the previous comments seriously and have responded to them in a thoughtful and constructive manner. Overall, the revisions have significantly improved the manuscript. The conceptual framing is clearer, the internal consistency of the study aim and discussion has been strengthened, and the methodological transparency has been enhanced. In particular, the clarifications regarding terminology, the positioning of the study, and the articulation of implications have made the argumentation more coherent and easier to follow. While in some instances the additional elaborations could perhaps have been developed in greater depth, I acknowledge the constraints of word limits and consider that the authors have made a sincere and substantial effort to address the concerns raised in the previous review. The manuscript is now clearer, more internally consistent, and better positioned within the broader scholarly conversation. In my view, the paper has improved meaningfully through the revision process and is suitable for publication. Recommendation: Accept.**

R. We thank the reviewer for the kind words and for acknowledging the relevance and timeliness of our study. We appreciate the constructive feedback provided, which helped us clarify and strengthen several conceptual and methodological aspects of the manuscript.

Reviewer 2

**Q2. My initial review concerns have been fully addressed.**

R. We sincerely thank the reviewer for their time and for confirming that the concerns raised in the initial review have been fully addressed. We appreciate the constructive feedback provided throughout the review process, which has contributed meaningfully to strengthening the manuscript.

Reviewer 3

**Q3. The revised manuscript demonstrates clear and substantive improvements. In particular, the conceptual distinction between centralised and decentralised VCC designs is now more consistently integrated across the Background, Methods and Results sections, and the Discussion has been strengthened in terms of structure and analytical coherence. The authors have responded constructively to most substantive concerns. However, several issues remain that require clarification in order to ensure methodological transparency and conceptual robustness. These relate to the theoretical anchoring of the study, the reporting of FTE data, the description of the coding process, and the explicit reporting of interviewer positionality in the main text.**

R. We thank the reviewer for this positive and constructive evaluation of the revised manuscript. We appreciate the recognition of the substantive improvements made, particularly regarding the integration of the conceptual distinction between centralized and decentralized VCC designs and the strengthened analytical coherence of the Discussion section. We also appreciate the reviewer’s continued careful attention to the remaining points requiring clarification. The comments regarding theoretical anchoring, FTE reporting, coding transparency, and interviewer positionality are relevant, and we address each of these comments in more detail below.

**Q4. Although additional references to organisational design theory have been incorporated, the manuscript still lacks a clearly articulated analytical framework. The distinction between centralised and decentralised VCC designs remains largely descriptive rather than explicitly grounded in established theoretical dimensions (e.g. coordination mechanisms, governance structures, standardisation versus mutual adjustment). The authors should more clearly specify how their empirical findings are theoretically informed and how they contribute to organisational design theory beyond citation.**

R. We thank the reviewer for this comment. To clarify the theoretical grounding, we explicitly link our empirical findings to theory in the Discussion section. We now establish a grounded connection with the literature on decentralization and centralization, building on manufacturing literature which shows the perks of (de)centralized warehouse locations.

*C. “This study shows the operational implications of two distinct organizational VCC designs and highlights a fundamental tension between localized, hospital-specific operations in decentralized VCCs and standardized processes that regulate centralized VCC designs. The empirical findings show that in decentralized VCCs, the programs for the included care pathways are collaboratively developed and evaluated within the hospital, reflecting regional standards of care and policies. In contrast, centralized VCCs leverage standardized protocols to achieve scalability, optimize resources, and enable the management of a broader patient base. (p.19, line 475-482)*

*This tension mirrors a debate in logistics and distribution literature, which conceptualizes the selection of a distribution structure as a multi-dimensional trade-off influenced by an interplay of product characteristics, supply-side constraints, such as labor and land availability, and contextual factors like regional policy divergence and digital infrastructure [32]. While centralization is traditionally favored to minimize inventory costs and exploit risk-pooling effects [33], efficiency gains are not warranted. Although centralized structures aim to minimize total system costs through coordinated replenishment, the increasing number of participating warehouses, VCCs in our context, can escalate coordination complexity, potentially leading to sub-optimal performance compared to decentralized models where individual units remain responsive to local cost structures [34]. Consequently, decentralization can be the preferred strategic orientation when demand dispersion increases or when local responsiveness and alignment with regional regulations are prioritized [32]. Crucially, while centralization mitigates demand fluctuations, decentralization offers resilience through risk diversification, maintaining independent warehouse locations to safeguard against regional disruptions or supply chain failures [35]. (p.19, line 483-498)*

*Both organizational VCC designs are characterized by a reliance on program-based care pathways, which may be inherited from traditional care models where protocol-based care guides care delivery [36], reinforcing a siloed approach in which each discipline operates independently.” (p.20, line 506-509)*

Additional references:

[32] Onstein AT, Tavasszy LA, Van Damme DA. Factors determining distribution structure decisions in logistics: A literature review and research agenda. Transport Reviews. 2019; 39(2): 243–260. doi:10.1080/01441647.2018.1459929

[33] Shen ZJM, Coullard C, Daskin MS. A joint location-inventory model. Transportation Science. 2003; 37(1): 40–55. doi:10.1287/trsc.37.1.40.12823

[34] Abdul-Jalbar B, Gutiérrez J, Puerto J, Sicilia J. Policies for inventory/distribution systems: The effect of centralization vs. decentralization. International Journal of Production Economics. 2003; 81: 281–293. doi:10.1016/S0925-5273(02)00361-4

[35] Schmitt AJ, Sun SA, Snyder LV, Shen ZJM. Centralization versus decentralization: Risk pooling, risk diversification, and supply chain disruptions. Omega. 2015; 52: 201–212. doi:10.1016/j.omega.2014.06.002

**Q5. The presentation of “FTE working in VCC (n)” requires clarification. It appears unlikely that all staff members are employed at 1.0 FTE within the VCC, and the number of employees and the number of FTEs represent conceptually distinct parameters. The authors should clarify whether these figures reflect aggregated FTE calculations or simply the number of staff, and explicitly explain how the FTE values were derived.**

R. We thank the reviewer for this important observation. The numbers reported in this column reflect the aggregated full-time equivalents (FTEs) working within each VCC rather than the absolute number of staff members. These values represent the total contracted time allocated to VCC-related activities across all involved staff and were provided by the department head or manager of each respective VCC, based on their internal operational records. To make this distinction explicit, we revised the table header from “FTE working in VCC (n)” to “Aggregated FTE working in VCC (FTE)”. In addition, we standardized the numerical presentation (e.g., 30 to 30,0) and added a table note to Table 1 specifying the data source and reporting year.

*C. “Aggregated FTE working in VCC (FTE)” (p.5, Table 1)*

*“Note: The data were collected in 2025 and retrieved from the department head or manager for each respective VCC.” (p.5, line 118-119)*

**Q6. While reference is made to COREQ in the supplementary material, essential information regarding the interviewer(s) should also be included in the main text. The manuscript should explicitly state who conducted the interviews, their professional background, whether they had prior relationships with participants, and how potential bias or positionality was addressed. Reliance solely on supplementary reporting is insufficient for full methodological transparency.**

R. We thank the reviewer for this important comment regarding methodological transparency. In response, we have expanded the main text in the Methods section to explicitly state that the interviews were conducted by the first author [Omitted for review purposes]. We also clarified that the interviewer (first author) had no prior relationship with the any of the respondents. To further strengthen transparency around positionality and potential bias, we added a reflection in the Limitations section noting that, because participants were recruited through department heads and managers of the VCCs, some degree of selection bias may have been introduced.

*C. “A diverse group of sixteen participants with no prior relationship to the interviewer [Omitted for review purposes] was initially approached, with twelve ultimately included in the study, encompassing a wide range of professional expertise (Table 2)” (p.6, line 126-129)*

*“Semi-structured interviews were conducted by [Omitted for review purposes] in April and May 2024 in both face-to-face and online settings, lasting between 27-42 minutes each.” (p.6, line 138-139)*

*“Furthermore, recruiting participants through department heads and managers may have introduced selection bias. Combined with differences in respondents’ professional roles, this may have influenced the perspectives captured and consequently limit the generalizability of the findings.” (p.22, line 623-626)*

**Q7. The description of the thematic analysis remains too general. The statement that coding consistency was ensured through peer meetings does not sufficiently clarify whether double coding was conducted, who performed the coding, how disagreements were resolved, or whether a formal codebook was developed. A more detailed account of the coding process is required to strengthen methodological rigour and transparency.**

R. We thank the reviewer for this important suggestion. To improve methodological transparency, we added a more detailed description of the coding process to the Methods section, clarifying who performed the initial coding, how the coding framework was independently reviewed, how discrepancies were resolved through consensus among all three authors, and how the resulting definitive coding framework was subsequently applied to all twelve transcripts.

*C. “The initial coding of the first three interviews was conducted by [Omitted for review purposes], after which both the coding framework and the coded transcripts were independently reviewed by [Omitted for review purposes]. Any discrepancies were discussed among all three authors until consensus was reached. This process resulted in a definitive coding framework, which was subsequently applied by [Omitted for review purposes] to all transcripts.” (p.7, line 162-167)*

**Q8. The manuscript states that secondary data (e.g. brochures and policy documents) were collected and used to support interpretation. However, it remains unclear how these documents were analysed, whether they were coded alongside the interview data, and how they contributed to the development of themes. The authors should specify the analytical approach applied to the document analysis and clarify how (and where) these data are reflected in the Results. Without such clarification, the role of document analysis in the overall study design remains insufficiently transparent.**

R. We thank the reviewer for this helpful comment. We clarified in the Methods section that the additional documents (e.g., brochures and folders) were obtained solely to better understand the organizational context and design of the respective VCCs. These materials were not formally coded and were not used in the thematic analysis or development of the results.

*C. “Relevant documents such as brochures and folders were obtained from department heads and managers after the interviews had taken place to provide a clearer understanding of the study context. These materials were used solely for contextual insights and were not included in the data analysis.” (p.7, line 147-150)*
