# Supplementary material for: The organization of virtual care centers: A qualitative study in Dutch hospitals
Source: PLOS Digit Health. 2026 Jun 26;5(6):e0001479. doi: 10.1371/journal.pdig.0001479 (PMC13308795; doi:10.1371/journal.pdig.0001479)
Supplement: Attachment — Submitted filename: 20260121 Response letter PLOS.docx [file pdig.0001479.s005.docx]

Reviewer 1

**General Evaluation
Thank you for the opportunity to review this interesting and timely manuscript examining the organizational designs of virtual care centres (VCCs) in Dutch hospitals. The topic is clearly relevant for PLOS Digital Health and contributes to our understanding of how digital health services are structured and managed in practice.** **Overall, the paper is well written and provides valuable empirical insight into a field that remains underexplored. My comments aim to help the authors further clarify some conceptual and methodological aspects and to strengthen the paper’s positioning and coherence. I would recommend minor revisions before the paper can be considered for publication.**

R. We thank the reviewer for the kind words and for acknowledging the relevance and timeliness of our study. We appreciate the constructive feedback provided, which helped us clarify and strengthen several conceptual and methodological aspects of the manuscript.

**Q1. Conceptual clarity – Definition of remote patient monitoring (RPM)
The authors define remote patient monitoring (RPM) as “supervision and guidance of patients through the remote monitoring of vital indicators and symptoms.” However, this definition seems conceptually inconsistent. Monitoring refers to the collection and observation of patient data (e.g., vital signs, symptoms) rather than the direct provision of care or supervision. In several parts of the manuscript, the authors describe activities such as nurses monitoring alerts, offering support, and enabling remote care delivery. These actions go beyond monitoring and clearly belong to the realm of remote patient care or remote patient management. I would recommend that the authors reconsider the terminology used throughout the paper. If the focus is on clinical engagement and support provided remotely, terms such as remote patient care or remote patient management might be more appropriate. Alternatively, if they wish to keep the RPM terminology, they should explicitly explain that they apply the concept in a broader sense, covering both monitoring and professional interventions. Furthermore, the cited definition (from 2007) appears outdated given the rapid conceptual and technological evolution of telehealth. More recent definitions could be used to provide a more contemporary framing of the concept. Clarifying this distinction would significantly improve the conceptual rigor of the paper and strengthen its theoretical foundation.**

R. Thank you for this suggestion. A timelier definition better reflects the current state of remote patient monitoring. We have therefore incorporated an updated definition of remote patient monitoring, including an addition to the reference list. In addition, the definition clarifies that our use of RPM encompasses both monitoring and professional interventions to reflect the broader scope of activities observed in practice.

*C. RPM uses digital technologies to collect medical and physiological data from patients outside clinical settings and transmit it to health care providers for assessment and guidance [6]. (p.4, line 75-77)*

Additional reference:

[6] Farias FA, Dagostini CM, Bicca YA, Falavigna VF, Falavigna A. Remote patient monitoring: A systematic review. Telemedicine and e-Health. 2020; 26(5): 529–225. doi:10.1089/tmj.2019.0066.

**Q2. Definition and contextual scope of “virtual care centres”
The authors define virtual care centres as facilities focusing on postoperative monitoring at home and long-term disease management. While this apparently reflects the current use of the term in the Dutch healthcare context, it is important to note that the concept of virtual care centres varies internationally. In many other countries (for example Finland, Canada, and the UK), virtual care centres or virtual health centres typically provide a much broader range of primary and preventive care services, including medication renewals, symptom-based triage, remote consultations, and self-care guidance. To enhance conceptual clarity and international relevance, I recommend that the authors explicitly state that their definition applies to the Dutch healthcare setting, and briefly acknowledge that alternative virtual care centre models exist globally. This clarification would help readers interpret the study’s findings within the appropriate national and organizational context.**

R. Thank you for this suggestion. We updated the manuscript to specify that VCCs provide perioperative, postoperative, episodic, and chronic forms of remote patient monitoring, offering a more comprehensive overview of their functionalities. In addition, we now clarified how the literature addresses international VCCs and clarified that this study focuses on the Dutch healthcare context, and we acknowledge that other VCC models exist internationally.

*C.* *VCCs facilitate a mix of digital and physical care - for perioperative, postoperative, episodic, and chronic patients – through RPM (p.4, line 74-77).*

*Virtual care centers (VCCs) facilitate remote patient monitoring (RPM) at the hospital level in the Netherlands. (p.4, line 72-72)*

*However, while VCCs are becoming common practice in hospitals, they remain underrepresented in academic literature. Existing studies have described related models of RPM at the ward level, such as clinical management centers in Portugal [15], telehealth centers in Australia [16], virtual wards in the United Kingdom [17], virtual clinics in the United States [18], and medical command centers in Sweden [19]. [p. 4, line 98-102)*

*Furthermore, VCCs in the Netherlands are primarily shaped by clinical and administrative judgement, accelerated by the COVID-19 pandemic. (p.5, line 105-107)*

Additional references (17, 18, and 19):

[17] NHS England. (n.d.). *What is a virtual ward?* https://www.england.nhs.uk/virtual-wards/what-is-a-virtual-ward/ [Cited 2026 Jan 20].

[18] Mayo Clinic Health System. (n.d.). *Virtual care options*. https://www.mayoclinichealthsystem.org/virtual-care [Cited 2026 Jan 20].

[19] Kastengren, M., Frisk, L., Winterfeldt, L., Wahlström, G., & Dalén, M. (2025). Implementation of Sweden's first digi‑physical hospital‑at‑home care model for high‑acuity patients. Journal of Telemedicine and Telecare, 31(6), 891–897. https://doi.org/10.1177/1357633X241232176

**Q3. Organizational perspective on RPM implementation
The authors rightly point out that previous studies have predominantly treated remote patient monitoring (RPM) as a technological intervention, often overlooking the importance of organizational design in its implementation. This is an important and valuable observation that adds theoretical depth to the study.**

R. Thank you for the kind words. We now clarify that the primary contribution of this study is to examine organizational designs, as previous literature has largely focused on RPM as a technological intervention rather than considering the organizational configurations that shape implementation.

*C. Despite these findings, existing literature conceptualizes RPM as a technological intervention, overlooking the importance of the organizational design in its implementation. (p.4, line 93-95)*

**Q4. In organizational and health management literature, it is well established that different organizational configurations—such as centralized or decentralized models—can substantially influence the outcomes and efficiency of technology-enabled care delivery. These configurations affect not only workflow and coordination, but also resource allocation, scalability, and ultimately the economic sustainability of such interventions. Although this paper does not address economic outcomes directly, it would be worthwhile to discuss how the chosen organizational design impacts cost-effectiveness and operational efficiency. Including this perspective in subsequent studies could broaden the understanding of how organizational structures shape the realized benefits of RPM.**

R. Thank you for the suggestion regarding future research. We have incorporated a sentence in the manuscript suggesting that subsequent studies should examine how organizational design choices impact cost-effectiveness and operational efficiency, thus broadening the understanding of how organizational structures of a VCC influence RPM outcomes.

*C. Moreover, subsequent studies should examine how organizational design choices impact cost-effectiveness and operational efficiency, broadening the understanding of how organizational designs of VCCs influence RPM outcomes. (p.23, line 624-627)*

**Q5. Study aim and alignment with the stated research gap
The statement of the study’s aim appears inconsistent across sections. In the Background, the authors write that the aim is “to examine organizational designs of VCCs in Dutch hospitals,” whereas the Abstract refers to exploring and characterizing their design to provide a clearer understanding of their structure and implications. In the Author Summary, the authors claim to address a gap concerning the lack of empirical evidence on organizational differences and practical implications of VCCs. These formulations are related but not fully equivalent. The first (“to examine organizational designs”) describes a rather descriptive purpose, while the latter two imply an analysis of implications and consequences of organizational variation. As currently formulated, it is not entirely clear whether the presented aim allows the authors to address the stated research gap. I would recommend harmonizing the aim statement across all sections and clarifying whether the study intends merely to describe existing organizational models or also to analyze their practical implications. If the latter is intended, the aim could be expanded accordingly. This clarification would strengthen the internal consistency and conceptual coherence of the paper.**

R. Thank you for this observation. We clarified the aim of this paper and focused explicitly on examining the different organizational designs of VCCs. We have harmonized the wording throughout the manuscript to ensure consistency and removed any ambiguous statements regarding implications, ensuring that the study aim aligns with the focus on organizational designs.

*C. This study aims to examine the organizational designs of VCCs to provide a clearer understanding of their structure and implications. (p.2, line 34)*

*Despite the growing implementation of both designs, empirical research on their organizational differences remains limited. (p.3, line 65-67)*

**Q6. Selection of organizations and sampling rationale**
**The study includes three virtual care centres (VCCs) selected from a total of ten operating in the Netherlands. The authors note that the selection was based on purposeful sampling, considering the number of patients and care pathways. However, the rationale for including these specific three centres—and excluding the remaining seven—is not fully explained. The current description of the sampling procedure does not provide sufficient clarity or transparency to understand why these organizations were chosen. Readers are left unsure whether the selection reflects diversity in organizational models, data accessibility, or other contextual factors. Providing a more explicit explanation of how and why these organizations were selected from the ten possible ones would strengthen the methodological transparency and help readers assess the robustness of the findings. I recommend that the authors elaborate on the selection criteria and briefly indicate how the chosen organizations relate to the broader landscape of Dutch VCCs. This clarification would improve the study’s credibility and interpretability.**

R. Thank you for this suggestion. We clarified the organizational selection process and explicitly described the two criteria used in our study: (1) the number of patients receiving remote care, reflecting organizational scope, and (2) the included care pathways, defined as structured plans integrating clinical procedures and supportive services. This clarification provides transparency and situates the selected VCCs within the broader landscape of Dutch virtual care centres.

*C. Three VCCs were included in our study – A, B, and C – based on two criteria. The first criterion was the number of patients receiving digital care, indicating the scope. The second criterion concerned the range of care pathways provided, defined as structured care plans that integrate clinical interventions with supportive digital services for the management of specific patient populations [23] (Table 1). (p.5, line 127-130)*

**Q7. Scope, duration, and validation of interviews
The manuscript states (S2 fine) that seven broad thematic areas were covered in the interviews, each consisting of multiple sub-questions, and that each interview lasted approximately 30 minutes. While the authors mention that participants were given the opportunity to review their transcribed interviews—and that four participants made minor corrections—this process primarily ensures transcriptional accuracy rather than substantive expansion or deepening of the discussion.**

R. We apologize for the confusion. The primary purpose of having participants review their transcripts was to ensure transcriptional accuracy, not to deepen the discussion. As the transcripts reflect only the interview content without interpretation, only minor textual corrections were made, which was already incorporated in the manuscript. Hence, no adjustment has been made to the manuscript.

**Given the complexity of the topic, a 30-minute timeframe appears rather limited for exploring multiple themes in sufficient depth. Although this issue cannot be remedied retrospectively, it may be worth acknowledging as a limitation. The authors might briefly note that time constraints and the professional context of the participants could have restricted opportunities for reflection and elaboration, which is a common challenge in qualitative research conducted in clinical environments. Including such a note would demonstrate methodological reflexivity and transparency.**

R. Regarding interview length, the average duration was approximately 30 minutes, with a range between 27–42 minutes. Since interviews were conducted until all themes were adequately covered, we did not consider time constraints as a limitation. We clarified the length of the interviews and added an extra column in Table 2. Moreover, we made a minor adjustment to S1 (COREQ) to update the interview length of the interviews.

*C. Semi-structured interviews were conducted in April and May 2024 in both face-to-face and online settings, lasting between 27-42 minutes each. (p.7, line 155)*

*Each interview lasted between 27-42 minutes. (S1 File, p.2)*

**Q8. Emergence of organizational models in the analysis
The authors state that the analysis led to the identification of two distinct organizational designs—centralized and decentralized. However, it remains somewhat unclear whether this distinction truly emerged from the analytical process or whether it was already evident from the selection of organizations. Given that the included VCCs differed substantially in scale (e.g., 600 vs. 23,000 patients) and operational scope (local vs. national), readers may wonder whether the centralized and decentralized models were at least partially known or anticipated prior to the analysis. If this was the case, the analysis may have confirmed or elaborated on an existing assumption rather than generating the distinction inductively. I would suggest clarifying whether the identification of these two organizational models was an outcome of the data analysis or whether it represents a theoretically informed categorization established earlier in the research design. This clarification would enhance the transparency and interpretive rigor of the methodological account.**

R. We appreciate the comment. While we were aware of differences between organizational designs, the distinction between centralized and decentralized models was not predetermined. The categorization emerged inductively from the empirical analysis, rather than being theoretically imposed prior to data collection.

*C.* *Two distinct organizational designs for VCCs have emerged from the data: decentralized designs, embedded within individual hospitals, and centralized designs, which coordinate care across multiple hospitals. (p.2, line 39-42)*

*Two distinct organizational VCC designs emerged from the data: decentralized and centralized designs. (p.9, line 188-189)*
 **Q9. Clarity and consistency of the study’s implications
The term implications is used in several parts of the manuscript—in the Author Summary (“practical implications”), in the Data Analysis section (“specific implications”), and again in the Discussion (“operational implications”). However, it remains somewhat unclear what type or level of implications the study ultimately aims to produce. Are these implications primarily operational, practical, managerial, organizational, or theoretical in nature?

This ambiguity also relates to the earlier concern about the study’s stated aim: if the primary contribution is to clarify the organizational designs of VCCs, the authors should specify what implications arise from this clarification and for whom (e.g., healthcare managers, clinicians, policymakers).**

R. We revised the manuscript for clarity and consistency. In the Author Summary, references to “practical implications” have been removed, focusing instead on organizational differences. In the Data Analysis section, “specific implications” has been replaced with “features” to accurately reflect the descriptive content. Operational implications remain in the Discussion section, as intended, to address how organizational design affects care processes.

*C. “We then identified themes that captured the characteristics and features of these organizational designs. The first theme captured the operational implications of VCCs […]. (p.7-8, line 176-178)*

**Q10. I recommend that the authors more explicitly articulate the main implications in the Discussion and summarize them clearly in the Conclusion. A short synthesis highlighting what the study contributes in terms of actionable or conceptual insights would strengthen the paper’s coherence and readability.**

R. Thank you for this suggestion. We have highlighted the main implications in the discussion section and clarified their contribution to the existing literature. To avoid redundancy, we did not modify the conclusion any further, as it provides a concise summary of the key findings.

*C. This study examined the organizational designs of VCCs in the Netherlands and identified a centralized and decentralized design. The distinction between these two designs is not rigid, hybrid forms may emerge that incorporate elements of both. The findings were categorized into three themes: 1) the operational implications, 2) collaboration between healthcare providers, and 3) the patient-healthcare provider relationship, which appeared across both designs. (p.19, line 475-479)*

**Q11. Reflection on selection bias in the Limitations section
The authors appropriately acknowledge a potential selection bias related to the recruitment of interview participants by department heads. It might also be valuable to extend this reflection to the earlier stage of organizational selection. As noted previously, only three out of ten Dutch VCCs were included in the study, but the rationale for their selection remains somewhat unclear.

A brief acknowledgement that selection bias could also apply to the organizational level—depending on how the participating centres were identified and approached—would further strengthen the transparency of the Limitations section. This addition would show awareness of potential sources of bias across multiple levels of the research process.**

R. We apologize for the oversight. We have added a sentence acknowledging potential selection bias at the organizational level, noting that including only three out of ten Dutch VCCs may have constrained the observed range of VCC designs. We recommend that future research includes a broader sample of VCCs to enhance generalizability.

*C. First, this study included only three out of ten VCCs in the Netherlands, potentially limiting the generalizability of our results. (p.23, line 620-622)*

**Q12. Positioning and value of interdisciplinarity
The study clearly integrates elements from several academic domains, including organizational and management studies, operations management, nursing science, and digital health technology research. This interdisciplinary nature is one of the paper’s strengths and reflects the multifaceted reality of virtual care delivery. However, it may be helpful for readers if the authors could more explicitly articulate how they position their study within a particular scientific conversation or discipline. At present, the discussion remains somewhat general, and the reference list does not show a clear concentration in any specific field. Clarifying the disciplinary lens could strengthen the conceptual anchoring and the perceived scholarly contribution. At the same time, the interdisciplinary scope of the work adds important value: it enables a more holistic understanding of a complex socio-technical phenomenon, bridges theoretical and practical perspectives, and fosters dialogue between disciplines that are often studied in isolation. Briefly highlighting this integrative contribution, perhaps in the Discussion or Conclusion, would underline both the novelty and the broader relevance of the paper.**

R. We thank the reviewer for the comment. We clarified the interdisciplinary nature of our study in the Discussion, highlighting contributions from operations management and digital health research. We emphasize that this integrative approach allows for a holistic understanding of virtual care delivery and bridges theoretical and practical perspectives, underlining both the novelty and broader relevance of the study.

*C.* *This study addresses a research gap in the literature, as organizational designs of VCCs have not previously been empirically examined beyond RPM at the ward level [15-19]. Our study bridges operations management and digital health disciplines, as the VCC designs possess elements related to processes and coordination, both between patients and healthcare professionals. The discussion positions these findings within the broader organizational design literature, showing how they align with and extend existing concepts. (p.19, line 480-485)*

**Final Recommendation**

**This is a promising and well-executed manuscript that makes a relevant contribution by addressing organizational design aspects of virtual care centres. With minor revisions focusing on conceptual clarity, methodological transparency, and explicit positioning, the paper will be well suited for publication in PLOS Digital Health.**

R. We thank the reviewer for the positive outlook and constructive suggestions. We have addressed the points raised, clarified conceptual definitions, improved methodological transparency, and highlighted the interdisciplinary value of the study. These revisions have strengthened the manuscript and its contribution to understanding organizational designs of virtual care centers.

Recommendation: Accept with minor revisions.

Reviewer 2

**I would like to sincerely thank the editors for the opportunity to review this manuscript on the Organization of Virtual Care Centers. The paper represents a high-quality scientific contribution that addresses a topic of both substantial practical importance and international academic relevance. The authors provide a well-structured and comprehensive discussion of organizational models, governance, and care delivery processes within Virtual Care Centers, offering valuable insights for the advancement of modern healthcare systems. The selection of this journal as the publication venue is, in my opinion, highly appropriate. The paper fits well within its thematic scope and will be of great interest to a broad readership in health services research, digital health policy, and clinical innovation.**

R. We sincerely thank the reviewer for the positive and encouraging evaluation of our manuscript. We greatly appreciate the recognition of the paper’s contribution to understanding organizational models, governance, and care delivery processes.

**Q13. From a holistic perspective, the manuscript is scientifically sound, methodologically robust, and clearly written. However, one important aspect deserves further consideration: the data infrastructure that underpins telemonitoring and digital care processes. This element is mentioned only briefly, though it constitutes a fundamental and enabling pillar for any sustainable and scalable Virtual Care Center. For future refinement of the paper, I would strongly encourage the authors to elaborate on this dimension and to discuss its role in ensuring interoperability, data quality, and secure information exchange. Moreover, it may be worthwhile to explicitly include — in the conclusion section — a statement advocating for the establishment of interoperable data infrastructures as a prerequisite for the further development of telemonitoring and integrated virtual care.**

R. Thank you for highlighting this important aspect. We have expanded the discussion of data infrastructure within the “Operational Implications” section, elaborating on system integration, file accessibility, and the role of interoperable digital systems in supporting sustainable and scalable virtual care centers. Since we elaborate on this topic in the Discussion section now, we did not include an additional statement in the conclusion to prevent unnecessary repetition.

*C. Moreover, the empirical findings suggest that both decentralized and centralized organizational designs face persistent challenges related to data infrastructure. Respondents emphasized that data accessibility and interoperable digital systems are essential for the effective functioning of a VCC, with these concerns being even more pronounced in centralized designs. These findings align with existing literature, which highlights that limited interoperability and constraints in EHR accessibility hinder benefits of digital technology such as usability, information exchange, and advanced data analytics [35]. The empirical findings and existing literature emphasize the importance of interoperability and data accessibility. (p.20, line 513-521)*

**Further detailed comments and specific suggestions can be found in the attached annotated PDF**.

**Q14. Line 56 - deigns**

R. Thank you for this suggestion. The typographical error has been corrected to “designs.”

*C. To explore the different characteristics of the two organizational designs, these themes were derived. (p.2, line 63)*

**Q15. It would be good if the incidence of VCC were described here, preferably also internationally. In conjunction with the generalizability of the results (in limitations), this could possibly show that the Netherlands is a very representative country for such a survey.**

R. We clarified that the context of this study relates specifically to the Dutch context. We acknowledged that VCCs exist internationally and mention existing studies, providing a broader perspective on virtual care centers while situating our findings within the Dutch healthcare system.

*C. Virtual care centers (VCCs) facilitate remote patient monitoring (RPM) at the hospital level in the Netherlands […]. (p.4, line 71-72)*

*However, while VCCs are becoming common practice in hospitals, they remain underrepresented in academic literature. Existing studies have described related models of RPM at the ward level, such as clinical management centers in Portugal [15], telehealth centers in Australia [16], virtual wards in the United Kingdom [17], virtual clinics in the United States [18], and medical command centers in Sweden [19]. Furthermore, VCCs in the Netherlands are primarily shaped by clinical and administrative judgement, accelerated by the COVID-19 pandemic. As a result, the designs of VCCs are not evidence-based, although such approaches in organizational design theory have been shown to be important for ensuring patient safety and staff satisfaction [20]. Hence, this research provides the first empirical analysis of organizational designs of VCCs in the Netherlands, addressing a gap in the literature where prior work has only presented RPM at the ward level. (p.4-5, line 98-112)*

**Q16. Table 4: Here it is not clear, if it is given or not - please add this information**

R. Thank you for this observation. We revised the text in Table 4. The revised text emphasizes that these capabilities are currently needed rather than fully implemented, reflecting the situation at hand described by participants.

*C.* *Comprehensive national-level system integration is required to ensure seamless data exchange and uniform data accessibility across all hospitals via shared digital platforms. (p.10, Table 4)*

**Q17. (Line 281) Here, it would be good to clarify which data is available across hospitals and which is not. Particularly in conjunction with the topic of alarm monitoring above, readers have assumed up to this point that vital data in particular is available. In this section, however, one gets the impression that a lot of data is missing. I would recommend differentiating between the type of data and the degree of transfer, at the latest in the discussion of the results.**

R. Thank you for this comment and we apologize for the confusion. First, we replaced the heading ‘system integration’ by ‘system interoperability, as this concept better reflects the idea that systems do not need to be fully integrated but must be able to communicate with one another. In addition, we clarified the type of data being referred to by adding an explanatory sentence.

*C.* *Importantly, this did not reflect missing clinical data - most patient data, including vital signs, were generally available - but rather challenges related to how and when these data could be accessed and integrated across organizational boundaries (p.13, line 293-296)*

**Q18. (Line 305**) **‘Package’ is difficult to understand. Perhaps it is colloquial language?**

R. Thank you for this suggestion. We have replaced the colloquial term “package” with “an integrated set of care services,” which conveys the intended meaning of coordinated and comprehensive care provision.

*C.* *Offering one integrated set of care provision is less easy to organize at a national [centralized] VCC, because it covers a larger area. (p.15, line 342)*

**Q19. Line 323 - Correction to: cardiologist**

R. Thank you. We have made the correction.

*C. cardiologist (p.15, line 360)*

**Q20. (Line 343) Since ‘the app’ is now mentioned more frequently in the text, it would be important for the reader to explain exactly what ‘the app’ is, at the latest in the discussion or even in the background, where telemonitoring is described very good.**

R. Thank you for this comment. We have replaced all instances of “the app” with “mobile application”, consistent with the terminology used in the Background section.

*C. They are currently working with different mobile applications, different communication modules, and different EHRs. (p.13, line 282-283)*

*We should build in that multiple programs can be combined in the mobile application. (p. 15, line 367-368)*

*Many patients initially are afraid for losing contact with their doctor. But eventually, they realize that through the mobile application, they can actually have quicker contact when needed. (p.16, line 380-382)*

**Q21. This section lacks discussion of ‘system integration and file accessibility’ – please add this, as data transfer is a very important organizational pillar in telemonitoring that should not be overlooked when comparing organizational models. The requirement for a homogeneous data infrastructure, especially in centralized structures, could even be an important conclusion of this study.**

R. Thank you for this suggestion. We added a section (in the Discussion section) discussing system interoperability and data accessibility. In doing so, we replaced the term ‘system integration’ by ‘system interoperability’ and ‘file’ by ‘data’, to more accurately reflect the concepts. We also emphasized, in line with both the empirical findings and the existing literature, that a robust digital infrastructure is essential, especially for centralized VCCs, where consistent data availability and seamless information exchange are critical.

*C.* *Moreover, the empirical findings suggest that both decentralized and centralized organizational designs face persistent challenges related to data infrastructure. Respondents emphasized that data accessibility and interoperable digital systems are essential for the effective functioning of a VCC, with these concerns being even more pronounced in centralized designs. These findings align with existing literature, which highlights that limited interoperability and constraints in EHR accessibility hinder benefits of digital technology such as usability, information exchange, and advanced data analytics [35]. The empirical findings and existing literature emphasize the importance of interoperability and data accessibility. (p.20, line 513-521)*

Additional references:

[35] Howe, J. L., Adams, K. T., Hettinger, A. Z., & et al. (2018). Electronic health record usability issues and potential contribution to patient harm. JAMA, 319(12), 1276–1278. https://doi.org/10.1001/jama.2018.1171

**Q22: (Line 538) See my comment in the first part - a correction could possibly mitigate this limitation.**

R. We have incorporated the reviewer’s earlier comment (Q9) regarding selection bias and clarified it in this section. We acknowledge that including only three of ten Dutch VCCs may limit generalizability, and we suggest that future research should examine a broader sample to capture a wider range of potential organizational designs.

*C. First, this study included only three out of ten VCCs in the Netherlands, potentially limiting the generalizability of our results. Future research should examine more VCC designs. (p.23, line 619-621)*

**Reviewer 3**

**General: This manuscript presents a well-written and timely qualitative study examining the organizational designs of Virtual Care Centers (VCCs) in Dutch hospitals. The topic is highly relevant given the rapid digitalization of healthcare delivery. The authors identify and compare two distinct organizational models, centralized and decentralized, offering valuable empirical insights into how these designs affect collaboration and patient- provider relationships. Overall, the paper is clear and logically structured** **but several substantive improvements are needed to strengthen its conceptual clarity and analytical coherence. Most importantly, the distinction between centralized and decentralized VCC designs requires clearer theoretical definition and consistent integration throughout the Background, Methods, and Results, as it currently appears both predetermined and emergent. The thematic structure would benefit from clearer conceptual boundaries and a more explicit explanation of how themes relate to the topic guide and to each other, as overlapping constructs now dilute the analytical depth. Additionally, the Discussion should be reorganized to synthesize key findings, avoid repetition, and more clearly distinguish empirically supported insights from theoretical reflections**.

R. We thank the reviewer for the valuable feedback. We have clarified the theoretical distinction between centralized and decentralized VCC designs and ensured consistent integration across the Background, Methods, and Results sections. The thematic structure has been refined to establish clearer conceptual boundaries, and the Discussion section has been reorganized to synthesize key findings, reduce repetition, and distinguish empirical insights from theoretical reflections.

**Q23. Abstract, Strengths: Clearly structured, addressing background, aim, methods, results, and implications. Points for Improvement: Line 32: The types of VCC designs emerging in practice are mentioned, but neither centralized nor decentralized VCCs are clearly defined. The absence of definitions hampers the reader’s ability to interpret the findings throughout the manuscript**. **The Results section should more explicitly summarize the three major analytical themes, rather than focusing predominantly on the two organizational.**

R. We have incorporated concise definitions of centralized and decentralized VCCs into the abstract and summarized the three major analytical themes in the Results section of the Abstract.

*C.* *Two distinct organizational designs for VCCs have emerged from the data: decentralized designs, embedded within individual hospitals, and centralized designs, which coordinate care across multiple hospitals at a national level. (p.2, line 40-42)*

*To explore the different characteristics of the two organizational designs, these themes were derived: 1) operational implications, 2) collaboration between healthcare providers, and 3) patient-healthcare provider relationships. (p.2, line 42-44)*

**Q24.** **Line 51: Clarify what the findings imply for the design, implementation, or management of VCCs.**

R. We now more clearly present what the findings imply for design, implementation, and management to enhance relevance for healthcare professionals.

*C. The findings are relevant for policy makers working with VCCs, as setting up a VCC is more complex than it may appear. This study examines decentralized and centralized designs, each with different features and outcomes, that policy makers should consider. (p.2, line 53-56)*

**Q25. Background: The literature review is largely descriptive; engaging with organizational design theory, implementation science, or sociotechnical systems theory would strengthen the conceptual foundation.**

R. Thank you for the suggestion. The conceptual foundation has been strengthened by integrating organizational design theory.

*C. As a result, the designs of VCCs are not evidence-based, although such approaches in organizational design theory have been shown to be important for ensuring patient safety and staff satisfaction [20]. (p. 5, line 108-110)*

Additional reference:

[20] Shoemaker LK, Swanson Kazley A, White A. Making the case for evidence-based design in healthcare: A descriptive case study of organizational decision making. Health Environments Research & Design Journal. 2010;4(1). doi:10.1177/193758671000400105

**Q26: Line 64: Provide a clear definition of VCCs, as no generally accepted definition currently exists and VCC models are different in internationally.**

R. Thank you for the comment. A clear definition of VCCs has been added to the Background section, in line with similar comments by Reviewer 1 and 2, acknowledging international differences and situating the Dutch context within a broader healthcare systems perspective.

*C. RPM uses digital technologies to collect medical and physiological data from patients outside clinical settings and transmit it to health care providers for assessment and guidance [6]. (p.4, line 75-77)*

**Q27. Line 70: The two groups described (chronic disease management vs. early discharge post-surgery) are not conceptually aligned. It is unclear what the authors aim to distinguish here. Later (line 88), the manuscript distinguishes between critical and non-critical pathways—please reconcile these categorizations.**

R. Thank you for the comment. The examples of monitoring types have been revised to reflect perioperative, postoperative, episodic, and chronic monitoring, which captures the diversity of VCC services in the Netherlands while noting differences internationally. The earlier distinction between critical and non-critical pathways has been removed to avoid confusion.

*C. VCCs facilitate a mix of digital and physical care, - for perioperative, postoperative, episodic, and chronic patients – through RPM. (p.4, line 74-75)*

**Q28. Line 85: The phrase “limited attention” requires further specification. What aspects of VCCs have been studied, and to what extent?**

R. Thank you for this comment. We clarified that previous research on remote patient monitoring has focused primarily on clinical outcomes and technology adoption, with no attention given to the organizational design of VCCs. This strengthens the rationale for the study and highlights its theoretical contribution. Moreover, the limited attention with respect to VCCs has been further addressed in Q29.

*C.* *Despite these findings, existing literature conceptualizes RPM as a technological intervention, overlooking the importance of the organizational design in its implementation. (p.4, line 94-96)*

**Q29. Lines 86–88: The novelty of the study should be stated more explicitly. What does this research add beyond earlier conceptualizations of “independent wards”? Clarification is needed.**

R. We explicitly state the novelty of the study in the Background section in which we elevate the discussion on the organizational design of remote patient monitoring. Furthermore, independent wards have been revised to examples in the literature, internationally, on related models of RPM at the ward level.

*C. However, while VCCs are becoming common practice in hospitals, they remain underrepresented in academic literature. Existing studies have described related models of RPM at the ward level, such as clinical management centers in Portugal [15], telehealth centers in Australia [16], virtual wards in the United Kingdom [17], virtual clinics in the United States [18], and medical command centers in Sweden [19]. (p.4-5, line 98-102)*

*Hence, this research provides the first empirical analysis of organizational designs of VCCs in the Netherlands, addressing a gap in the literature where prior work has only presented RPM at the ward level. (p.5, line 110-112)*

**3. Methods**
**Q30: Line 105: The referenced URL is not functional; please correct or replace it.**

R. The referenced URL has been verified and remains functional. We have ensured that the link is correctly formatted and accessible for readers.

*Link:* <https://www.igj.nl/publicaties/publicaties/2022/02/28/factsheet-telemonitoring-bij-volwassenen-thuis> (p. 27, line 728-729)

**Q31. Line 108: Clarify how “suitability” relates to the criteria from reference 21. The rationale for selecting three out of ten VCCs appears pragmatic rather than conceptual; this should be explicitly explained.**

R. We have elaborated on how suitability relates to the criteria from reference 21(we changed the sequence of reference 21 and Table 1) and clarified the selection of the three included VCCs accordingly.

*C.* *Three VCCs were included in our study – A, B, and C – based on two criteria. The first criterion was the number of patients receiving digital care, indicating the scope. The second criterion concerned the range of care pathways provided, defined as structured care plans that integrate clinical interventions with supportive services for the management of specific patient populations [23] (Table 1). (p.5, line 128-131)*

**Q32. Table 1: Specify the time frame for the patient numbers. Include information on the number of full-time equivalents (FTEs) employed in each VCC.**

R. Table 1 has been updated to specify the time frame for patient numbers and now includes the number of full-time equivalents (FTEs) employed in each VCC. These additions provide greater transparency regarding organizational capacity and eliminate any ambiguity present in the previous version, while maintaining consistency with the original data.

*C.* *FTE working in VCC (n) (p.6, Table 1)*

**Q33. Provide information on who conducted the interviews, their professional backgrounds, and how positionality or potential bias was addressed.**

R. We present information on who conducted the interviews, their professional backgrounds, and how positionality and how potential bias was addressed. In S1 File, we present The Consolidated Criteria for Reporting Qualitative Studies (COREQ).

*C.* *This exploratory, qualitative study has been conducted following the consolidated criteria for reporting qualitative research (COREQ) (S1 File) [21]. (p.5, line 120-122)*

**Q34. Describe how coding consistency was ensured (e.g., double coding, consensus meetings, peer debriefing) and by whom.**

R. We now clarify that coding consistency was ensured by peer meetings, which were conducted by all members of the research team to maintain reliability and consistency in our thematic analysis.

*C. Coding consistency was ensured by using peer meetings. (p.7, line 175)*

**Q35: Clarify whether the categorizations of centralized and decentralized VCCs were predefined or emerged inductively during analysis, as this currently appears inconsistent across the manuscript.**

R. In line with comments by Reviewer 1 (Q8), we now explicitly state that categorizations of centralized and decentralized VCCs emerged inductively during analysis but were informed by organizational design theory, reconciling any perceived inconsistency.

*C. Two distinct organizational designs for VCCs have emerged from the data: decentralized designs, embedded within individual hospitals, and centralized designs, which coordinate care across multiple hospitals at a national level. (p.2, line 39-42)*

*Two distinct organizational VCC designs emerged from the data: decentralized and centralized designs. (p.9, line 189-190)*

**Q36. Explain how the interview topic guide was informed by existing literature and how it aligns with the study aim. This is currently unclear.**

R. The interview topic guide was developed based on organizational design theory and aligned with the study aim. This explanation is added to the Methods section.

*C. An interview topic guide based on scientific literature on organizational design theory [20, 25-27]. (p.7, line 157-159)*

**Q37. Explain how secondary data (e.g., policy documents) were analyzed.**

R. We added more detail on how secondary data, such as policy documents, were analyzed to complement interview findings and strengthen data triangulation.

*C. This data was used to support a more in-depth interpretation of the findings. (p.7, line 165-166)*

**Q38: Table 3 presents results rather than methods and should be moved accordingly. Including an appendix with a coding tree would enhance analytic transparency.**

R. We apologize for the confusion. The sentence referring to Table 3 heading has been revised to improve clarity.

*C.* *An example of the coding process for theme 2 is presented in Table 3. (p.8, line 182-183)*

*Table 3. Example of the development of theme 2 based on our thematic analysis. (p.8, line 185)*

**4. Results**
**Q39. It remains unclear how the centralized and decentralized designs informed sampling, data collection, and analysis. The manuscript suggests these models emerged from the data, but they also appear to structure the study a priori. This requires clarification.**

R. We thank the reviewer for pointing this out. Centralized and decentralized designs were not predetermined but emerged inductively from the data, while being informed by organizational design theory. This explanation is now explicitly stated in both the Methods and Results sections to resolve any ambiguity regarding the analytical approach.

*C. Two distinct organizational designs for VCCs have emerged from the data: decentralized designs, embedded within individual hospitals, and centralized designs, which coordinate care across multiple hospitals at a national level. (p.2, line 39-42)*

*Two distinct organizational VCC designs have emerged from the data: decentralized and centralized designs. (p.8, line 189-190)*

**Q40. There is conceptual overlap between Collaboration between healthcare providers and Patient–provider relationships, and between System integration and Continuity of care (lines 242–261). Cross-referencing or re-clustering would improve thematic clarity.**

R. Thank you for this observation. While the themes address distinct aspects, namely ‘collaboration among healthcare providers’ versus ‘relational continuity of care’, we agree that clarity could be improved. We have therefore renamed the theme ‘Continuity of care’ to ‘Relational continuity of care’.

*C: 3.4 Relational continuity of care (p.18, line 460)*

*In decentralized VCCs, relational continuity of care is expected to be more easily achieved by keeping handover of care within the regional network, ensuring that patients receive care from the same team of healthcare providers who are familiar with the patient’s medical history and needs. (p.18, line 461)*

*The medical specialists and nurses are delegated from multiple hospitals, which makes it more challenging to preserve the personal relationships and therapeutic alliances that are essential for ensuring relational continuity of care. (p.18, line 469)*

**Q41. Lack of explicit definitions (Table 4): Each theme (e.g., Operational framework, Personal contact) would benefit from concise conceptual definitions. • Clarify what is meant by Theme 1: Operational framework. Why is this considered a “framework”? • Theme 1 is broad and combines conceptually distinct elements (monitoring workflows, SOPs, system integration). Consider refining or differentiating subthemes.**

R. We have revised Theme 1 by replacing ‘Operational framework’ with ‘Operational implications’ to better reflect its content. In addition, each major theme now includes a concise conceptual definition directly under the Results section, and subthemes have been refined to differentiate monitoring workflows, SOPs, and system integration. These changes improve thematic clarity and eliminate ambiguity.

*C. These two organizational VCC designs differ in three themes. The first theme are the operational implications, referring to the organizational processes, workflow structures, and system integration. The second theme constitutes the collaboration between healthcare providers, while the third theme incorporates the patient–healthcare provider relationships. The themes and their corresponding subthemes are identified in Table 4. (p.9, line 196-201)*

**Q42. The Results contain highly operational descriptions (e.g., alert distribution, filtering processes). Consider foregrounding structural or organizational mechanisms over workflow details.**

R. We thank the reviewer for this comment. We retained the operational descriptions (e.g., alert distribution and filtering processes) because they demonstrate how the organizational design is enacted in practice, which is essential for understanding the resulting workflow implications. Furthermore, as the existing literature provides lacks insight into the organizational designs for VCCs, these descriptions add value by illustrating how operational processes unfold within each design.

**Q43. SOP-related content overlaps with monitoring workflows. Refining or integrating these subthemes may improve coherence.**

R: We thank the reviewer for this comment. We acknowledge that monitoring workflows could, in principle, be grouped under SOP-related content. However, we kept these subthemes separate because SOPs capture the overarching, formalized ways of working, whereas workflows describe the more dynamic, process‑level operational activities. Merging them would obscure this distinction and reduce conceptual clarity.

**Q44. System integration (lines 244–261): The description conflates EHR interoperability with device and communication system integration. Analytically separating these elements would improve clarity.**

*R.* We thank the reviewer for this comment and we apologize for the confusion. We replaced system integration with system interoperability, as these concepts involve different technical and organizational challenges, and our results specifically address interoperability. Accordingly, we updated Heading 1.3.

*C. 1.3 System interoperability and data accessibility (p.12, line 278)*

**Q45. Lines 256–261 emphasize the importance of complete patient information, which overlaps with Continuity of care. Consider cross-referencing or integrating this into Theme 4.**

R. We thank the reviewer for this comment. We clarified the terminology and specified that the theme concerns relational continuity of care (see also adjustments made for Q40). Because relational continuity focuses on patients receiving care from the same team of providers, it differs conceptually from the emphasis on complete patient information, which relates to the technical availability and accessibility of data. For this reason, cross‑referencing or merging the two would conflate distinct constructs.

**Q46. Subthemes such as Personal contact between colleagues and Trust in colleagues also relate to relational dynamics, which partially overlap with Theme 3 (patient-facing). Consider reorganizing these relationships for conceptual consistency.**

R. Thank you for this suggestion. Subthemes like personal contact and trust in colleagues were maintained under interprofessional collaboration rather than patient-facing themes, as they pertain to professional relationships within the care team. These clarifications preserve conceptual integrity and ensure that each theme captures a unique dimension of VCC design.

**Q47. The findings on trust require clarification: Table 4 suggests both reduced trust and trust strengthened by formal agreements in centralized designs. Distinguish these dynamics more clearly.**

R. Thank you for this observation. We agree that the previous explanation of trust was not fully aligned with our results. We have revised the text in Table 4.

*C. Trust may become more challenging to establish. (p.10, Table 4)*

**5. Discussion**
**Q48. The Discussion lacks an initial summary of the key findings; adding this would help orient the reader.**

R. Thank you for pointing this out. We have added a concise summary of the key findings at the beginning of the Discussion to better orient the reader.

*C. This study examined the organizational designs of VCCs in the Netherlands and identified a centralized and decentralized design. The distinction between these two designs is not rigid, hybrid forms may emerge that incorporate elements of both. The findings were categorized into three themes: 1) operational implications, 2) collaboration between healthcare providers, and 3) patient-healthcare provider relationship, which appeared across both designs. (p.19, line 476-480)*

**Q49. The Discussion largely restates results and does not sufficiently integrate themes or discuss how themes relate to each other. Some arguments are not clearly grounded in the findings.**

R. We thank the reviewer for this comment. We have clarified in the Discussion that all three themes emerged across both the centralized and decentralized VCC designs. While the themes collectively characterize core elements of VCC functioning, their interconnections are not linear or strongly interdependent, which is why they were not merged. In response to the reviewer’s suggestion, we strengthened the integration of themes by expanding the discussion of operational implications, particularly regarding system interoperability and data accessibility (see response to Q21).

*C. The findings were categorized into three themes: 1) operational implications, 2) collaboration between healthcare providers, and 3) patient-healthcare provider relationship, which appeared across both designs. (p.19, line 478-480)*

**Q50. Comparisons with prior studies should more explicitly relate to your own findings and clarify whether cited literature concerns VCCs specifically or digital health more broadly. Also, VCC models from other countries are not discussed at all.** 

R. We thank the reviewer for pointing this out. We clarified the contribution to the existing literature and its interdisciplinary nature (Q12). As the existing literature does not examine organizational designs of VCCs, comparisons necessarily draw on studies addressing underlying concepts, such as coordination, digital workflows, and patient interaction, within digital health more broadly. Regarding international VCCs, we clarified in the Introduction that although the study acknowledges the existence of VCC models in other countries, its focus is explicitly limited to the Netherlands (Q2). We have updated the Discussion to make this distinction explicit.

*C. This study addresses a research gap in the literature, as organizational designs of VCCs have not previously been empirically examined beyond RPM at the ward level [15-19]. Our study bridges operations management and digital health disciplines, as the VCC designs possess elements related to processes and coordination, both between patients and healthcare professionals. The discussion positions these findings within the broader organizational design literature, showing how they align with and extend existing concepts. (p.19, line 481-486)*

**Q51. Line 453: The argument here seems to contradict Theme 1, which suggests centralized VCCs may offer more personalized care. This tension should be resolved.** 

R. We apologize for the confusion as we aimed to center our argumentation around the challenges related to the delivery of patient-centered care, rather than personalization. We changed the sentences and, as such, resolved the tension that arose.

*C.* *This tension between standardized protocols and patient-centered care appears to reflect structural characteristics of program‑based care pathways more generally, rather than stemming solely from the organizational design of VCCs. (p. 20, line 508-511)*

**Q52. The Discussion states that centralized VCCs may “potentially improve efficiency,” while also claiming that the study does not confirm such improvements. This requires reframing to avoid speculative interpretation.** 

R. We thank the reviewer for this comment. We have removed the statement suggesting that centralized VCCs may “potentially improve efficiency,” as the study does not provide empirical evidence to support this a statement. In line with the reviewer’s feedback and our response to Q4, we now frame this point as a direction for future research.

*C. Moreover, subsequent studies should examine how organizational design choices impact cost-effectiveness and operational efficiency, broadening the understanding of how organizational structures influence RPM outcomes. (p.23, line 625-628)*

**Q53.The argument that challenges stem primarily from program-based care pathways rather than organizational design is promising but underdeveloped; further theoretical grounding is recommended.** 

R. Thank you for pointing this out. We have expanded this section by adding theoretical approach related to fragmented care. The revised text now provides deeper theoretical grounding and strengthens the argument.

*C. This challenge is not unique to VCCs but aligns with broader concerns about fragmented care, in which chronic patients receive care from multiple providers and settings that do not consistently communicate or coordinate with one another [34]. This tension between standardized protocols and patient-centered care appears to reflect structural characteristics of program‑based care pathways more generally, rather than stemming solely from the organizational design of VCCs. (p.20, line 508-513)*

Additional reference:

[34] Joo, J. Y. (2023). Fragmented care and chronic illness patient outcomes: A systematic review. Nursing Open, 10, 3460–3473. https://doi.org/10.1002/nop2.1607

**Q54. The difficulty in aligning regional policies is noted but not explained in terms of implications for national governance, resource allocation, or scaling.**

R. We thank the reviewer for this insightful comment and apologize for the lack of clarity. Although aligning regional policies could theoretically offer advantages for centralized VCCs, this alignment remains difficult in practice. Persistent differences between VCCs in hospitals across the Netherlands both in organizational structures and operational priorities make harmonization challenging. As a result, the implications for national governance, resource allocation, and scaling remain limited, as regional variation constrains the ability to implement uniform approaches.

*C. However, maintaining alignment with the diverse regional policies across the Netherlands remains a significant challenge for these centralized designs. (p.19, line 495-496)*

**Q55. The claim that geographical separation hinders collaboration requires nuance; digital and regional teamwork literature shows that physical distance does not necessarily impede effective collaboration.**

R. We thank the reviewer for this valuable comment. We have nuanced our argument by clarifying that the challenge identified in our study does not relate to geographical distance per se. Instead, drawing on our empirical findings, we explain that the difficulties arise from organizational distance, specifically, limited prior relationships and weak relational ties between partnering hospitals within centralized VCCs. We have updated the manuscript accordingly to reflect this distinction.

*C. Although physical distance alone does not inherently prevent effective collaboration, our empirical results indicate that the challenges observed in centralized VCCs arise from organizational rather than geographical distance. In centralized VCCs, collaboration often involves hospitals that are more institutionally remote, partners with whom staff have limited prior contact or established working relationships. This highlights the importance of relational governance structures. Specifically, the empirical findings indicate that centralized VCCs require not only the development of trust and effective communication between organizations but also the presence of formalized inter‑organizational governance arrangements [36]. The literature further notes that, alongside relational governance, these inter-organizational structures also include contractual mechanisms [37] to ensure that various arrangements, such as finances and data management [38], are properly managed and safeguard the involved parties against potential opportunistic behaviors [39]. (p.20/p.21, line 532-540)*

**Q56. Relational and contractual governance structures are introduced, but links to empirical findings are not always clear. Please specify what is based on data vs. literature.** 

R. We thank the reviewer for this helpful comment. We clarified the distinction between what is based on theoretical and empirical findings.

*C. Specifically, the empirical findings indicate that centralized VCCs require not only the development of trust and effective communication between organizations but also the presence of formalized inter‑organizational governance arrangements [36]. The literature further notes that, alongside relational governance, these inter-organizational structures also include contractual mechanisms [37] to ensure that various arrangements, such as finances and data management [38], are properly managed and safeguard the involved parties against potential opportunistic behaviors [39]. (p.21, line 538-540)*

**Q57. The “threshold” at which decentralized VCCs may shift toward more centralized designs is a theoretical proposition; emphasize that this was not empirically established in the current study.**

R. Thank you for pointing this out. We clarified that this was not empirically established and is a theoretical proposition.

*C. From a theoretical standpoint, the strong relational foundation in decentralized VCCs may support more effective integration and collaboration with regional healthcare providers. However, a decentralized VCCs may face limitations in regional expansion. As the patient population increases, the personal contact that characterizes decentralized designs may become unsustainable. This theoretical constraint invites consideration of a scale threshold at which a decentralized VCC would functionally transition toward a centralized design. Identifying this ‘cut-off’ point is essential for ensuring that effective collaboration and integration are preserved during scaling-up efforts. Future research should empirically examine this ‘cut-off’ point. (p.21, line 547-555)*

**Q58. The question of whether centralized VCCs deliver faster assistance should be clarified: does this concern arise from participant accounts or conceptual critique?**

R. Thank you for this comment. We clarified that the concern arises from the participant accounts, specifically from the respondents.

*C. While centralized designs have been hailed for their ability to increase the pace of assistance based on the respondents, it is questionable whether this design actually enables quicker assistance. (p. 22, line 573-575)*

**Q59. The Discussion mentions that literature is inconclusive about power-sharing in VCCs, but cited studies address telemonitoring more broadly. Clarify this distinction.** 

R. Thank you for pointing this out. We clarified that the cited literature stems from telemonitoring and remote patient monitoring (RPM) more broadly, rather than VCCs specifically.

*C. Previous research on RPM and telemonitoring has provided evidence for higher levels of sharing power and responsibility because of RPM [43], where patients are active participants in their treatment decisions [43]. At the same time, other research emphasized that patient empowerment is not improved by digital care provision [45]. (p.22, line 585-589)*

**Q60. The text alternates between explanations based on patient empowerment and selection bias; these concepts should be disentangled and tied explicitly to empirical findings**. 

R. We thank the reviewer for this valuable comment. We have clarified that the discussion of patient empowerment and selection bias is grounded in our empirical findings and have explicitly disentangled these concepts in the revised text.

*C. However, respondents also emphasized that patients must meet certain eligibility criteria to be enrolled in RPM programs.* *Hence, the empirical findings show that this introduces a potential selection bias, as it is difficult to determine whether the observed increase in autonomy stems from the organizational design of VCCs or from the pre-existing self-management capacities of patients who are deemed suitable for remote monitoring. This ambiguity aligns with existing literature that reports inconsistent empirical evidence regarding whether RPM genuinely redistributes power and responsibility between patients and healthcare providers. (p. 22, line 591-598)*

**Q61. Claims about threats to relational continuity, particularly in centralized VCCs, should be directly linked to the findings**.

R. Thank you for this comment. We replaced the general term continuity of care with relational continuity of care (see our response to Q40) to ensure conceptual precision. The discussion now explicitly refers to the relevant findings, where the underlying mechanisms of this issue are described in more detail.

**Q62. Consider suggesting how relational continuity could be improved within centralized VCCs.** 

R. Thank you for this suggestion. Neither our empirical findings nor the existing literature provides a basis for formulating grounded recommendations on how relational continuity of care could be improved within centralized VCCs. As a result, we are unable to propose evidence‑based strategies at this stage. We, therefore, highlight this as an important challenge to be addressed and examined in future research.

**6. Limitations and Future Research**
**Q63: The first limitation states that patient perspectives were not captured, but this was not an aim of the study; clarify the relevance of this limitation. Also note that only one theme could be validated with patient perspectives.**

R: Thank you for this comment. We agree that patient perspectives were not an aim of the study and removed this as a limitation to avoid confusion.

**Q64. Clarify how professional role differences (lines 544–546) may have shaped the findings—for example, nurses and medical specialists may have different views on collaboration.**

R. We thank the reviewer for this valuable comment. We have expanded the limitations section to acknowledge that differences in professional roles among respondents may have influenced the findings. These role-specific perspectives could have shaped the findings and should therefore be considered a limitation of the study.

*C. Furthermore, differences in respondents’ professional roles may have influenced their perspectives and therefore limit the generalizability of the findings. (p.23, line 623-628)*

**Q65. While the limitation of including only 3 out of 10 VCCs is acknowledged, its implications for transferability and contextual variation should be explained. Access challenges should also be described in the Methods.**

R: Thank you for this comment. We have expanded the limitation regarding the inclusion of only three out of ten Dutch VCCs. The revised text explains that this restricts transferability and may not capture the full contextual variation across all VCCs. We also note that access challenges influenced sampling decisions, and this has now been described in the Methods section for transparency. Future studies should include a broader sample to strengthen generalizability and examine diverse organizational configurations.

*C. Using purposive sampling, ten VCCs in the Netherlands employing RPM were identified [22]. Three VCCs were included in our study – A, B, and C – based on two criteria. The first criterion was the numbers of patients receiving digital care, indicating the scope. The second criterion concerned the range of care pathways provided, defined as structured care plans that integrate clinical interventions with supportive services for the management of specific patient populations [23] (Table 1). (p.5, line 126-131)*

*First, this study included only three out of ten VCCs in the Netherlands, potentially limiting the generalizability of our results. Future research should examine more VCC designs. (p.23, line 617-622)*

**Q66. The absence of hybrid VCC models is noted but not introduced earlier in the manuscript; consider discussing or defining hybrid models earlier.**

R. We thank the reviewer for this comment. Indeed, we should mention this earlier on and have therefore extended this in the findings results in the beginning of the Discussion, because the organizational designs centralized and decentralized emerged as the two types, but acknowledging potential hybrid forms.

*C. The distinction between these two designs is not rigid, hybrid forms may emerge that incorporate elements of both. (p.19, line 477-478)*
