# Supplementary material for: The organization of virtual care centers: A qualitative study in Dutch hospitals
Source: PLOS Digit Health. 2026 Jun 26;5(6):e0001479. doi: 10.1371/journal.pdig.0001479 (PMC13308795; doi:10.1371/journal.pdig.0001479)
Supplement: Peer review history

# Peer review: Decision Letter 0

**DOI:** 10.1371/journal.pdig.0001479.r001

**Authors:** Laura Maaß, Zhao Ni

23 Nov 2025

PDIG-D-25-00451

The organization of virtual care centers: A qualitative study in Dutch hospitals

PLOS Digital Health

Dear Dr. Peters,

Thank you for submitting your manuscript to PLOS Digital Health. After careful consideration, we feel that it has merit but does not fully meet PLOS Digital Health's publication criteria as it currently stands. Therefore, we invite you to submit a revised version of the manuscript that addresses the points raised during the review process.

Please submit your revised manuscript by Jan 22 2026 11:59PM. If you will need more time than this to complete your revisions, please reply to this message or contact the journal office at digitalhealth@plos.org. When you're ready to submit your revision, log on to https://www.editorialmanager.com/pdig/ and select the 'Submissions Needing Revision' folder to locate your manuscript file.

Please include the following items when submitting your revised manuscript:

* A rebuttal letter that responds to each point raised by the editor and reviewer(s). You should upload this letter as a separate file labeled 'Response to Reviewers'. This file does not need to include responses to any formatting updates and technical items listed in the 'Journal Requirements' section below.

* A marked-up copy of your manuscript that highlights changes made to the original version. You should upload this as a separate file labeled 'Revised Manuscript with Track Changes'.

* An unmarked version of your revised paper without tracked changes. You should upload this as a separate file labeled 'Manuscript'.

If you would like to make changes to your financial disclosure, competing interests statement, or data availability statement, please make these updates within the submission form at the time of resubmission. Guidelines for resubmitting your figure files are available below the reviewer comments at the end of this letter.

We look forward to receiving your revised manuscript.

Kind regards,

Laura Maaß, PhD

Academic Editor

PLOS Digital Health

Leo Anthony Celi

Editor-in-Chief

PLOS Digital Health

orcid.org/0000-0001-6712-6626

**Journal Requirements:**

1. Please note that your Data Availability Statement is currently missing the repository name and/or the DOI/accession number of each dataset OR a direct link to access each database. If your manuscript is accepted for publication, you will be asked to provide these details on a very short timeline. We therefore suggest that you provide this information now, though we will not hold up the peer review process if you are unable.

2. Please insert an Ethics Statement at the beginning of your Methods section, under a subheading 'Ethics Statement'. It must include:

1) The name(s) of the Institutional Review Board(s) or Ethics Committee(s)

2) The approval number(s), or a statement that approval was granted by the named board(s)

3) (for human participants/donors) - A statement that formal consent was obtained (must state whether verbal/written) OR the reason consent was not obtained (e.g. anonymity). NOTE: If child participants, the statement must declare that formal consent was obtained from the parent/guardian.

3. Your manuscript is missing the following sections: Introductions. Please ensure these are present, and in the correct order, and that any references to subheadings in your main text are correct. An outline of the required sections can be consulted in our submission guidelines here:

https://journals.plos.org/digitalhealth/s/submission-guidelines#loc-parts-of-a-submission

4. If the reviewer comments include a recommendation to cite specific previously published works, please review and evaluate these publications to determine whether they are relevant and should be cited. There is no requirement to cite these works unless the editor has indicated otherwise.

**Reviewers' Comments:**

Reviewer's Responses to Questions

**Comments to the Author**

1. Does this manuscript meet PLOS Digital Health’s publication criteria? Is the manuscript technically sound, and do the data support the conclusions? The manuscript must describe methodologically and ethically rigorous research with conclusions that are appropriately drawn based on the data presented.

Reviewer #1: Partly

Reviewer #2: Yes

Reviewer #3: Yes

**********

2. Has the statistical analysis been performed appropriately and rigorously?

Reviewer #1: N/A

Reviewer #2: Yes

Reviewer #3: N/A

**********

3. Have the authors made all data underlying the findings in their manuscript fully available (please refer to the Data Availability Statement at the start of the manuscript PDF file)?

The PLOS Data policy requires authors to make all data underlying the findings described in their manuscript fully available without restriction, with rare exception. The data should be provided as part of the manuscript or its supporting information, or deposited to a public repository. For example, in addition to summary statistics, the data points behind means, medians and variance measures should be available. If there are restrictions on publicly sharing data—e.g. participant privacy or use of data from a third party—those must be specified.

Reviewer #1: Yes

Reviewer #2: Yes

Reviewer #3: Yes

**********

4. Is the manuscript presented in an intelligible fashion and written in standard English?

PLOS Digital Health does not copyedit accepted manuscripts, so the language in submitted articles must be clear, correct, and unambiguous. Any typographical or grammatical errors should be corrected at revision, so please note any specific errors here.

Reviewer #1: Yes

Reviewer #2: Yes

Reviewer #3: Yes

**********

5. Review Comments to the Author

Please use the space provided to explain your answers to the questions above. You may also include additional comments for the author, including concerns about dual publication, research ethics, or publication ethics. (Please upload your review as an attachment if it exceeds 20,000 characters)

Reviewer #1: Reviewer Report

General Evaluation

Thank you for the opportunity to review this interesting and timely manuscript examining the organizational designs of virtual care centres (VCCs) in Dutch hospitals. The topic is clearly relevant for PLOS Digital Health and contributes to our understanding of how digital health services are structured and managed in practice.

Overall, the paper is well written and provides valuable empirical insight into a field that remains underexplored. My comments aim to help the authors further clarify some conceptual and methodological aspects and to strengthen the paper’s positioning and coherence. I would recommend minor revisions before the paper can be considered for publication.

Comments

1. Background

1.1 Conceptual clarity – Definition of remote patient monitoring (RPM)

The authors define remote patient monitoring (RPM) as “supervision and guidance of patients through the remote monitoring of vital indicators and symptoms.” However, this definition seems conceptually inconsistent. Monitoring refers to the collection and observation of patient data (e.g., vital signs, symptoms) rather than the direct provision of care or supervision.

In several parts of the manuscript, the authors describe activities such as nurses monitoring alerts, offering support, and enabling remote care delivery. These actions go beyond monitoring and clearly belong to the realm of remote patient care or remote patient management. I would recommend that the authors reconsider the terminology used throughout the paper. If the focus is on clinical engagement and support provided remotely, terms such as remote patient care or remote patient management might be more appropriate. Alternatively, if they wish to keep the RPM terminology, they should explicitly explain that they apply the concept in a broader sense, covering both monitoring and professional interventions.

Furthermore, the cited definition (from 2007) appears outdated given the rapid conceptual and technological evolution of telehealth. More recent definitions could be used to provide a more contemporary framing of the concept. Clarifying this distinction would significantly improve the conceptual rigor of the paper and strengthen its theoretical foundation.

1.2 Definition and contextual scope of “virtual care centres”

The authors define virtual care centres as facilities focusing on postoperative monitoring at home and long-term disease management. While this apparently reflects the current use of the term in the Dutch healthcare context, it is important to note that the concept of virtual care centres varies internationally.

In many other countries (for example Finland, Canada, and the UK), virtual care centres or virtual health centres typically provide a much broader range of primary and preventive care services, including medication renewals, symptom-based triage, remote consultations, and self-care guidance. To enhance conceptual clarity and international relevance, I recommend that the authors explicitly state that their definition applies to the Dutch healthcare setting, and briefly acknowledge that alternative virtual care centre models exist globally. This clarification would help readers interpret the study’s findings within the appropriate national and organizational context.

1.3 Organizational perspective on RPM implementation

The authors rightly point out that previous studies have predominantly treated remote patient monitoring (RPM) as a technological intervention, often overlooking the importance of organizational design in its implementation. This is an important and valuable observation that adds theoretical depth to the study.

In organizational and health management literature, it is well established that different organizational configurations—such as centralized or decentralized models—can substantially influence the outcomes and efficiency of technology-enabled care delivery. These configurations affect not only workflow and coordination, but also resource allocation, scalability, and ultimately the economic sustainability of such interventions.

Although this paper does not address economic outcomes directly, it would be worthwhile to discuss how the chosen organizational design impacts cost-effectiveness and operational efficiency. Including this perspective in subsequent studies could broaden the understanding of how organizational structures shape the realized benefits of RPM.

1.4 Study aim and alignment with the stated research gap

The statement of the study’s aim appears inconsistent across sections. In the Background, the authors write that the aim is “to examine organizational designs of VCCs in Dutch hospitals,” whereas the Abstract refers to exploring and characterizing their design to provide a clearer understanding of their structure and implications. In the Author Summary, the authors claim to address a gap concerning the lack of empirical evidence on organizational differences and practical implications of VCCs.

These formulations are related but not fully equivalent. The first (“to examine organizational designs”) describes a rather descriptive purpose, while the latter two imply an analysis of implications and consequences of organizational variation. As currently formulated, it is not entirely clear whether the presented aim allows the authors to address the stated research gap.

I would recommend harmonizing the aim statement across all sections and clarifying whether the study intends merely to describe existing organizational models or also to analyze their practical implications. If the latter is intended, the aim could be expanded accordingly. This clarification would strengthen the internal consistency and conceptual coherence of the paper.

2. Methods

2.1 Selection of organizations and sampling rationale

The study includes three virtual care centres (VCCs) selected from a total of ten operating in the Netherlands. The authors note that the selection was based on purposeful sampling, considering the number of patients and care pathways. However, the rationale for including these specific three centres—and excluding the remaining seven—is not fully explained.

The current description of the sampling procedure does not provide sufficient clarity or transparency to understand why these organizations were chosen. Readers are left unsure whether the selection reflects diversity in organizational models, data accessibility, or other contextual factors. Providing a more explicit explanation of how and why these organizations were selected from the ten possible ones would strengthen the methodological transparency and help readers assess the robustness of the findings.

I recommend that the authors elaborate on the selection criteria and briefly indicate how the chosen organizations relate to the broader landscape of Dutch VCCs. This clarification would improve the study’s credibility and interpretability.

2.2 Scope, duration, and validation of interviews

The manuscript states (S2 fine) that seven broad thematic areas were covered in the interviews, each consisting of multiple sub-questions, and that each interview lasted approximately 30 minutes. While the authors mention that participants were given the opportunity to review their transcribed interviews—and that four participants made minor corrections—this process primarily ensures transcriptional accuracy rather than substantive expansion or deepening of the discussion.

Given the complexity of the topic, a 30-minute timeframe appears rather limited for exploring multiple themes in sufficient depth. Although this issue cannot be remedied retrospectively, it may be worth acknowledging as a limitation. The authors might briefly note that time constraints and the professional context of the participants could have restricted opportunities for reflection and elaboration, which is a common challenge in qualitative research conducted in clinical environments. Including such a note would demonstrate methodological reflexivity and transparency.

2.3 Emergence of organizational models in the analysis

The authors state that the analysis led to the identification of two distinct organizational designs—centralized and decentralized. However, it remains somewhat unclear whether this distinction truly emerged from the analytical process or whether it was already evident from the selection of organizations.

Given that the included VCCs differed substantially in scale (e.g., 600 vs. 23,000 patients) and operational scope (local vs. national), readers may wonder whether the centralized and decentralized models were at least partially known or anticipated prior to the analysis. If this was the case, the analysis may have confirmed or elaborated on an existing assumption rather than generating the distinction inductively.

I would suggest clarifying whether the identification of these two organizational models was an outcome of the data analysis or whether it represents a theoretically informed categorization established earlier in the research design. This clarification would enhance the transparency and interpretive rigor of the methodological account.

3. Discussion

3.1 Clarity and consistency of the study’s implications

The term implications is used in several parts of the manuscript—in the Author Summary (“practical implications”), in the Data Analysis section (“specific implications”), and again in the Discussion (“operational implications”). However, it remains somewhat unclear what type or level of implications the study ultimately aims to produce. Are these implications primarily operational, practical, managerial, organizational, or theoretical in nature?

This ambiguity also relates to the earlier concern about the study’s stated aim: if the primary contribution is to clarify the organizational designs of VCCs, the authors should specify what implications arise from this clarification and for whom (e.g., healthcare managers, clinicians, policymakers).

I recommend that the authors more explicitly articulate the main implications in the Discussion and summarize them clearly in the Conclusion. A short synthesis highlighting what the study contributes in terms of actionable or conceptual insights would strengthen the paper’s coherence and readability.

4. Limitations and Future Research

4.1 Reflection on selection bias in the Limitations section

The authors appropriately acknowledge a potential selection bias related to the recruitment of interview participants by department heads. It might also be valuable to extend this reflection to the earlier stage of organizational selection. As noted previously, only three out of ten Dutch VCCs were included in the study, but the rationale for their selection remains somewhat unclear.

A brief acknowledgement that selection bias could also apply to the organizational level—depending on how the participating centres were identified and approached—would further strengthen the transparency of the Limitations section. This addition would show awareness of potential sources of bias across multiple levels of the research process.

5. Overall Reflection on Disciplinary Positioning

5.1 Positioning and value of interdisciplinarity

The study clearly integrates elements from several academic domains, including organizational and management studies, operations management, nursing science, and digital health technology research. This interdisciplinary nature is one of the paper’s strengths and reflects the multifaceted reality of virtual care delivery.

However, it may be helpful for readers if the authors could more explicitly articulate how they position their study within a particular scientific conversation or discipline. At present, the discussion remains somewhat general, and the reference list does not show a clear concentration in any specific field. Clarifying the disciplinary lens could strengthen the conceptual anchoring and the perceived scholarly contribution.

At the same time, the interdisciplinary scope of the work adds important value: it enables a more holistic understanding of a complex socio-technical phenomenon, bridges theoretical and practical perspectives, and fosters dialogue between disciplines that are often studied in isolation. Briefly highlighting this integrative contribution, perhaps in the Discussion or Conclusion, would underline both the novelty and the broader relevance of the paper.

Final Recommendation

This is a promising and well-executed manuscript that makes a relevant contribution by addressing organizational design aspects of virtual care centres. With minor revisions focusing on conceptual clarity, methodological transparency, and explicit positioning, the paper will be well suited for publication in PLOS Digital Health.

Recommendation: Accept with minor revisions.

Reviewer #2: I would like to sincerely thank the editors for the opportunity to review this manuscript on the Organization of Virtual Care Centers. The paper represents a high-quality scientific contribution that addresses a topic of both substantial practical importance and international academic relevance. The authors provide a well-structured and comprehensive discussion of organizational models, governance, and care delivery processes within Virtual Care Centers, offering valuable insights for the advancement of modern healthcare systems.

The selection of this journal as the publication venue is, in my opinion, highly appropriate. The paper fits well within its thematic scope and will be of great interest to a broad readership in health services research, digital health policy, and clinical innovation.

From a holistic perspective, the manuscript is scientifically sound, methodologically robust, and clearly written. However, one important aspect deserves further consideration: the data infrastructure that underpins telemonitoring and digital care processes. This element is mentioned only briefly, though it constitutes a fundamental and enabling pillar for any sustainable and scalable Virtual Care Center. For future refinement of the paper, I would strongly encourage the authors to elaborate on this dimension and to discuss its role in ensuring interoperability, data quality, and secure information exchange.

Moreover, it may be worthwhile to explicitly include — in the conclusion section — a statement advocating for the establishment of interoperable data infrastructures as a prerequisite for the further development of telemonitoring and integrated virtual care.

Further detailed comments and specific suggestions can be found in the attached annotated PDF.

Reviewer #3: General

This manuscript presents a well-written and timely qualitative study examining the organizational designs of Virtual Care Centers (VCCs) in Dutch hospitals. The topic is highly relevant given the rapid digitalization of healthcare delivery. The authors identify and compare two distinct organizational models, centralized and decentralized, offering valuable empirical insights into how these designs affect collaboration and patient- provider relationships. Overall, the paper is clear and logically structured but several substantive improvements are needed to strengthen its conceptual clarity and analytical coherence. Most importantly, the distinction between centralized and decentralized VCC designs requires clearer theoretical definition and consistent integration throughout the Background, Methods, and Results, as it currently appears both predetermined and emergent. The thematic structure would benefit from clearer conceptual boundaries and a more explicit explanation of how themes relate to the topic guide and to each other, as overlapping constructs now dilute the analytical depth. Additionally, the Discussion should be reorganized to synthesize key findings, avoid repetition, and more clearly distinguish empirically supported insights from theoretical reflections.

1. Abstract

Strengths

• Clearly structured, addressing background, aim, methods, results, and implications.

Points for Improvement

• Line 32: The types of VCC designs emerging in practice are mentioned, but neither centralized nor decentralized VCCs are clearly defined. The absence of definitions hampers the reader’s ability to interpret the findings throughout the manuscript.

• The Results section should more explicitly summarize the three major analytical themes, rather than focusing predominantly on the two organizational models.

• Line 51: Clarify what the findings imply for the design, implementation, or management of VCCs.

2. Background

• The literature review is largely descriptive; engaging with organizational design theory, implementation science, or sociotechnical systems theory would strengthen the conceptual foundation.

• Line 64: Provide a clear definition of VCCs, as no generally accepted definition currently exists and VCC models are different in internationally.

• Line 70: The two groups described (chronic disease management vs. early discharge post-surgery) are not conceptually aligned. It is unclear what the authors aim to distinguish here. Later (line 88), the manuscript distinguishes between critical and non-critical pathways—please reconcile these categorizations.

• Line 85: The phrase “limited attention” requires further specification. What aspects of VCCs have been studied, and to what extent?

• Lines 86–88: The novelty of the study should be stated more explicitly. What does this research add beyond earlier conceptualizations of “independent wards”? Clarification is needed.

3. Methods

• Line 105: The referenced URL is not functional; please correct or replace it.

• Line 108: Clarify how “suitability” relates to the criteria from reference 21. The rationale for selecting three out of ten VCCs appears pragmatic rather than conceptual; this should be explicitly explained.

• Table 1: Specify the time frame for the patient numbers. Include information on the number of full-time equivalents (FTEs) employed in each VCC.

• Provide information on who conducted the interviews, their professional backgrounds, and how positionality or potential bias was addressed.

• Describe how coding consistency was ensured (e.g., double coding, consensus meetings, peer debriefing) and by whom.

• Clarify whether the categorizations of centralized and decentralized VCCs were predefined or emerged inductively during analysis, as this currently appears inconsistent across the manuscript.

• Explain how the interview topic guide was informed by existing literature and how it aligns with the study aim. This is currently unclear.

• Explain how secondary data (e.g., policy documents) were analyzed.

• Table 3 presents results rather than methods and should be moved accordingly. Including an appendix with a coding tree would enhance analytic transparency.

4. Results

• It remains unclear how the centralized and decentralized designs informed sampling, data collection, and analysis. The manuscript suggests these models emerged from the data, but they also appear to structure the study a priori. This requires clarification.

• There is conceptual overlap between Collaboration between healthcare providers and Patient–provider relationships, and between System integration and Continuity of care (lines 242–261). Cross-referencing or re-clustering would improve thematic clarity.

• Lack of explicit definitions (Table 4): Each theme (e.g., Operational framework, Personal contact) would benefit from concise conceptual definitions.

• Clarify what is meant by Theme 1: Operational framework. Why is this considered a “framework”?

• Theme 1 is broad and combines conceptually distinct elements (monitoring workflows, SOPs, system integration). Consider refining or differentiating subthemes.

• The Results contain highly operational descriptions (e.g., alert distribution, filtering processes). Consider foregrounding structural or organizational mechanisms over workflow details.

• SOP-related content overlaps with monitoring workflows. Refining or integrating these subthemes may improve coherence.

• System integration (lines 244–261): The description conflates EHR interoperability with device and communication system integration. Analytically separating these elements would improve clarity.

• Lines 256–261 emphasize the importance of complete patient information, which overlaps with Continuity of care. Consider cross-referencing or integrating this into Theme 4.

• Subthemes such as Personal contact between colleagues and Trust in colleagues also relate to relational dynamics, which partially overlap with Theme 3 (patient-facing). Consider reorganizing these relationships for conceptual consistency.

• The findings on trust require clarification: Table 4 suggests both reduced trust and trust strengthened by formal agreements in centralized designs. Distinguish these dynamics more clearly.

5. Discussion

• The Discussion lacks an initial summary of the key findings; adding this would help orient the reader.

• The Discussion largely restates results and does not sufficiently integrate themes or discuss how themes relate to each other. Some arguments are not clearly grounded in the findings.

• Comparisons with prior studies should more explicitly relate to your own findings and clarify whether cited literature concerns VCCs specifically or digital health more broadly. Also, VCC models from other countries are not discussed at all.

• Line 453: The argument here seems to contradict Theme 1, which suggests centralized VCCs may offer more personalized care. This tension should be resolved.

• The Discussion states that centralized VCCs may “potentially improve efficiency,” while also claiming that the study does not confirm such improvements. This requires reframing to avoid speculative interpretation.

• The argument that challenges stem primarily from program-based care pathways rather than organizational design is promising but underdeveloped; further theoretical grounding is recommended.

• The difficulty in aligning regional policies is noted but not explained in terms of implications for national governance, resource allocation, or scaling.

• The claim that geographical separation hinders collaboration requires nuance; digital and regional teamwork literature shows that physical distance does not necessarily impede effective collaboration.

• Relational and contractual governance structures are introduced, but links to empirical findings are not always clear. Please specify what is based on data vs. literature.

• The “threshold” at which decentralized VCCs may shift toward more centralized designs is a theoretical proposition; emphasize that this was not empirically established in the current study.

• The question of whether centralized VCCs deliver faster assistance should be clarified: does this concern arise from participant accounts or conceptual critique?

• The Discussion mentions that literature is inconclusive about power-sharing in VCCs, but cited studies address telemonitoring more broadly. Clarify this distinction.

• The text alternates between explanations based on patient empowerment and selection bias; these concepts should be disentangled and tied explicitly to empirical findings.

• Claims about threats to relational continuity, particularly in centralized VCCs, should be directly linked to the findings.

• Consider suggesting how relational continuity could be improved within centralized VCCs.

6. Limitations and Future Research

• The first limitation states that patient perspectives were not captured, but this was not an aim of the study; clarify the relevance of this limitation. Also note that only one theme could be validated with patient perspectives.

• Clarify how professional role differences (lines 544–546) may have shaped the findings—for example, nurses and medical specialists may have different views on collaboration.

• While the limitation of including only 3 out of 10 VCCs is acknowledged, its implications for transferability and contextual variation should be explained. Access challenges should also be described in the Methods.

• The absence of hybrid VCC models is noted but not introduced earlier in the manuscript; consider discussing or defining hybrid models earlier.

**********

6. PLOS authors have the option to publish the peer review history of their article (what does this mean?). If published, this will include your full peer review and any attached files.

**Do you want your identity to be public for this peer review?**  If you choose “no”, your identity will remain anonymous but your review may still be made public.

For information about this choice, including consent withdrawal, please see our Privacy Policy.

Reviewer #1: No

Reviewer #2: No

Reviewer #3: No

**********

[NOTE: If reviewer comments were submitted as an attachment file, they will be attached to this email and accessible via the submission site. Please log into your account, locate the manuscript record, and check for the action link "View Attachments". If this link does not appear, there are no attachment files.]

**Figure resubmission:**

While revising your submission, we strongly recommend that you use PLOS’s NAAS tool (https://ngplosjournals.pagemajik.ai/artanalysis) to test your figure files. NAAS can convert your figure files to the TIFF file type and meet basic requirements (such as print size, resolution), or provide you with a report on issues that do not meet our requirements and that NAAS cannot fix.

After uploading your figures to PLOS’s NAAS tool - https://ngplosjournals.pagemajik.ai/artanalysis, NAAS will process the files provided and display the results in the "Uploaded Files" section of the page as the processing is complete. If the uploaded figures meet our requirements (or NAAS is able to fix the files to meet our requirements), the figure will be marked as "fixed" above. If NAAS is unable to fix the files, a red "failed" label will appear above. When NAAS has confirmed that the figure files meet our requirements, please download the file via the download option, and include these NAAS processed figure files when submitting your revised manuscript.

**Reproducibility:**

To enhance the reproducibility of your results, we recommend that authors of applicable studies deposit laboratory protocols in protocols.io, where a protocol can be assigned its own identifier (DOI) such that it can be cited independently in the future. Additionally, PLOS ONE offers an option to publish peer-reviewed clinical study protocols. Read more information on sharing protocols at https://plos.org/protocols?utm_medium=editorial-email&utm_source=authorletters&utm_campaign=protocols

Attachment Submitted filename: PDIG-D-25-00451 (2)_Rev.pdf

# Author comment: Author response to Decision Letter 1

**DOI:** 10.1371/journal.pdig.0001479.r002

21 Jan 2026

Attachment Submitted filename: 20260121 Response letter PLOS.docx

# Peer review: Decision Letter 1

**DOI:** 10.1371/journal.pdig.0001479.r003

**Authors:** Laura Maaß, Zhao Ni, Laura Maaß, Zhao Ni

29 Mar 2026

PDIG-D-25-00451R1

The organization of virtual care centers: A qualitative study in Dutch hospitals

PLOS Digital Health

Dear Dr. Peters,

Thank you for submitting your manuscript to PLOS Digital Health. After careful consideration, we feel that it has merit but does not fully meet PLOS Digital Health's publication criteria as it currently stands. Therefore, we invite you to submit a revised version of the manuscript that addresses the points raised during the review process.

Please submit your revised manuscript by Apr 28 2026 11:59PM. If you will need more time than this to complete your revisions, please reply to this message or contact the journal office at digitalhealth@plos.org. When you're ready to submit your revision, log on to https://www.editorialmanager.com/pdig/ and select the 'Submissions Needing Revision' folder to locate your manuscript file.

Please include the following items when submitting your revised manuscript:

* A letter that responds to each point raised by the editor and reviewer(s). You should upload this letter as a separate file labeled 'Response to Reviewers'. This file does not need to include responses to any formatting updates and technical items listed in the 'Journal Requirements' section below.

* A marked-up copy of your manuscript that highlights changes made to the original version. You should upload this as a separate file labeled 'Revised Manuscript with Track Changes'.

* An unmarked version of your revised paper without tracked changes. You should upload this as a separate file labeled 'Manuscript'.

If you would like to make changes to your financial disclosure, competing interests statement, or data availability statement, please make these updates within the submission form at the time of resubmission. Guidelines for resubmitting your figure files are available below the reviewer comments at the end of this letter.

We look forward to receiving your revised manuscript.

Kind regards,

Laura Maaß

Academic Editor

PLOS Digital Health

Zhao Ni

Section Editor

PLOS Digital Health

Leo Anthony Celi

Editor-in-Chief

PLOS Digital Health

orcid.org/0000-0001-6712-6626

**Journal Requirements:**

If the reviewer comments include a recommendation to cite specific previously published works, please review and evaluate these publications to determine whether they are relevant and should be cited. There is no requirement to cite these works unless the editor has indicated otherwise.

**Additional Editor Comments (if provided):**

[Note: HTML markup is below. Please do not edit.]

**Reviewers' Comments:**

Reviewer's Responses to Questions

**Comments to the Author**

1. If the authors have adequately addressed your comments raised in a previous round of review and you feel that this manuscript is now acceptable for publication, you may indicate that here to bypass the “Comments to the Author” section, enter your conflict of interest statement in the “Confidential to Editor” section, and submit your "Accept" recommendation.

Reviewer #1: All comments have been addressed

Reviewer #2: All comments have been addressed

Reviewer #3: All comments have been addressed

**********

2. Does this manuscript meet PLOS Digital Health’s publication criteria? Is the manuscript technically sound, and do the data support the conclusions? The manuscript must describe methodologically and ethically rigorous research with conclusions that are appropriately drawn based on the data presented.

Reviewer #1: Yes

Reviewer #2: Yes

Reviewer #3: Yes

**********

3. Has the statistical analysis been performed appropriately and rigorously?

Reviewer #1: N/A

Reviewer #2: Yes

Reviewer #3: Yes

**********

4. Have the authors made all data underlying the findings in their manuscript fully available (please refer to the Data Availability Statement at the start of the manuscript PDF file)?

The PLOS Data policy requires authors to make all data underlying the findings described in their manuscript fully available without restriction, with rare exception. The data should be provided as part of the manuscript or its supporting information, or deposited to a public repository. For example, in addition to summary statistics, the data points behind means, medians and variance measures should be available. If there are restrictions on publicly sharing data—e.g. participant privacy or use of data from a third party—those must be specified.

Reviewer #1: (No Response)

Reviewer #2: Yes

Reviewer #3: Yes

**********

5. Is the manuscript presented in an intelligible fashion and written in standard English?

PLOS Digital Health does not copyedit accepted manuscripts, so the language in submitted articles must be clear, correct, and unambiguous. Any typographical or grammatical errors should be corrected at revision, so please note any specific errors here.

Reviewer #1: Yes

Reviewer #2: Yes

Reviewer #3: Yes

**********

6. Review Comments to the Author

Please use the space provided to explain your answers to the questions above. You may also include additional comments for the author, including concerns about dual publication, research ethics, or publication ethics. (Please upload your review as an attachment if it exceeds 20,000 characters)

**Reviewer #1:**  Thank you for the opportunity to review the revised version of this manuscript. The authors have clearly taken the previous comments seriously and have responded to them in a thoughtful and constructive manner.

Overall, the revisions have significantly improved the manuscript. The conceptual framing is clearer, the internal consistency of the study aim and discussion has been strengthened, and the methodological transparency has been enhanced. In particular, the clarifications regarding terminology, the positioning of the study, and the articulation of implications have made the argumentation more coherent and easier to follow.

While in some instances the additional elaborations could perhaps have been developed in greater depth, I acknowledge the constraints of word limits and consider that the authors have made a sincere and substantial effort to address the concerns raised in the previous review. The manuscript is now clearer, more internally consistent, and better positioned within the broader scholarly conversation.

In my view, the paper has improved meaningfully through the revision process and is suitable for publication. Recommendation: Accept.

**Reviewer #2:**  My initial review concerns have been fully addressed.

**Reviewer #3:**  The revised manuscript demonstrates clear and substantive improvements. In particular, the conceptual distinction between centralised and decentralised VCC designs is now more consistently integrated across the Background, Methods and Results sections, and the Discussion has been strengthened in terms of structure and analytical coherence. The authors have responded constructively to most substantive concerns.

However, several issues remain that require clarification in order to ensure methodological transparency and conceptual robustness. These relate to the theoretical anchoring of the study, the reporting of FTE data, the description of the coding process, and the explicit reporting of interviewer positionality in the main text.

Specific comments

Q25

Although additional references to organisational design theory have been incorporated, the manuscript still lacks a clearly articulated analytical framework. The distinction between centralised and decentralised VCC designs remains largely descriptive rather than explicitly grounded in established theoretical dimensions (e.g. coordination mechanisms, governance structures, standardisation versus mutual adjustment). The authors should more clearly specify how their empirical findings are theoretically informed and how they contribute to organisational design theory beyond citation.

Q32

The presentation of “FTE working in VCC (n)” requires clarification. It appears unlikely that all staff members are employed at 1.0 FTE within the VCC, and the number of employees and the number of FTEs represent conceptually distinct parameters. The authors should clarify whether these figures reflect aggregated FTE calculations or simply the number of staff, and explicitly explain how the FTE values were derived.

Q33

While reference is made to COREQ in the supplementary material, essential information regarding the interviewer(s) should also be included in the main text. The manuscript should explicitly state who conducted the interviews, their professional background, whether they had prior relationships with participants, and how potential bias or positionality was addressed. Reliance solely on supplementary reporting is insufficient for full methodological transparency.

Q34

The description of the thematic analysis remains too general. The statement that coding consistency was ensured through peer meetings does not sufficiently clarify whether double coding was conducted, who performed the coding, how disagreements were resolved, or whether a formal codebook was developed. A more detailed account of the coding process is required to strengthen methodological rigour and transparency.

Q37

The manuscript states that secondary data (e.g. brochures and policy documents) were collected and used to support interpretation. However, it remains unclear how these documents were analysed, whether they were coded alongside the interview data, and how they contributed to the development of themes. The authors should specify the analytical approach applied to the document analysis and clarify how (and where) these data are reflected in the Results. Without such clarification, the role of document analysis in the overall study design remains insufficiently transparent.

**********

7. PLOS authors have the option to publish the peer review history of their article (what does this mean?). If published, this will include your full peer review and any attached files.

**Do you want your identity to be public for this peer review?**  If you choose “no”, your identity will remain anonymous but your review may still be made public.

For information about this choice, including consent withdrawal, please see our Privacy Policy.

Reviewer #1: No

Reviewer #2: No

Reviewer #3: No

**********

[NOTE: If reviewer comments were submitted as an attachment file, they will be attached to this email and accessible via the submission site. Please log into your account, locate the manuscript record, and check for the action link "View Attachments". If this link does not appear, there are no attachment files.]

**Figure resubmission:**

While revising your submission, we strongly recommend that you use PLOS’s NAAS tool (https://ngplosjournals.pagemajik.ai/artanalysis) to test your figure files. NAAS can convert your figure files to the TIFF file type and meet basic requirements (such as print size, resolution), or provide you with a report on issues that do not meet our requirements and that NAAS cannot fix.

After uploading your figures to PLOS’s NAAS tool - https://ngplosjournals.pagemajik.ai/artanalysis, NAAS will process the files provided and display the results in the "Uploaded Files" section of the page as the processing is complete. If the uploaded figures meet our requirements (or NAAS is able to fix the files to meet our requirements), the figure will be marked as "fixed" above. If NAAS is unable to fix the files, a red "failed" label will appear above. When NAAS has confirmed that the figure files meet our requirements, please download the file via the download option, and include these NAAS processed figure files when submitting your revised manuscript.

**Reproducibility:**

To enhance the reproducibility of your results, we recommend that authors of applicable studies deposit laboratory protocols in protocols.io, where a protocol can be assigned its own identifier (DOI) such that it can be cited independently in the future. Additionally, PLOS ONE offers an option to publish peer-reviewed clinical study protocols. Read more information on sharing protocols at https://plos.org/protocols?utm_medium=editorial-email&utm_source=authorletters&utm_campaign=protocols

# Author comment: Author response to Decision Letter 2

**DOI:** 10.1371/journal.pdig.0001479.r004

22 Apr 2026

Attachment Submitted filename: 20260421 Response letter.docx

# Peer review: Decision Letter 2

**DOI:** 10.1371/journal.pdig.0001479.r005

**Authors:** Laura Maaß, Zhao Ni, Laura Maaß, Zhao Ni, Laura Maaß, Zhao Ni

18 May 2026

The organization of virtual care centers: A qualitative study in Dutch hospitals

PDIG-D-25-00451R2

Dear Mr. Peters,

We are pleased to inform you that your manuscript 'The organization of virtual care centers: A qualitative study in Dutch hospitals' has been provisionally accepted for publication in PLOS Digital Health.

Before your manuscript can be formally accepted you will need to complete some formatting changes, which you will receive in a follow-up email from a member of our team.

Please note that your manuscript will not be scheduled for publication until you have made the required changes, so a swift response is appreciated.

IMPORTANT: The editorial review process is now complete. PLOS will only permit corrections to spelling, formatting or significant scientific errors from this point onwards. Requests for major changes, or any which affect the scientific understanding of your work, will cause delays to the publication date of your manuscript.

If your institution or institutions have a press office, please notify them about your upcoming paper to help maximize its impact. If they'll be preparing press materials, please inform our press team as soon as possible -- no later than 48 hours after receiving the formal acceptance. Your manuscript will remain under strict press embargo until 2 pm Eastern Time on the date of publication. For more information, please contact digitalhealth@plos.org.

Thank you again for supporting Open Access publishing; we are looking forward to publishing your work in PLOS Digital Health.

Best regards,

Laura Maaß, PhD

Academic Editor

PLOS Digital Health

***********************************************************

**Additional Editor Comments (if provided):**

**Reviewer Comments (if any, and for reference):**

Reviewer's Responses to Questions

**Comments to the Author**

1. If the authors have adequately addressed your comments raised in a previous round of review and you feel that this manuscript is now acceptable for publication, you may indicate that here to bypass the “Comments to the Author” section, enter your conflict of interest statement in the “Confidential to Editor” section, and submit your "Accept" recommendation.

Reviewer #3: All comments have been addressed

**********

2. Does this manuscript meet PLOS Digital Health’s publication criteria? Is the manuscript technically sound, and do the data support the conclusions? The manuscript must describe methodologically and ethically rigorous research with conclusions that are appropriately drawn based on the data presented.

Reviewer #3: Yes

**********

3. Has the statistical analysis been performed appropriately and rigorously?

Reviewer #3: Yes

**********

4. Have the authors made all data underlying the findings in their manuscript fully available (please refer to the Data Availability Statement at the start of the manuscript PDF file)?

The PLOS Data policy requires authors to make all data underlying the findings described in their manuscript fully available without restriction, with rare exception. The data should be provided as part of the manuscript or its supporting information, or deposited to a public repository. For example, in addition to summary statistics, the data points behind means, medians and variance measures should be available. If there are restrictions on publicly sharing data—e.g. participant privacy or use of data from a third party—those must be specified.

Reviewer #3: Yes

**********

5. Is the manuscript presented in an intelligible fashion and written in standard English?

PLOS Digital Health does not copyedit accepted manuscripts, so the language in submitted articles must be clear, correct, and unambiguous. Any typographical or grammatical errors should be corrected at revision, so please note any specific errors here.

Reviewer #3: Yes

**********

6. Review Comments to the Author

Please use the space provided to explain your answers to the questions above. You may also include additional comments for the author, including concerns about dual publication, research ethics, or publication ethics. (Please upload your review as an attachment if it exceeds 20,000 characters)

Reviewer #3: All my discussion points are adressed. I suggest accepting this paper for publication.

**********

7. PLOS authors have the option to publish the peer review history of their article (what does this mean?). If published, this will include your full peer review and any attached files.

**Do you want your identity to be public for this peer review?**  If you choose “no”, your identity will remain anonymous but your review may still be made public.

For information about this choice, including consent withdrawal, please see our Privacy Policy.

Reviewer #3: No

**********
